# RhoMitoAnnotator and Polypods, Bioinformatics Tools for the *Rhodiola* Mitochondrial Gene Assembly, Annotation and Phylogenetic Analysis

**DOI:** 10.3390/ijms27104440

**Published:** 2026-05-15

**Authors:** Erhuan Zang, Yanda Zhu, Tingyu Ma, Dengxiu Ma, Lingchao Zeng, Xiaozhe Yi, Peigen Xiao, Lijia Xu, Linchun Shi, Jinxin Liu

**Affiliations:** 1 State Key Laboratory for Quality Ensurance and Sustainable Use of Dao-di Herbs, Institute of Medicinal Plant Development, Chinese Academy of Medical Sciences and Peking Union Medical College, Beijing 100193, China; zangerhuan@implad.ac.cn (E.Z.); s2024009062@student.pumc.edu.cn (Y.Z.); 18283449576@163.com (D.M.); zlcryl@163.com (L.Z.); pgxiao@implad.ac.cn (P.X.); xulijia@hotmail.com (L.X.); 2 School of Pharmacy, Minzu University of China, Beijing 100081, China; tyma0904@163.com; 3 School of Traditional Chinese Medicine, Capital Medical University, Beijing 100069, China; yxz1996@126.com; 4 Engineering Research Center of Chinese Medicine Resource, Ministry of Education, Beijing 100193, China

**Keywords:** RhoMitoAnnotator, Polypods, mitochondrial genome, gene complexity, *Rhodiola*

## Abstract

Plant mitochondrial genomes are difficult to analyze because of their structural dynamism and frequent annotation errors. To address these challenges, we first constructed a high-confidence mitochondrial reference library for *Rhodiola* by integrating transcriptomic evidence, public sequence resources, and experimental validation. This curated resource defined 30 mitochondrial protein-coding genes (PCGs), including corrected exon–intron boundaries and validated 5′-terminal variants in *ccmC*, *ccmFn*, and *nad9*. Leveraging this curated dataset, we developed the RhoMitoAnnotator, which integrates three novel algorithms, EBAnno, REAnno, and NCAnno, to accurately annotate trans-splicing, RNA editing, and non-canonical start/stop codons. Using long-read sequencing guided by the RhoMitoAnnotator, we completed the mitogenomes of *R. rosea*, *R. crenulata*, and *R. sacra*, systematically re-annotated seven publicly available mitogenomes, revealing cross-chromosomal gene arrangement, and widespread structural misannotations. To enable scalable analysis with short-read data, we built Polypods, an integrated pipeline that successfully assembled mitochondrial PCGs from 108 samples across 39 *Rhodiola* species, and identified variant genes, stop codon-lacking regions in *nad6*, and internal stop codons in *rpl16*. Phylogenetic analyses based on mitochondrial and chloroplast PCGs showed a lineage pattern consistent with the hypothesis of an evolutionary transition from hermaphroditism to dioecy in *Rhodiola*, and consistently supported six species as monophyletic lineages. Overall, this study provides a curated mitochondrial gene atlas for *Rhodiola* and a reference-guided analytical framework for mitochondrial PCG annotation and recovery in this genus, with potential adaptability to other plant lineages after lineage-specific database construction and parameter optimization.

## 1. Introduction

Mitochondrial genomes (mitogenomes), as the central mediators of cellular energy metabolism, play an irreplaceable role in revealing species evolutionary mechanisms, analyzing adaptive evolution, and tracing population historical dynamics [1]. There is a significant size difference between mitogenomes of plants and animals, with plant mitogenomes typically being 100 to 10,000 times larger than those of animals. In angiosperms, mitogenome sizes generally range from 200 Kb to 3 Mb [2]. In contrast to chloroplast genomes, plant mitogenomes exhibit remarkable variability both between and within species [3]. Plant mitogenomes exhibit several distinctive characteristics, including vast variation in size and structure, highly conserved genes, sparse gene distribution, the presence of trans-spliced genes, and widespread RNA editing [4]. This structural complexity presents two major challenges. First, annotation errors and gene omissions are widespread in public plant mitogenome databases, significantly compromising data reliability. Second, the accurate and scalable acquisition of protein-coding genes (PCGs) remains difficult [5]. Although third-generation long-read sequencing technologies such as PacBio and Oxford Nanopore can assemble complete sequences, their high cost restricts their use in large-scale comparative studies. In contrast, next-generation sequencing (NGS) technologies, exemplified by Illumina, offer advantages in cost-efficiency and accuracy, but their short read lengths typically result in highly fragmented mitochondrial contigs [6]. This predicament has resulted in mitogenome research lagging far behind chloroplast genome studies in scale. As of 26 November 2025, the National Center for Biotechnology Information (NCBI) has released 12,989 chloroplast genomes, but only 688 mitochondrial genomes (https://www.ncbi.nlm.nih.gov/datasets/organelle/?taxon=33090, accessed on 26 November 2025), highlighting a substantial disparity. Currently, most studies on plant mitochondrial genes utilize PCGs for phylogenetic and evolutionary analyses without requiring complete genome assemblies. Notably, fragmented assemblies derived from NGS data can still contain complete PCG sequences [7]. Therefore, developing efficient tools capable of accurate PCG annotation and enabling scalable research based on NGS data has become crucial for advancing the field of plant mitochondrial genomics.

To address these common challenges, this study focuses on the genus *Rhodiola* (Crassulaceae) as a case model. *Rhodiola* comprises approximately 90 species worldwide, with 73 species, 2 subspecies, and 7 varieties distributed in China, representing a rapidly radiating population whose intrageneric phylogenetic relationships remain incompletely resolved [8]. Species within this genus are of notable medicinal value, *R. crenulata* and *R. rosea* are included in the United States Pharmacopeia (USP-NF2021), while *R. crenulata* and *R. sacra* are listed in the 2025 edition of the Chinese Pharmacopoeia [9]. Currently, although chloroplast genomes of about 42 *Rhodiola* species have been systematically studied, mitochondrial genomic data remain extremely limited; only seven species are available in the NCBI database, many of which exhibit problems such as gene omissions and annotation inaccuracies [10,11]. This scarcity and low quality of mitochondrial genomic data significantly hinder a deeper understanding of mitochondrial evolutionary patterns and phylogenetic relationships within *Rhodiola*. Therefore, a systematic investigation of annotation errors and large-scale gene acquisition in this genus is needed not only to elucidate the evolutionary history of its mitochondrial genes but also to establish an analytical framework with potential transferability to other plant groups with complex mitogenomes, provided that lineage-specific references and parameters are carefully calibrated.

Addressing the widespread issues of annotation errors and omissions in plant mitochondrial genes within public databases, we first clarified the exact number and precise boundaries of PCGs in *Rhodiola*, generating a high-quality reference sequence database. Then, we developed the annotation tool RhoMitoAnnotator (v1.0.0), incorporating algorithms such as EBAnno, REAnno, and NCAnno, to effectively handle trans-splicing, RNA editing, and non-canonical start/stop codons, significantly improving annotation accuracy and completeness. Utilizing this tool with second- and third-generation sequencing data, we generated complete mitochondrial genome maps for three medicinally important *Rhodiola* species: *R. rosea*, *R. crenulata*, and *R. sacra*. Furthermore, we re-annotated the mitogenomes of seven *Rhodiola* species available in public databases and performed collinearity analysis. To reduce reliance on costly third-generation long-read sequencing, we developed Polypods, a bioinformatic pipeline designed for Illumina short-read data. With its modular and extensible architecture, Polypods integrates the complete workflow from PCG assembly and gene annotation to phylogenetic analysis, providing an efficient, low-cost, and standardized solution for plant mitochondrial PCGs study. Applying this pipeline along with the curated reference sequences database, we successfully assembled and annotated mitochondrial PCGs from 108 samples of 39 *Rhodiola* species and performed population genomic analyses. Phylogenetic trees constructed from mitochondrial and chloroplast genomes were compared, revealing both convergent evolutionary signals related to sexual systems and topological conflicts in certain clades. This study not only provides essential tools and genomic resources for elucidating organellar gene evolution in *Rhodiola*, but also provides a reference-guided analytical framework that may be adapted to other plant lineages after the construction of lineage-specific reference databases and parameter optimization.

## 2. Results

### 2.1. Correction of Mitochondrial Gene Sequences Based on Transcriptomic and PCR Evidence

Constructing a reliable *Rhodiola* mitochondrial reference gene database is essential for accurate annotation of mitochondrial PCGs. In this section, we present the correction and curation of mitochondrial gene sequences based on integrated transcriptomic, public sequence, and PCR evidence. These analyses clarified the conserved PCG set in *Rhodiola* mitogenomes, identified previously omitted genes and putative pseudogenes, refined CDS boundaries and exon structures, and supported RNA-editing site detection. Together, these results provided a curated mitochondrial reference gene database for subsequent annotation and comparative analyses.

#### 2.1.1. Confirmation of PCGs in the Mitogenomes of Rhodiola Species

Through the integration of transcriptome data and homologous alignment analysis, the mitochondrial PCGs in *Rhodiola* were determined to comprise 30. Due to omissions in the assembly or annotation process, five previously omitted PCGs were identified in *R. sacra*, namely *atp9*, *nad1*, *nad5*, *nad6*, and *rpl16*. In *R. crenulata*, nine previously omitted PCGs were discovered, including *atp9*, *ccmFn*, *cob*, *cox2*, *nad1*, *nad3*, *rpl16*, *rps12*, and *rps7*. These genes possess complete open reading frames (ORFs), and their transcripts are well-supported by the transcriptome data generated in this study, indicating their functional potential as PCGs (Supplementary Table S1). Additionally, we constructed a database from the ONT long-read transcriptome data of *R. sacra* using makeblastdb, and performed BLASTn alignment with *Rhodiola* mitochondrial *rps4* and *rps14* gene sequences from public databases. The results showed no expression evidence for these genes in the transcriptome data. No transcript evidence for mitochondrial *rps4* and *rps14* was detected in the ONT long-read transcriptome dataset of *R. sacra*. Therefore, these loci were treated as putative pseudogenes or non-functional remnants in *Rhodiola* mitogenome [12]. This interpretation remains based on the absence of transcript support and sequence evidence, and further validation would be required to determine their complete loss or functional status.

#### 2.1.2. Curation of 5′/3′ Boundaries of PCGs in Rhodiola

Multiple sequence alignment among *Rhodiola* species revealed variations in the 5′ and 3′ boundaries of certain mitochondrial genes. A total of 19 genes, including *atp1*, *atp4*, *atp8*, *atp9*, *ccmB*, *cox1*, *cox2*, *cox3*, *nad1*, *nad3*, *nad4*, *nad4L*, *nad5*, *nad7*, *rpl10*, *rpl5*, *rps12*, *rps13*, and *rps7* were retained with unmodified boundaries following confirmation of clear and consistent alignment signals. For the 11 genes (*atp6*, *matR*, *ccmC*, *ccmFn*, *ccmFc*, *cob*, *mttB*, *nad2*, *nad6*, *nad9*, *rpl16*) whose boundaries could not be unambiguously defined due to inconsistent alignment results, precise CDS boundaries were resolved by integrating multiple lines of evidence, including experimental data from the literature and homologous protein sequences from the UniProt database. The 5′ sites of *nad2* and *nad9* were determined based on experimentally validated data reported in previous studies [13,14], while their 3′ sites were highly conserved in *Rhodiola* species. For *ccmC*, *ccmFc*, *ccmFn*, *mttB*, *cob*, and *rpl16*, the boundaries of the coding regions were verified by comparing the homologous protein sequences of *Arabidopsis thaliana* in the UniProt database. Furthermore, the 3′ end of *atp6* was identified by comparison with experimental data from the literature. However, its 5′ end showed length variations relative to the reference sequence. Mapping the *R. sacra* full-length transcriptome data with the *A. thaliana atp6* sequence also revealed poor mapping efficiency at the 5′ end. Consequently, we selected the *atp6* sequence from *R. rosea*, which exhibited good conservation in multiple sequence alignment and maintained the ORF with an ATG start codon, as the 5′ boundary of *atp6*. The annotation of *matR* proved more complex. Compared with the homologous protein in *A. thaliana*, *matR* in *Rhodiola* lacks a termination codon at the same 3′ end but extends downstream by several amino acids. Accordingly, we chose the sequence with the longest ORF with a recognizable termination codon as the 3′ boundary of *matR* from *R. rosea*. At the 5′ end of *matR*, although putative homologous regions align to the *A. thaliana* protein, they initiate with a non-canonical ATA start codon and display substantial nucleotide divergence, suggesting potential lineage-specific evolutionary changes. Following the approach used for determining the atp6 5′ end, we selected the *matR* sequence from *R. rosea* that was well-conserved in multiple alignment and preserved the original ORF with an ATG start codon as the 5′ boundary of *matR*. Notably, the 5′ boundaries of *nad6* were relatively conserved within the genus, but conspicuous misalignment was observed after approximately 619 bp. Comparison with the cloned *nad6* sequence from *Brassica campestris* indicated a frameshift mutation in *Rhodiola* resulting from a single-nucleotide deletion [15]. Consequently, the typical TAA stop codon is not formed, leading to extended translation. Therefore, the 3′ boundaries of *nad6* were determined based on the longest identifiable ORF. During alignment, we noted that the 5′ end of *ccmFn* and *nad9* in *R. crenulata* and *ccmC* in *R. rosea* were inconsistent with their respective assembled transcripts. Moreover, potential C-to-U RNA editing was observed at putative start and stop codons: the 5′ ends of *nad1* and *nad4L* began with ACG, and the 3′ end of *atp6* was CAA. These observations require further validation to confirm their biological relevance.

#### 2.1.3. Validation of 5′ Terminal Variants by PCR

**Figure 1:**
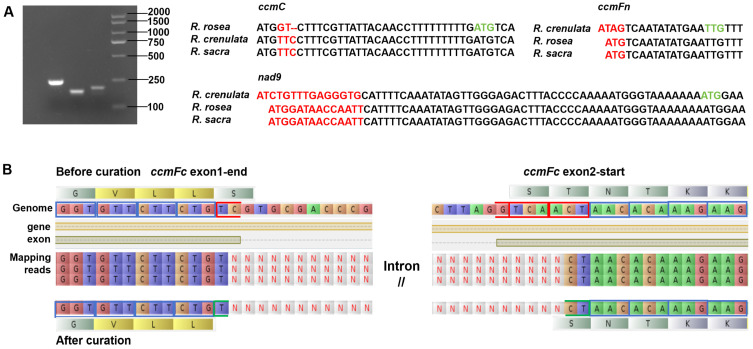
Correction of mitochondrial PCG annotations based on transcriptomic and PCR evidence: (**A**) PCR validation of 5′-terminal variations in *ccmC*, *ccmFn*, and *nad9*. Variable sequences among the three species are highlighted in red, and start codons are highlighted in green. (**B**) Transcript-supported correction of the *ccmFc* exon boundary in *R. sacra* and *R. crenulata*. Blue, red, and green boxes represent original codons, misannotated codons, and corrected codons, respectively. The original protein sequence, transcript read mapping, and corrected protein sequence are shown from top to bottom. "//" indicates that the sequence in between is an intron sequence.

Multiple sequence alignment revealed significant variations in the 5′ terminal regions of the *ccmC* gene in *R. rosea* and the *ccmFn* and *nad9* genes in *R. crenulata*. To confirm the biological validity of these variations, we performed PCR amplification and Sanger sequencing targeting the 5′ regions of these three genes. To encompass the 5′ terminal variant regions, the amplification products of *ccmC*, *ccmFn*, and *nad9* are 206 bp, 175 bp, and 200 bp, respectively (Figure 1A). The results showed complete concordance between the assembled sequences and the PCR-derived sequences, experimentally validating the authenticity of the observed 5′ terminal variants. Based on ORF prediction and sequence conservation analysis, we propose the following interpretation: the 5′ end of the *ccmC* gene in *R. rosea* is extended by 34 nucleotides downstream compared to the reference sequence, utilizes an ATG start codon, and encodes a putative protein with an N-terminal truncation of 11 amino acids. In *R. crenulata*, the *ccmFn* gene exhibits a 16-nucleotide downstream extension at its 5′ end, utilizes a TTG start codon, and results in an N-terminal truncation of five amino acids. Similarly, the *nad9* gene in *R. crenulata* displays a 64-nucleotide downstream extension at the 5′ end, initiates with an ATG start codon, and leads to an N-terminal truncation of 21 amino acids. These experimentally validated 5′ terminal variations indicate that mitochondrial genes in the genus *Rhodiola* may achieve functional modulation or undergo adaptive evolution through N-terminal protein truncation. The biological significance of these structural alterations warrants further investigation.

#### 2.1.4. Analysis of RNA Editing Sites

RNA editing sites were analyzed in *R. rosea*, *R. crenulata*, and *R. sacra* by comparing genomic and transcriptomic data with PREPACT 3.0 [16]. Analysis results show that a total of 524 RNA editing sites were identified in *R. rosea*, affecting 509 amino acid residues, among which 36 were synonymous edits. In *R. crenulata*, 490 editing sites were detected, altering 478 amino acids, including 43 synonymous changes. *R. sacra* exhibited 475 editing sites, impacting 464 amino acids, with 29 being synonymous (Supplementary Tables S2–S4). All identified editing events were of the C-to-U type, with synonymous edits accounting for 7.1% to 9.0% of the total. Notably, transcriptomic data confirmed RNA editing events affecting both start and stop codons. The initiation codons of *nad1* and *nad4L* were edited from ACG to ATG, enabling proper translation initiation, whereas the stop codon of *atp6* was modified from CAA to TAA, ensuring accurate translation termination. A particularly notable finding was a high-frequency C-to-U editing in *rpl16-37*, observed in all three species with an editing rate of 95%, resulting in a codon change from CAG to UAG. According to the standard genetic code, UAG functions as a stop codon, which would presumably lead to the production of a truncated protein. The downstream GTG codon at *rpl16-40* may serve as an alternative start site. Considerable variation in editing patterns was observed among species. The *mttB* gene displayed the most pronounced divergence, with 28 editing sites in *R. rosea*, 18 in *R. crenulata*, and only 2 in *R. sacra*. Similarly, the *atp9* gene exhibited species-specific editing, with a single synonymous edit at position 93 bp detected exclusively in *R. rosea*. In addition, *rpl10* and *cob* genes showed editing events unique to *R. crenulata*. Specifically, C-to-U editing in *rpl10-228*, *rpl10-330* and *cob-53* was found only in this species. These results suggest that species-specific RNA editing patterns may reflect post-transcriptional regulatory divergence within *Rhodiola*.

#### 2.1.5. Curation of Exon–Intron Boundaries

Using Illumina rRNA-depleted RNA-seq reads that were mapped to the genomes of *R. crenulata* and *R. sacra*, we performed a systematic correction of the exon boundaries of seven multi-exon mitochondrial genes annotated in the NCBI database for these two species. By referencing the mitogenome of *R. rosea* assembled in this study, we confirmed the following exon compositions in *Rhodiola*: *nad1*, *nad2*, *nad5*, and *nad7* each consist of five exons; *nad4* contains four exons; *cox2* has three exons; and *ccmFc* comprises two exons. Comparative analysis revealed substantial assembly and annotation errors in the NCBI records for *R. crenulata* and *R. sacra*. Specifically, *R. crenulata* exhibited three assembly omissions, five annotation omissions, and eight exon annotation errors, while *R. sacra* had nine assembly omissions, three annotation omissions, and seven exon annotation errors (Supplementary Table S5). Notable exon boundary misannotations were identified in *R. crenulata* for the genes *nad1*, *nad2*, *nad5*, *cox2*, and *ccmFc*, and in *R. sacra* for *nad1*, *nad2*, *nad4*, *nad5*, and *ccmFc* (Supplementary Figure S1). A representative example involves the boundary between exon1-end and exon 2-start of *ccmFc* in both *R. sacra* and *R. crenulata*. The originally annotated protein sequence “GVLLSSTNTKK” was corrected to “GVLL--SNTKK” based on transcriptional evidence (Figure 1B). Following systematic correction, exon boundaries of multi-exon genes were consistently supported by transcriptomic data, significantly improving the accuracy and reliability of mitochondrial gene annotations in these species. These corrected gene models provided a high-quality reference sequence database for RhoMitoAnnotator annotation and Polypods-based PCG recovery.

### 2.2. Development of RhoMitoAnnotator for Precise Mitochondrial PCG Annotation

**Figure 2:**
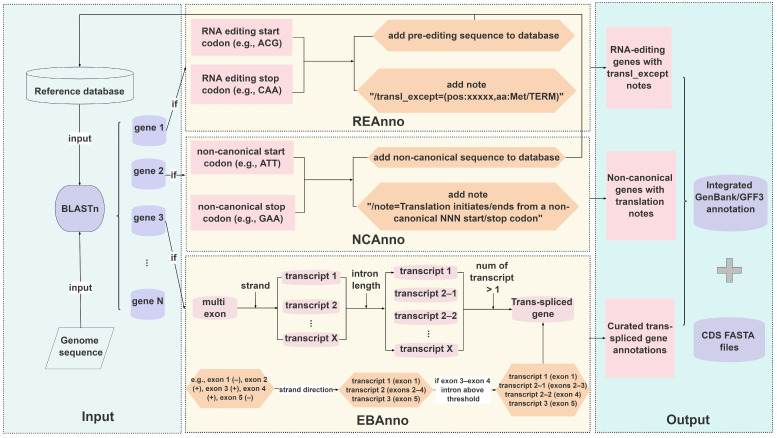
Overview of the RhoMitoAnnotator workflow. The pipeline involves three novel algorithms, EBAnno, REAnno, and NCAnno, to accurately annotate trans-splicing events, RNA editing sites, and non-canonical start/stop codons.

To address the widespread complexity in mitochondrial gene structures and meet the need for accurate annotation, this study developed the RhoMitoAnnotator, a mitochondrial PCG annotation tool for the genus *Rhodiola*. The tool employs BLAST to align the genome to reference sequence databases for both single-exon and multi-exon genes. Custom algorithms were implemented to handle the intricacies of multi-exon gene structures and non-canonical initiation/termination events, enabling accurate annotation of PCGs in *Rhodiola* mitogenomes (Figure 2).

To resolve complex multi-exon gene structures, particularly those involving trans-splicing events, we developed the Exon-Based Annotation (EBAnno) algorithm. First, the algorithm compiles the genomic coordinates and strand directionality for all exons. If exons are located on opposing strands, the transcripts are split, defining exons oriented on the same strand as belonging to a single transcript. For example, a gene with five exons arranged as “exon 1 (+), exon 2 (−), exon 3 (−), exon 4 (−), exon 5 (+)” would be divided into three transcripts: transcript 1 (exon 1), transcript 2 (exons 2–4), and transcript 3 (exon 5). For any transcript containing two or more exons, a second round of splitting is performed based on a configurable intron length threshold (Supplementary Table S6). If the distance between two adjacent exons exceeds this threshold, the transcript is further subdivided. In the above example, if the interval between exon 3 and exon 4 surpasses the set cutoff, transcript 2 would be split into transcript 2–1 (exons 2–3) and transcript 2–2 (exon 4). If a gene has multiple transcripts, it is marked as a trans-spliced gene (e.g., *nad1*, *nad2* and *nad5*). Additionally, any gene whose exon count deviates from the number recorded in the reference database is labeled as a pseudogene. To enhance annotation rigor, an exon order validation step was implemented. For two adjacent exons on the same strand, they are expected to follow the canonical genomic order (e.g., exon1 before exon2 on the positive strand, and exon2 before exon1 on the negative strand). Any reversal of this expected order leads to the gene being flagged as a pseudogene. Although no such mis-ordered cases were encountered in our annotations, this validation step remains available as an optional user-specified check.

To address discrepancies between translation predictions and actual transcript outcomes caused by RNA editing of start or stop codons (Supplementary Table S7), we developed the RNA Editing gene Annotation (REAnno) algorithm. This algorithm uses the pre-edited gene sequence as a reference and explicitly marks the editing sites along with their corresponding amino acid changes in the annotation output. For instance, in cases such as the editing of the *nad4L* start codon from “ACG” to “ATG”, the annotation indicates “/transl_except=(pos:xxxxx,aa:Met)” to indicate a post-transcriptional modification, and the translation is directly rendered as “M”. For genes utilizing non-canonical start/stop codons, we developed the non-canonical gene Annotation (NCAnno) algorithm. By specifying the “/note=Translation initiates from a non-canonical ATT start codon.” field, NCAnno ensures correct translational framework annotation for genes such as *cob* (start codon ATT). Regarding termination anomalies, for example, the absence of a stop codon in *nad6* or premature termination in *matR* due to poorly conserved 3′ ends, our strategy involves providing reference sequences of varying lengths and determining the termination boundary based on the sequence with the highest alignment score.

### 2.3. Reconstruction of the Mitogenome Database and Microsynteny Analysis in Rhodiola

During the process of gene curation, we identified widespread annotation errors and gene omissions in several published *Rhodiola* mitogenomes available in the NCBI database. To address these issues, we performed systematic re-annotation of seven *Rhodiola* species (*R. juparensis*, *R. tangutica*, *R. crenulata*, *R. sacra*, *R. wallichiana*, *R. kirilowii*, *R. rosea*) using the RhoMitoAnnotator, resulting in the establishment of a high-quality reference database for *Rhodiola* mitogenomes. After re-annotation, all seven species were confirmed to encode PCGs: *atp1*, *atp4*, *atp6*, *atp8*, *atp9*, *ccmB*, *ccmC*, *ccmFc*, *ccmFn*, *cob*, *cox1*, *cox2*, *cox3*, *matR*, *mttB*, *nad1*, *nad2*, *nad3*, *nad4*, *nad4L*, *nad5*, *nad6*, *nad7*, *nad9*, *rpl5*, *rpl10*, *rpl16*, *rps7*, *rps12*, and *rps13*. In addition to previously noted gene omissions in *R. crenulata* and *R. sacra* based on transcriptomic data, this analysis further revealed that *R. juparensis* lacks four PCGs (*atp9*, *nad1*, *nad5*, and *rpl16*), while *R. wallichiana* lacks *atp9*, *nad1*, *nad5*, *nad6*, and *rpl16*. The mitogenome assembly of *R. kirilowii* was found to be incomplete, resulting in the omission of 18 PCGs, including *atp4*, *atp8*, *atp9*, *ccmFn*, *cob*, *cox1*, *matR*, *mttB*, *nad1*, *nad2*, *nad4*, *nad4L*, *nad5*, *nad9*, *rpl5*, *rpl10*, *rpl16*, and *rps7.* In all species, 129 bp *sdh4* sequences were identified. It exhibits high sequence similarity to a homologous region annotated as a pseudogene in *A. thaliana*, supporting the classification of *sdh4* as a pseudogene in *Rhodiola*. We also observed widespread occurrences of non-canonical start and stop codons. Specifically, *nad1* and *nad4L* use ACG as the start codon in all species, and *atp6* ends with CAA. These sites were respectively converted into the standard AUG start codon and UAA stop codon through C-to-U RNA editing. The *cob* gene consistently employs ATT as the start codon across all species. Furthermore, species-specific variations were identified: in *R. tangutica*, the *atp8* start codon is altered from ATG to GTG, and in *R. wallichiana*, the *cox1* stop codon is changed from TAA to GGA. Additionally, the *atp8* gene in *R. juparensis* begins with ACG, suggesting it may also undergo RNA editing.

After annotating seven *Rhodiola* species with the RhoMitoAnnotator, we conducted microsynteny analyses to further investigate the distribution and evolutionary patterns of the PCGs in these species. We focused specifically on trans-spliced genes because they contain multiple exons and generally exhibit relatively flexible genomic arrangements (Supplementary Figure S2). We observed that several exons of *nad1* and *nad5* are present in multiple copies in *R. rosea* and *R. crenulata*, a phenomenon not detected in other species, suggesting that these two species may have undergone more frequent chromosomal rearrangements. Based on the multi-chromosome patterns observed in *R. crenulata* and *R. wallichiana*, exons belonging to the *nad1*, *nad2*, and *nad5* are located on different chromosomes. This arrangement suggests a unique trans-splicing mechanism of these genes in these two species. In contrast, the organization of these three genes is relatively conserved in *R. sacra* and *R. tangutica*, which may reflect functional constraints during evolution. Additionally, *nad5* is absent from *R. juparensis*, whereas *nad1*, *nad2*, and *nad5* are all missing from *R. kirilowii*, whose chromosomes are also conspicuously shorter. These observations indicate a substantial likelihood of remaining assembly errors in the mitogenomes of both species.

**Figure 3:**
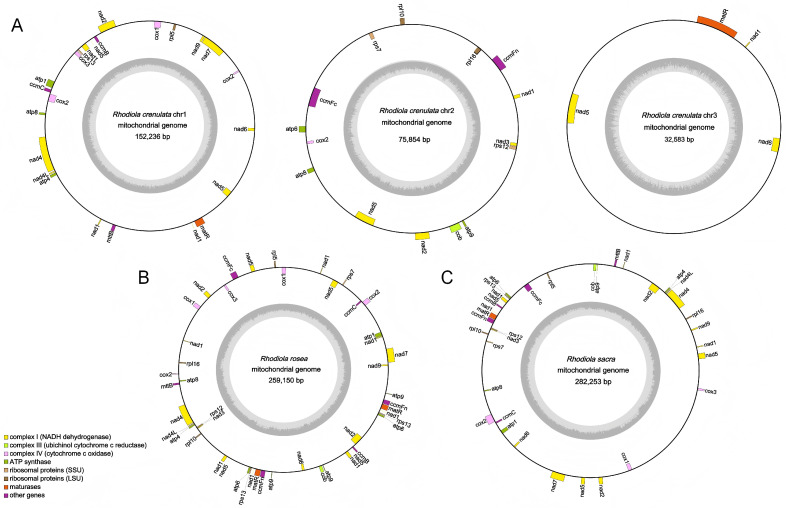
Mitogenome maps of *R. crenulata* (**A**), *R. rosea* (**B**), and *R. sacra* (**C**). Genes shown on the inner and outer circles are transcribed clockwise and counterclockwise, respectively. Different colors indicate functional gene groups.

In this study, the mitogenomes of *R. crenulata*, *R. rosea*, and *R. sacra*, three important medicinal species of *Rhodiola*, were assembled (Figure 3A–C). In contrast to previous reports, our assembly revealed that *R. crenulata* possesses three circular mitochondrial chromosomes, whereas *R. sacra* exhibit a single circular chromosome. This differs from earlier studies, which described *R. crenulata* as having a single circular and *R. sacra* as consisting of two circles. The newly assembled mitogenomes of both *R. crenulata* and *R. sacra* contain the full set of 30 PCGs, consistent with the results obtained from our earlier Polypods-based assembly using second-generation transcriptomic data. The two additional circular chromosomes (Chr2 and Chr3) identified in *R. crenulata*, previously missing in published assemblies, harbor several PCGs that were omitted in earlier annotations. Specifically, Chr2 contains *atp9*, *ccmFn*, *cob*, *nad3*, *rpl16*, *rps7*, and *rps12*, while Chr3 carries exon segments of *nad1* and *nad5*. Furthermore, the *cox2* gene, which was not annotated in earlier *R. crenulata* assemblies, was identified on the newly assembled Chr1 using the RhoMitoAnnotator. For *R. sacra*, the single circular chromosome assembled in this study incorporates all PCGs reported in the earlier two-circle model, along with additional genes that were previously overlooked. Through systematic sequencing, assembly, and annotation, this study provides a substantially improved representation of these mitogenomes, recovering genes and structural elements that were absent in prior assemblies.

### 2.4. Implementation of the Polypods Pipeline

**Figure 4:**
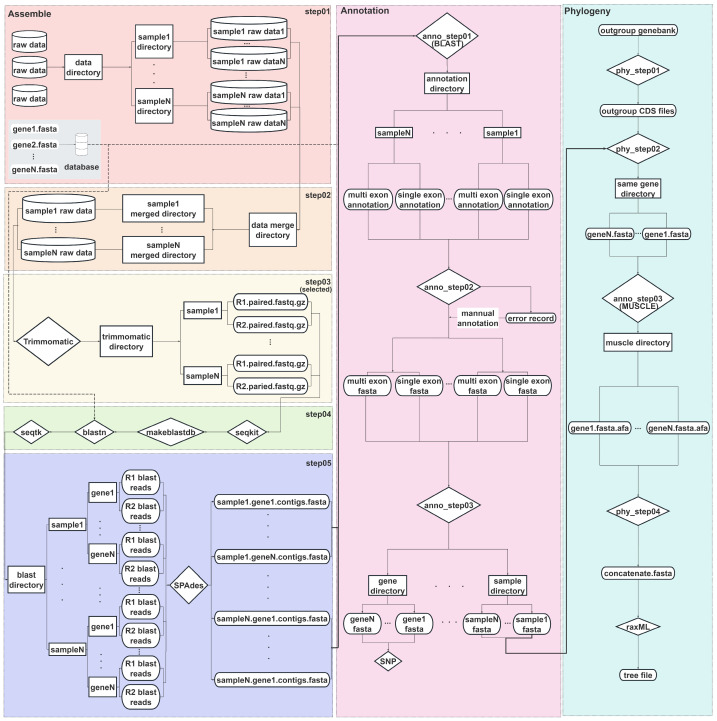
Overview of the Polypods workflow, which integrates three main pipelines for mitochondrial gene analysis: assembly, annotation, and phylogenetic reconstruction.

Given the core requirement of utilizing only PCGs in most population genomics studies, we developed a novel bioinformatics pipeline, Polypods (Figure 4). This pipeline utilizes low-cost Illumina short-read sequencing data along with curated reference sequences to assemble complete CDSs and achieves accurate annotation through an integrated annotation workflow. In this study, Polypods was validated using *Rhodiola* datasets and demonstrated its utility for reference-guided mitochondrial PCG recovery in this genus. Its modular design may allow adaptation to other plant groups, but such applications will require lineage-specific reference databases and parameter recalibration.

#### 2.4.1. Assembly Pipeline

The assembly pipeline of Polypods comprises five sequential scripts (Step 01–05). This pipeline accepts either raw sequencing data (.fastq.gz/.fq.gz) or preprocessed, quality-controlled paired-end reads (paired.fastq.gz/paired.fq.gz) as input, and proceeds as follows: (1) If raw sequencing data are provided, Step 01 first organizes files into sample-specific folders based on filename prefixes. When multiple sequencing runs exist for the same sample, Step 02 merges the corresponding files. Subsequently, Step 03 performs adapter trimming and quality filtering using Trimmomatic. (2) If input data have already been quality-controlled, users may skip Trimmomatic by executing only Step 01 and Step 02. After sample data are organized, the pipeline proceeds directly to Step 04, omitting the quality control step (Step 03).

Prior to running Step 04 for non-*Rhodiola* species, a database folder must be prepared containing reference gene sequences for assembly. In this step, we provide the *Rhodiola* PCGs database as a case study. The pipeline allows users to assemble either all genes in the database or a selected subset of interest. It is noteworthy that in *Rhodiola*, the nad5 gene contains a minimal exon of only 22 bp, which cannot be reliably captured by standard BLAST searches. To accurately identify this small exon, we adopted a strategy inspired by the ISECUS algorithm [17], extracting 150 bp of conserved sequence from both its upstream and downstream regions and appending these to the *nad5* reference file (Supplementary Figure S3). Following gene selection, Step 04 converts the processed paired-end FASTQ files into FASTA format using seqkit [18], and constructs BLAST databases for each FASTA file with makeblastdb. The reference sequences from the database are then aligned against the reads using BLASTn. To maximize assembly coverage and avoid unpaired reads, a union set of read names mapped to the reference is compiled, and the corresponding paired-end reads are extracted into separate FASTQ files using seqtk. Finally, Step 05 performs de novo assembly of the extracted reads with SPAdes (Supplementary Table S8).

#### 2.4.2. Annotation Pipeline

The contigs assembled by SPAdes do not represent complete coding sequences (CDSs) and therefore cannot be used directly for downstream analysis. They must be annotated and assembled into full-length genes based on reference sequences. Compared to the assembly pipeline, the annotation pipeline is relatively straightforward, consisting of three scripts that primarily rely on BLAST. In anno_step 01, the pipeline first uses makeblastdb to create a BLAST database from the assembled contigs. It then aligns reference sequences from the assembled database to this contig database. For single-exon genes, the optimal alignment is selected by evaluating alignment length. For multi-exon genes, the corresponding alignments are chosen based on the length of each exon to reconstruct the gene structure. In anno_step 02, the pipeline assembles the CDSs separately for single-exon and multi-exon genes according to the annotation results and generates an error log. This log highlights potential issues that may have occurred during the annotation process, enabling users to review the corresponding BLAST outputs and perform manual curation when necessary. A common source of annotation errors arises when sequences from one exon are fragmented and assembled into separate contigs. This leads to two distinct alignment records for the same exon during the BLAST step, which the annotation pipeline cannot resolve automatically. In anno_step 03, the pipeline organizes the final annotation results into two separate directories. One directory contains gene-specific files, each consisting of the FASTA sequences of a given gene across all samples. The other contains sample-specific files, each comprising all PCG sequences for the respective sample. The gene-based files are suitable for SNP analysis, whereas the sample-based files are optimized for downstream phylogenetic tree reconstruction.

#### 2.4.3. Phylogenetic Pipeline

As a complete analytical workflow, Polypods provides a pipeline for constructing maximum likelihood (ML) trees using a concatenation approach. However, unlike the assembly and annotation pipelines, this step is not mandatory; users may opt to use it based on their specific needs. The pipeline consists of four steps. First, users must select an outgroup species and download the corresponding mitogenome file in GenBank format containing CDS information. Phy_step 01 of the pipeline extracts the CDSs of the outgroup species based on the annotations in the GenBank file. If CDS files of outgroup species already existed, users can proceed directly to phy_step 02.

Next, phy_step 02 identifies genes shared by the outgroup CDS dataset and the assembled sample CDS dataset according to gene names. phy_step 03 then performs multiple sequence alignment using MUSCLE. Finally, phy_step 04 concatenates the aligned sequences into a single matrix for phylogenetic reconstruction and submits the file to RAxML to construct a maximum likelihood (ML) tree for the assembled samples. During this process, missing genes or incomplete CDS regions in individual samples, which may result from low sequencing depth, poor sample quality, or incomplete assembly, can lead to unequal sequence lengths and cause errors during tree construction. In such cases, a cautious temporary treatment is to fill the missing region using the corresponding aligned sequence from a conspecific sample, but only when the missing fragment is located in a region that is completely conserved within the genus and when the aim is to reduce technical artifacts caused by incomplete assembly rather than to infer novel sequence variation. If a sample contains a large number of missing genes or extensive incomplete regions, we recommend excluding that sample from the phylogenetic analysis, which represents a better compromise between successful tree construction and analytical reliability. In addition, the current pipeline does not use IQ-TREE or other tools to automatically select the optimal evolutionary model. Users can either specify a model recognized by RAxML or use the default model, “GTR+GAMMA+I”.

### 2.5. Benchmarking of Polypods Assembly and RhoMitoAnnotator Annotation Performance

**Figure 5:**
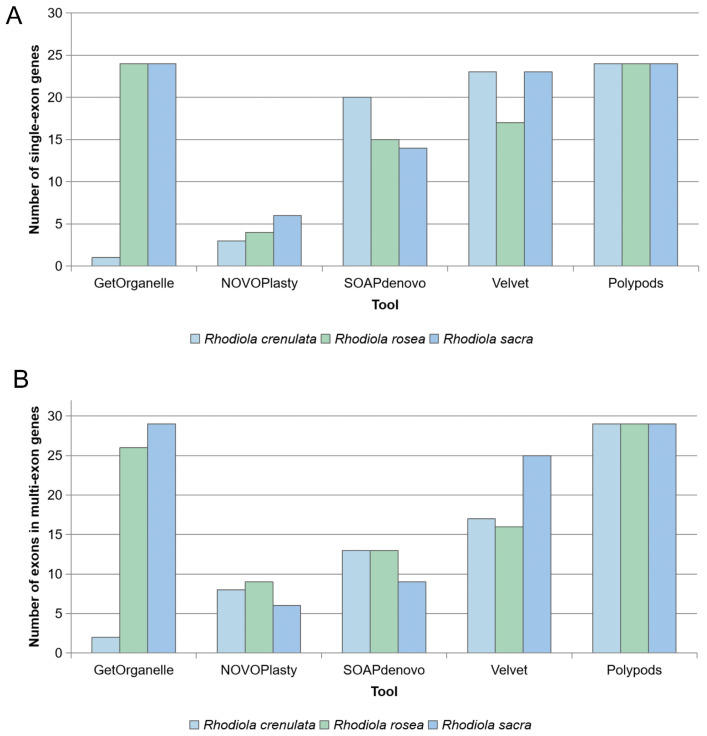
Comparison of mitochondrial PCG recovery among different assembly tools. (**A**) Number of assembled single-exon mitochondrial genes recovered from three representative *Rhodiola* samples. (**B**) Number of assembled exon fragments from multi-exon mitochondrial genes recovered from the same samples. Bars represent different *Rhodiola* species.

For the assembly-level comparison, Polypods was compared with four commonly used assemblers, including GetOrganelle, NOVOPlasty, SOAPdenovo, and Velvet (Figure 5). GetOrganelle and NOVOPlasty were included as organelle-targeted assemblers, whereas SOAPdenovo and Velvet were included as widely used general-purpose assemblers to provide a broader methodological comparison. Three samples, *R. crenulata* XZ_1, *R. rosea* HB_1, and *R. sacra* XZ_1, were selected for testing. All tools were run using their default parameters. For assembly tools requiring a reference genome, the previously assembled mitochondrial genome of *R. rosea* (NCBI accession number: PP024540.1) was used as the reference. Because these tools generated different types of assembly outputs, we evaluated the reliability of mitochondrial PCG recovery using the number of annotated single-exon genes and the number of exon fragments identified from multi-exon genes. The statistical results showed that, among the four established assembly tools, Velvet recovered the largest number of genes and exons. GetOrganelle produced relatively complete assemblies for the *R. rosea* HB_1 and *R. sacra* XZ_1 samples, but performed poorly for the *R. crenulata* XZ_1 sample. SOAPdenovo also recovered a relatively large number of genes and exons, whereas NOVOPlasty showed the poorest assembly performance among these tools. In the three tested samples, Polypods recovered all single-exon genes and all detectable exon fragments of multi-exon genes using the curated *Rhodiola* reference database. These results suggest that Polypods is effective for reference-guided mitochondrial PCG recovery in *Rhodiola*. By enabling the complete recovery of mitochondrial PCGs and exon fragments, Polypods provides reliable genomic materials for phylogenetic and other downstream analyses.

To further assess the annotation performance of RhoMitoAnnotator, we compared its results with those generated by PMGA, a recently developed plant mitogenome annotation tool, using seven re-annotated *Rhodiola* mitogenomes. Because no independent manually curated reference annotations were available for all seven genomes, we focused on tool-to-tool concordance and manually inspected major discrepant loci rather than directly claiming absolute annotation accuracy. The comparison included gene recovery, CDS/exon boundary concordance, translated protein concordance, RNA-editing-associated start/stop codon representation, and pseudogene annotation. PMGA tended to annotate a larger number of CDS features and additionally reported tRNA and rRNA features, whereas the RhoMitoAnnotator produced a more conservative PCG-focused annotation. Across the seven *Rhodiola* mitogenomes, PMGA and the RhoMitoAnnotator shared 141 protein-coding gene annotations (Supplementary Table S9). Among these shared genes, 110/141 showed identical CDS/exon boundaries, 103/141 produced identical translated protein sequences after excluding terminal stop symbols, and 96/141 matched in both coordinates and protein sequence. To compare the annotation differences between the two tools in greater detail, we compiled the tool-specific CDS annotations, as well as the genes with discrepancies in boundaries or protein sequences, from PMGA and RhoMitoAnnotator (Supplementary Table S10). The PMGA-specific CDS annotations included genes such as *rps4*, *sdh3*, *sdh4*, and *rps14*. When these genes were processed by RhoMitoAnnotator with the curated reference database, they were annotated as pseudogenes. In addition, boundary inconsistencies were observed for a set of genes, including *mttB*, *nad6*, *nad7*, *rpl16*, *cob*, *matR*, and *ccmFn*. These discrepancies mainly arose from differences in start-site selection, exon-boundary placement, and the handling of RNA-editing-associated start/stop codons. In the RhoMitoAnnotator, a gene is labeled as a pseudogene when its exon number differs from that recorded in the curated reference database, with an additional exon-order validation step available to detect abnormal exon arrangements. Accordingly, several potentially disrupted genes, particularly *nad1*, *nad2*, and *nad5*, were annotated as pseudogenes by the RhoMitoAnnotator rather than as intact CDSs. The RhoMitoAnnotator also provided more structured annotation of RNA-editing-associated start and stop codons in several mitochondrial PCGs, such as *atp6*, *atp8*, *nad1*, and *nad4L*. PMGA and the RhoMitoAnnotator showed comparable runtime performance, with both tools generally completing each *Rhodiola* mitogenome annotation within approximately 5 min under our computing environment. Overall, PMGA provides broader general mitogenome feature annotation, including RNA genes, whereas the RhoMitoAnnotator offers conservative PCG-oriented annotation with explicit treatment of pseudogenes and RNA-editing-associated coding boundaries.

### 2.6. Variation Analysis of Mitochondrial PCGs in Rhodiola Based on Population Data

Utilizing Illumina sequencing data from 108 *Rhodiola* samples, this study successfully assembled and annotated the mitochondrial PCGs using the Polypods pipeline, enabling a systematic evaluation of the genetic variation landscape within the genus at the population level. SNP detection across PCGs revealed variation characteristics of different functional genes and their potential evolutionary implications. Analysis results showed that genes such as *matR* (32 SNPs), *atp8* (24 SNPs), and *atp1* (23 SNPs) exhibited relatively high genetic diversity, suggesting that these energy metabolism-related genes may have undergone positive adaptive differentiation during the evolution of *Rhodiola*. In contrast, genes including *nad3* (2 SNPs), *nad4L* (2 SNPs), *rpl10* (1 SNP), and *atp9* (0 SNPs) showed low SNP counts, indicating a high degree of sequence conservation (Supplementary Table S11).

Several mitochondrial genes displayed unique structural variation patterns within the genus *Rhodiola*, showing varying degrees of species specificity. The initial 619 bp region of the *nad6* gene was relatively conserved, with only nine SNP sites identified, whereas the sequence following this region showed significantly reduced conservation across species, suggesting that the 3′ end of this gene may have experienced a lineage-specific evolutionary process. Variations in *nad9* and *ccmC* exhibited clear species specificity: variation in the 5′ region of *nad9* was detected only in *R. crenulata*, while the 5′ end variation in *ccmC* was unique to *R. rosea*, consistent with our PCR validation results. However, the 5′ end variation in the *ccmFn* gene was not exclusive to *R. crenulata*; similar variation patterns were also detected in multiple species including *R. wallichiana* var. *cholaensis*, *R. tangutica*, *R. sachalinensis*, and *R. yunnanensis*, implying that this variation may be present within a specific evolutionary clade. Notably, internal stop codons were commonly identified in *rpl16* across the examined *Rhodiola* species. In *R. crenulata*, *R. dumulosa*, *R. quadrifida*_XZ_3, and *R. sacra*_YN, a G→T substitution at *rpl16-73* directly created a TAA stop codon. Additional termination mutations were observed at distinct sites in other samples: *rpl16-46* in the *R. atuntsuensis*_XZ_1, *rpl16-52* in *R. fastigiata*_XJ_2, and *rpl16-70* in *R. litwinowii*. In these accessions, the GTG codon at *rpl16-82* may serve as an alternative start codon. The occurrence of internal stop codons suggests that *rpl16* is likely undergoing pseudogenization in this genus. Furthermore, the coding sequence of the *ccmB* gene was highly conserved in the vast majority of samples. However, only *R. forrestii* collected from Yunnan exhibited a mutation from GAA to TTT at positions 19–21 bp, resulting in a non-synonymous amino acid substitution, reflecting a significant signal of lineage differentiation. Notably, the *nad1* gene contained an insertion of three amino acids (Ser-Lys-Lys) in species including *R. crenulata*, *R. sacra*, *R. rosea*, *R. sachalinensis*, *R. forrestii*, and *R. primuloides*. Notably, the stop codon of the *nad9* gene in *R. primuloides* was mutated from TAA to AAA, leading to an extension of its ORF and potentially resulting in a protein product with an altered functional structure. These species- or clade-specific variation sites not only reveal the dynamic evolutionary history of the mitogenome in the genus *Rhodiola* but also provide reliable genetic markers for molecular identification of species within the genus.

### 2.7. Phylogenetic Analysis Based on Rhodiola Mitochondrial and Chloroplast PCGs

**Figure 6:**
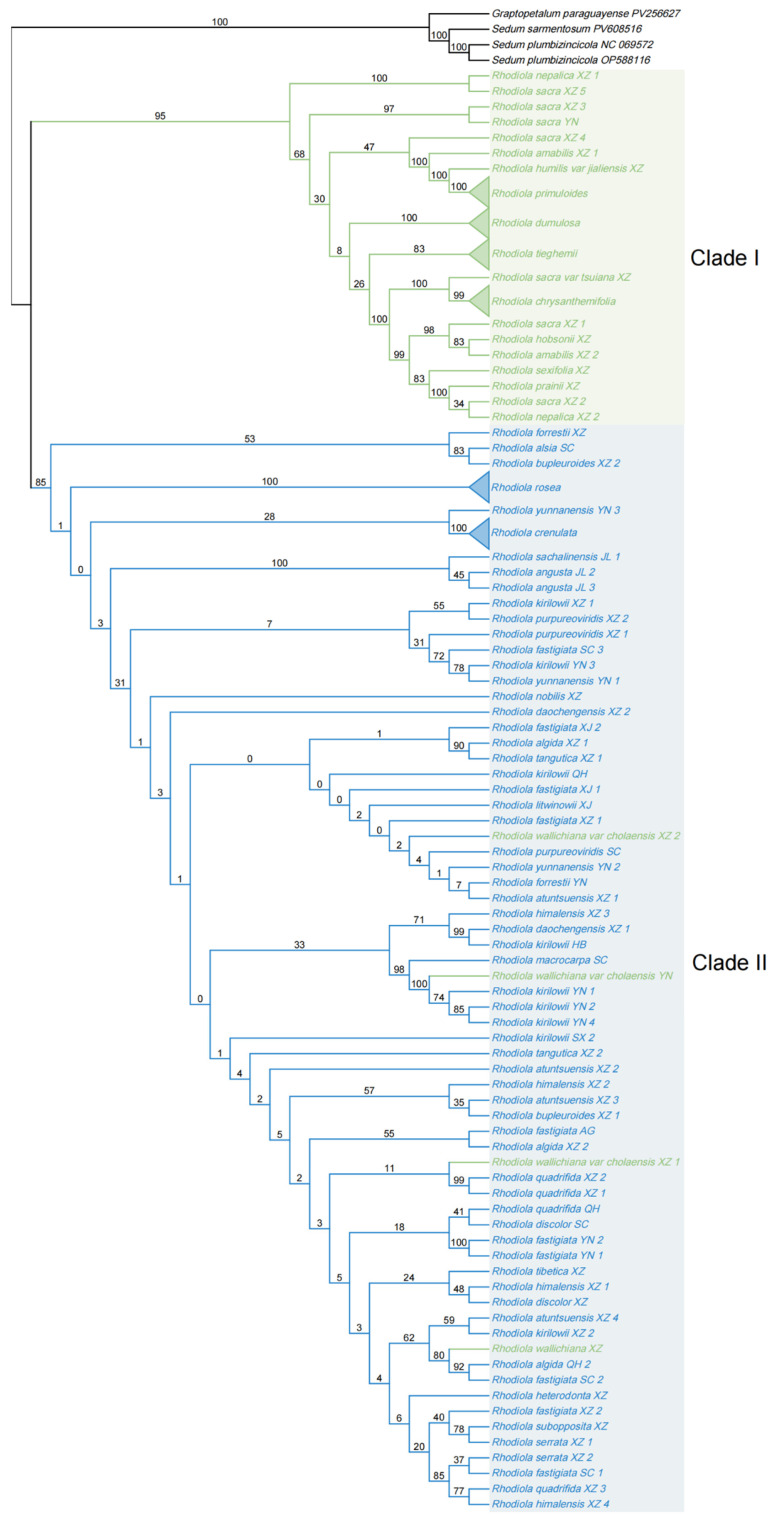
The ML tree of *Rhodiola* species inferred from concatenated mitochondrial CDSs. Four highly conserved mitochondrial genes (*atp9*, *rpl10*, *nad3*, and *nad4L*) are excluded to improve phylogenetic resolution. Bootstrap values from 1000 replicates are shown on the branches. The two shaded regions indicate the two major *Rhodiola* lineages, Clade I and Clade II. Green and blue colors represent hermaphroditic and dioecious taxa. Triangles indicate collapsed monophyletic species; species names are labeled next to the triangles.

**Figure 7:**
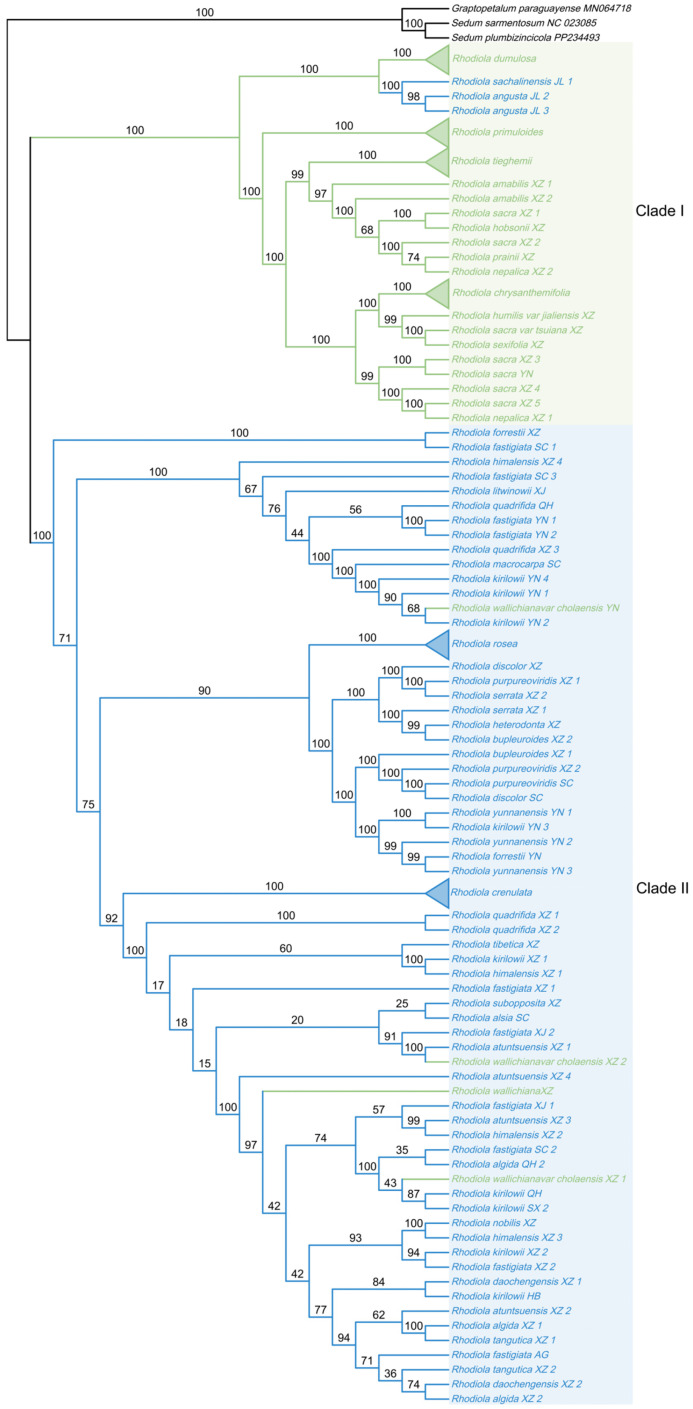
The ML tree of *Rhodiola* species inferred from concatenated chloroplast CDSs. Bootstrap values from 1000 replicates are shown on the branches. The two shaded regions indicate the two major *Rhodiola* lineages, Clade I and Clade II. Green and blue colors represent hermaphroditic and dioecious taxa. Triangles indicate collapsed monophyletic species; species names are labeled next to the triangles.

Phylogenetic trees were reconstructed using concatenated mitochondrial and chloroplast CDSs of *Rhodiola* species, with *Graptopetalum paraguayense*, *Sedum plumbizincicola*, and *S. sarmentosum* (Crassulaceae) designated as outgroups (Figure 6 and Figure 7). In both organellar phylogenies, *Rhodiola* species were broadly separated into two major lineages, here referred to as Clade I and Clade II. Clade I was mainly composed of hermaphroditic species, whereas Clade II was predominantly composed of dioecious species. This pattern suggests that cytoplasmic PCG datasets contain phylogenetic signals associated with sexual-system differentiation in *Rhodiola*, although these signals should be interpreted as lineage-associated rather than as direct evidence of sex determination.

The mitochondrial and chloroplast phylogenies showed broadly similar separation between the hermaphroditic-enriched and dioecious-enriched lineages, but they differed in branch support and the placement of several taxa. In the mitochondrial tree, Clade I was recovered as a basal lineage with relatively high support, while internal relationships within Clade II showed generally low bootstrap values, indicating limited phylogenetic resolution of mitochondrial PCGs for some dioecious lineages. In contrast, the chloroplast tree provided higher support for several internal branches and showed clearer resolution within parts of Clade II. These differences indicate that mitochondrial and chloroplast genomes provide complementary but not identical cytoplasmic phylogenetic signals.

Several representative species groups were consistently recovered in both analyses. For example, *R. crenulata*, *R. rosea*, *R. dumulosa*, *R. primuloides*, *R. chrysanthemifolia* and *R. tieghemii* each formed distinct and highly supported monophyletic groups in both mitochondrial and chloroplast phylogenies, supporting their genetic coherence at the organellar genome level. However, some taxa showed inconsistent placement between the two trees, and several species did not strictly follow the dominant sexual-system pattern of their corresponding clades. For instance, *R. sachalinensis* and *R. angusta* from Jilin populations were grouped within or close to the hermaphroditic-enriched lineage in the chloroplast tree, whereas they were placed in the dioecious-enriched lineage in the mitochondrial tree. These incongruences may reflect differences in organellar inheritance, lineage sorting, geographic differentiation, or historical introgression.

## 3. Discussion

In summary, this study successfully addressed the two major challenges of accurate annotation and large-scale acquisition of plant mitochondrial PCGs by developing the high-precision annotation tool RhoMitoAnnotator and the high-throughput analytical pipeline Polypods. Applying this methodological framework, we completed mitochondrial genome maps for three representative species, re-annotated seven publicly available mitogenomes, and conducted population-scale organellar PCGs analysis for 108 samples from 39 species. This work comprehensively characterized the diversity, structural variation, and evolutionary dynamics of the mitogenomes in *Rhodiola*. Furthermore, a combined phylogenetic analysis based on mitochondrial and chloroplast PCGs revealed that dioecious and hermaphroditic lineages form strongly supported, distinct clades. This result supported the hypothesis of an evolutionary transition from hermaphroditism to dioecy in *Rhodiola* and indicated that the sexual system is a stable phylogenetic trait within the genus. Consequently, our study not only provided essential data and a methodological foundation for understanding the evolutionary mechanisms of organellar genomes in *Rhodiola*, but also provided a reference-guided framework for large-scale mitochondrial PCG analysis in *Rhodiola*, with potential transferability to other plant lineages after appropriate database construction and parameter optimization.

### 3.1. Complex Structure and Pervasive Misannotation of Plant Mitochondrial Genes

Systematic analyses reveal that mitochondrial gene entries in current public databases frequently contain assembly and annotation errors, including omissions in assembly or annotation, inaccuracies in gene boundary delineation, and misclassification of pseudogenes. These annotation inconsistencies not only compromise data reliability but also propagate erroneous conclusions in related research, while correcting such errors typically requires substantial manual curation [17,19].

During the curation of mitochondrial PCGs in *Rhodiola*, we observed numerous instances of gene boundary variation. In this study, we identified and verified authentic 5′ end sequence variations in the *nad9* and *ccmFn* genes of *R. crenulata*, as well as in the *ccmC* gene of *R. rosea*. The 5′-terminal sequence of a mitochondrial gene not only influences transcriptional initiation efficiency but often encodes the N-terminal region of the protein, which can participate in mitochondrial targeting signaling or subunit assembly. Thus, variation in this region has clear functional implications. In *R. crenulata*, *nad9* encodes a core subunit of mitochondrial respiratory chain Complex I, and sequence variation in this gene may be relevant to mitochondrial energy metabolism. Previous studies on tobacco have shown that disruption of *nad9* impairs respiratory chain function and is associated with abnormal floral organ development, including petal deformation, anther degeneration, and male sterility [20]. In the present study, the *nad9* variant identified in *R. crenulata* was observed in male flowers characterized by carpel sterility. However, in the absence of functional validation, a causal relationship between this variant and carpel sterility cannot be established. Therefore, this *nad9* variation should be interpreted as a candidate mitochondrial marker potentially associated with reproductive organ differentiation in *R. crenulata*, rather than as direct evidence of a functional determinant of sex differentiation. Further studies incorporating gene expression profiling, mitochondrial respiratory activity assays, and genetic or transgenic validation will be necessary to determine whether this variant contributes directly to floral organ development or sexual-system differentiation.

Furthermore, our specialized approach to annotating the *nad6* gene highlights the challenges inherent in plant mitochondrial gene annotation. Unlike most conserved mitochondrial genes, the sequence of *nad6* beyond 619 bp exhibits high variability among *Rhodiola* species. To accurately define its coding region, we predicted ORFs for candidate sequences from each species using ORF Finder and selected the CDS encoding the longest possible protein product as the standard reference for that species, thereby maximizing the retention of potential protein sequence information. Previous research has identified the widespread occurrence of non-stop codon-containing *nad6* transcripts in angiosperms as an ancient evolutionary trait [21]. Their work demonstrated that the mature 3′ end of *nad6* transcripts is typically determined by a cis-acting element located upstream of the genomic stop codon. These elements are primarily classified into two types: the canonical t-element and stable stem-loop structures [22]. Consequently, the variations observed in the *nad6* gene within *Rhodiola* may represent a lineage-specific manifestation of this universal stop codon-independent maturation mechanism, potentially reflecting an important aspect of adaptive evolution in the mitogenome of this genus.

This study revealed that the *rpl16* gene in *Rhodiola* is likely a pseudogene undergoing a multi-layered degradation process driven by the combined effects of DNA mutations and RNA editing. Our analysis identified a high-frequency C-to-U RNA editing event at *rpl16-37* (CAG→UAG), which introduced a premature stop codon at the transcript level. Concurrently, DNA sequence comparisons revealed multiple, independently arising mutations at sites such as *rpl16-46*, *52*, *70*, and *73* across different species and populations, leading to further translational truncation at the genomic level. Similar RNA-editing-mediated premature termination events have been reported for mitochondrial *rpl16* transcripts in other angiosperms, including *Petunia*, *Arabidopsis*, and rapeseed, suggesting that *rpl16* pseudogenization may have occurred repeatedly or may be lineage-dependent in plant mitogenomes [23,24,25]. Furthermore, previous studies have provided no evidence supporting its translation. In *A. thaliana*, *rpl16* is annotated with the remark that an “internal stop codon may indicate *rpl16* is a pseudogene or requires an alternative start codon.” Therefore, these results suggested that *rpl16* could no longer encode a full-length, functional ribosomal protein, supporting its ongoing pseudogenization in the genus. Future work employing plant mitochondrial isolation coupled with proteomic analysis is needed to verify the expression of the *rpl16* protein and to elucidate the molecular regulatory mechanisms and evolutionary implications of these coordinated DNA and RNA-level changes [26].

### 3.2. Evolution of the Sexual System and Organelle Discordance in Rhodiola

Based on chloroplast and mitochondrial PCG data, our phylogenetic reconstructions consistently yielded strongly supported clades corresponding to hermaphroditic and dioecious lineages. These results suggest that sexual systems show lineage-associated phylogenetic signals within the genus. In both trees, hermaphroditic groups occupied basal positions, a topology that is consistent with the hypothesis of an evolutionary transition from hermaphroditism to dioecy in *Rhodiola* [11]. Nevertheless, certain topological inconsistencies were observed between the two phylogenies, which may primarily be attributed to incomplete lineage sorting (ILS) and interspecific gene flow or introgression [27]. Such discordance is particularly pronounced in lineages that have undergone rapid radiative evolution [28]. While prior studies have addressed phylogenetic conflicts between plastid and nuclear genomes in this genus, the phylogenetic relationships between chloroplast and mitogenomes have not been systematically compared.

Our analyses reveal that the chloroplast gene tree of *Rhodiola* exhibits a well-resolved topology with high nodal support and clearly defined species clustering. In contrast, the mitochondrial gene tree shows lower support values within several clades, and the topological stability of certain branches is notably weaker. This pattern of low support persists in the mitochondrial tree even after excluding four highly conserved genes (*atp9*, *rpl10*, *nad3*, and *nad4L*). We hypothesize that a key factor driving this discordance is the difference in evolutionary rates and selective pressures acting on the two organellar genomes. Chloroplast genes may accumulate genetic variation more readily, thereby providing a stronger phylogenetic signal for resolution. Mitochondrial genes experience stronger purifying selection, leading to a slower accumulation of substitutions, which limits their phylogenetic informativeness [29,30]. Notably, internal nodes within dioecious lineages generally show low support, suggesting that these groups may have experienced recent rapid radiation, leaving insufficient time for the mitogenome to accumulate lineage-specific substitutions. Additionally, in species such as *R. crenulata* and *R. rosea*, the phylogenetic placement differs between the chloroplast and mitochondrial trees. This discrepancy indicates that the chloroplast genome in these species has accumulated more informative genetic variation compared to the mitogenome. Moreover, when mitogenomes contain multiple copies or heteroplasmic sequences, assembly biases introduced during sequence selection may skew phylogenetic inference [31]. Future studies should integrate data from additional single-copy nuclear genes, plastid markers, and complete mitogenomes [32,33]. Such a synergistic approach will be essential for more accurately resolving the speciation mechanisms and deciphering the co-evolutionary patterns between nuclear and organellar genomes in *Rhodiola*.

### 3.3. Advantages, Challenges, and Future Perspectives of the RhoMitoAnnotator and Polypods

The RhoMitoAnnotator was developed to improve the accuracy of gene annotation in plant mitogenomes, particularly for protein-coding genes with complex structures or non-standard translational features [34]. The tool incorporates several specialized annotation modules. EBAnno is designed to resolve complex multi-exon structures and to identify independent transcripts of trans-spliced genes together with their corresponding coding regions. REAnno and NCAnno are used to annotate RNA-edited and non-canonical start/stop codons, respectively [35]. Through the integration of these modules, the RhoMitoAnnotator improved the completeness and accuracy of mitochondrial PCG annotation in Rhodiola, enabling the correction of previously omitted genes, inaccurate exon boundaries, and non-standard coding features in public annotations. These curated annotations also provided the basis for constructing the high-quality mitochondrial reference gene database used in this study. Importantly, the RhoMitoAnnotator performs full gene-level annotation and generates standardized outputs, including corrected CDS and protein sequences as well as GenBank-format annotation files. Although Polypods also includes an annotation-related step, its purpose and implementation differ from those of RhoMitoAnnotator. The RhoMitoAnnotator is intended for precise mitogenome annotation and reference database construction, whereas Polypods is designed primarily for batch homology-based retrieval, extraction, and assembly of mitochondrial PCGs from large numbers of Illumina short-read datasets. Therefore, Polypods does not independently perform comprehensive de novo gene annotation or generate complete GenBank annotation files. Instead, it relies on a curated and accurately annotated reference sequence database, such as that generated by the RhoMitoAnnotator, to identify and assemble target mitochondrial PCGs across multiple samples. In this sense, the two tools are functionally complementary: the RhoMitoAnnotator provides high-confidence reference annotations, while Polypods applies these curated references to multi-sample mitochondrial PCG recovery and downstream phylogenetic analysis.

Despite its utility, the current version of RhoMitoAnnotator has several limitations. First, its annotation functions are currently focused on PCGs and do not yet include dedicated modules for tRNA and rRNA annotation. Second, although the tool can resolve several types of complex gene structures, it does not yet provide a reliable automated strategy for detecting and distinguishing potential frameshift mutations within CDS regions or individual exons. Such frameshifts may reflect genuine biological variation, sequencing errors, or assembly artifacts, and therefore require careful discrimination [24]. Future development of RhoMitoAnnotator should focus on two main directions: (1) broadening annotation coverage by incorporating non-coding RNA prediction tools to enable comprehensive annotation of mitochondrial genomic elements and (2) enhancing analytical depth by developing modules capable of detecting and validating frameshift mutations. As genomic resources for medicinal plants continue to expand, integrating machine learning methods could further improve annotation accuracy [36].

Polypods was developed as a batch-processing pipeline for recovering mitochondrial PCGs from standard and cost-effective Illumina short-read sequencing data. The pipeline integrates sequence assembly, homology-based PCG retrieval, sequence concatenation, and phylogenetic reconstruction, thereby providing a standardized workflow for comparative mitogenomic analysis in medicinal plants. In this study, Polypods enabled the construction of a population-level mitochondrial PCG dataset for *Rhodiola*, demonstrating its applicability to large-scale sample processing when a reliable reference gene database is available. However, the application of Polypods also revealed several technical challenges related to assembly completeness. When different exons of the same gene are assembled into separate contigs, fully automated reconstruction remains difficult, and manual inspection may still be required. For example, the *nad5* gene, which exceeds 2000 bp in length, contained gaps of 3–18 bp in several samples, including *R. sacra*_XZ_1, *R. crenulata*_XZ_3, *R. rosea*_JL_2, and *R. forrestii*_XZ. This result indicates that reliable assembly of relatively long mitochondrial genes requires sequencing data of sufficient depth and quality. In addition, a 65 bp gap was detected in the *nad1* gene of one *R. crenulata* medicinal material sample, probably due to DNA degradation in processed herbal materials [37]. To address these limitations, future improvements could include the incorporation of limited long-read sequencing data for valuable or degraded samples and the development of homology-guided gap-filling algorithms specifically adapted to plant mitogenomes. Such improvements would increase the robustness of Polypods for analyzing low-quality or fragmented sequencing datasets.

Nevertheless, RhoMitoAnnotator and Polypods were developed and validated using *Rhodiola* mitogenomic datasets, and several key components, including the curated reference gene database and the intron-length thresholds used in EBAnno, were optimized according to the characteristics of this genus. Previous phylogenetic and phylogenomic studies have suggested that *Rhodiola* has experienced rapid diversification, organellar discordance, and reticulate evolutionary processes [38], indicating that it represents a relatively complex system for evaluating mitochondrial gene annotation and comparative organellar analysis. Therefore, the successful application of these tools in *Rhodiola* provides a useful methodological framework, but does not by itself demonstrate universal performance across all plant lineages. For application to other taxa, users should construct lineage-specific reference databases, recalibrate key parameters such as intron-length thresholds, and validate complex annotations using transcriptomic or experimental evidence where possible. Future work will extend the evaluation of RhoMitoAnnotator and Polypods to additional plant groups with different mitochondrial genome architectures, thereby further assessing their robustness, transferability, and limitations.

## 4. Materials and Methods

### 4.1. Plant Material Collection and DNA Extraction

A total of 108 samples of 39 *Rhodiola* species were collected across the Tibetan Plateau and adjacent areas, as well as from the Taibai Mountains, Changbai Mountains, Bashang Plateau, Helan Mountains, and other areas (Supplementary Table S12). Yulin Lin, Yaodong Qi, Xinlei Zhao and Jinxin Liu from the Institute of Medicinal Plant Development, Chinese Academy of Medical Sciences and Peking Union Medical College, along with Jun Zhang from Yunnan Nationalities University, conducted the authentication of these species based on morphological characteristics and DNA barcoding. The samples were deposited in the Herbarium of the Institute of Medicinal Plant Development, Chinese Academy of Medical Sciences, Beijing, China. High-quality genomic DNA was isolated from the fresh leaves using the CTAB method [39], and the DNA quality and concentration were tested by 1% agarose gel electrophoresis, NanoDrop One spectrophotometer (Thermo Fisher Scientific, Waltham, MA, USA) and Qubit 4.0 Fluorometer (Thermo Fisher Scientific, Waltham, MA, USA).

### 4.2. Sequencing Method

This study employed a tiered sequencing approach to comprehensively investigate the mitochondrial genomics of the *Rhodiola* genus, strategically integrating multiple sequencing technologies to optimize the balance between data quality and cost-effectiveness.

#### 4.2.1. Transcriptome Sequencing for Annotation Support

To facilitate accurate gene annotation, particularly for determining gene boundaries and exon–intron structures, we generated rRNA-depleted transcriptome sequencing data using both Oxford Nanopore Technology (ONT) and Illumina sequencing platforms. For *R. sacra*, ONT full-length transcriptome sequencing was performed. After ribosomal RNA depletion, the remaining RNA population was reverse-transcribed, ligated with sequencing adapters, amplified, and converted into sequencing libraries. The libraries were loaded onto R10.4.1 flow cells and sequenced on a PromethION sequencer for 48–72 h. In addition, strand-specific rRNA-depleted total RNA-seq libraries were prepared for *R. crenulata*, *R. rosea*, and *R. sacra* using the Illumina platform. Ribosomal RNA was depleted from total RNA before RNA fragmentation and cDNA synthesis, with dUTP incorporated to preserve strand specificity. The cDNA was then subjected to end repair, A-tailing, adapter ligation, PCR amplification, and size selection. Quality-verified libraries were sequenced on an Illumina NovaSeq 6000 platform (Illumina, Inc., San Diego, CA, USA) to generate 150 bp paired-end reads. These rRNA-depleted transcriptome datasets captured both coding and non-coding transcripts and were used in this study as transcript evidence to support gene model annotation.

#### 4.2.2. Reference Genome Sequencing of *R. rosea*, *R. crenulata*, and *R. sacra*

To establish a high-quality reference, we performed hybrid sequencing for *R. rosea*, *R. crenulata*, and *R. sacra* combining second-generation (Illumina short-read) and third-generation (Nanopore long-read) technologies. The DNA libraries were sequenced on Illumina NovaSeq 6000 platform and 150 bp paired-end reads were generated. For Nanopore sequencing, libraries were constructed using the SQK-LSK109 ligation kit according to the standard protocol. The purified library was loaded onto primed R9.4 Spot-On Flow Cells and sequenced on a PromethION sequencer during a 48-h run. Base calling was performed using Oxford Nanopore’s GUPPY software (v0.3.0).

#### 4.2.3. Population-Level Genome Sequencing

For comparative genomic and phylogenetic analyses across the genus, we conducted population-scale sequencing of 108 *Rhodiola* samples. High-quality DNA was fragmented to an average size of 350 bp for PCR-free library construction. Libraries were sequenced on the Illumina NovaSeq 6000 platform (Illumina, Inc., San Diego, CA, USA), generating approximately 6 Gb of raw data per sample. Raw reads were processed with Trimmomatic v0.39 [40] to remove adapter sequences and low-quality bases, retaining high-quality paired-end reads for downstream bioinformatic analyses.

### 4.3. Assembly and Annotation Method

For the mitogenome assembly and annotation of *R. rosea*, *R. crenulata*, and *R. sacra*, the long-read sequencing data were directly de novo assembled to construct a draft using the default parameters of the Flye software v2.9.6-b1802 [41], and graphical assembly results in GFA format were obtained. The GFA files were visualized using Bandage v0.8.1 [42]. The contigs that belong to *Rhodiola* mitogenome were extracted using BLASTn module v2.16.0+ [43] with PCGs of *A. thaliana* mitogenome in Bandage. Finally, we used minimap2 v2.30 [44] to compare the BGI or Illumina short-reads with obtained contigs and used bedtools v2.31.1 [45] to export the mitochondrial short-reads. Finally, we used the Unicycler v0.5.1 [46] for hybrid assembly to obtain the final mitogenome sequence with filtered long reads and short reads. *A. thaliana* (NC_037304) and *Liriodendron tulipifera* (NC_021152.1) were selected as reference genomes for protein-coding genes of mitogenome, and RhoMitoAnnotator was used to annotate the mitogenome. The mitogenome map was visualized using OGDRAW software v1.3.1 [47].

### 4.4. Chloroplast PCGs Assembly, Annotation and Phylogenetic Tree Construction

For chloroplast PCGs assembly and annotation of *Rhodiola*, raw reads were processed using Trimmomatic v0.39 [40], and then were used for the assembly of chloroplast PCGs with GetOrganelle v1.7.0 [48]. The chloroplast PCGs were annotated using our previously developed tool, CPGAVAS2 (http://47.96.249.172:16019/analyzer/home, accessed on 22 October 2025). Some samples failed to assemble chloroplast PCGs through GetOrganelle or NovoPlasty due to data quality issues. For such cases, we adopted the Polypods process developed in this study, which successfully obtained complete chloroplast PCGs. Phylogenetic analyses were then performed using RAxML v8.2.12 [49], and bootstrap analyses were performed with 1000 replicates. The results of the phylogenetic analysis were visualized using FigTree v1.4.5.

### 4.5. PCR Method for Verification of 5′ End Variants

Specific primers were designed targeting the 5′ end variant regions of the *nad9* and *ccmFn* genes in *R. crenulata* and the *ccmC* gene in *R. rosea*. The primers were designed within 100 bp upstream and downstream of each variant site using Primer Premier 5.0 software (Supplementary Table S13). PCR amplification was performed on a T100 Thermal Cycler (Bio-Rad Laboratories, Inc., Hercules, CA, USA) under the following conditions: initial denaturation at 94 °C for 5 min; 40 cycles of denaturation at 94 °C for 30 s, annealing at 56 °C for 30 s, and extension at 72 °C for 45 s; followed by a final extension at 72 °C for 10 min. PCR products were detected by 1% agarose gel electrophoresis. The sequencing was performed using the ABI3730XL sequencer (Applied Biosystems, Thermo Fisher Scientific, Waltham, MA, USA). Codoncode 6.0.1 (CodonCode Corporation, Centerville, MA, USA) was used for forward and reverse sequence proofreading and assembly, and low-quality sequences and primer regions were removed.

### 4.6. Development of the RhoMitoAnnotator Tool and Microsynteny Analysis

RhoMitoAnnotator operates in the same computational environment as Polypods but relies solely on BLAST as its third-party tool. To obtain reliable intron lengths for transcript splitting, we analyzed the intron sizes of multi-exon genes from the mitogenomes of seven published *Rhodiola* species available on NCBI (*R. rosea*_PP024540.1, *R. crenulata*_NC_070303.1, *R. sacra*_OP312070.1, OP312071.1, *R. tangutica*_NC_072122.1, *R. wallichiana*_OP312068.1, OP312069.1, *R. kirilowii*_CM082116.1, *R. juparensis*_NC_082108.1). Based on this analysis, gene-specific intron length thresholds were established for transcript splitting. Initial annotation was performed using a reference sequence database. During the annotation process, we observed that certain gene sequences from some species exhibited considerable divergence in start and stop codon positions compared to those in the original reference database, leading to alignment and annotation anomalies. To address this, we manually extracted these atypical gene sequences and incorporated them into the reference database, thereby constructing a customized reference sequence library for the annotation pipeline.

Microsynteny blocks between *Rhodiola* species (curated genomes of *R. rosea*, *R. crenulata*, *R. sacra*, *R. tangutica*, *R. wallichiana*, *R. kirilowii*, *R. juparensis*) were identified using BLAST with default parameters. Then, JCVI was used to visualize the collinearity regions.

### 4.7. Pipelines of Polypods Software

The Polypods pipeline comprises six modular components: (1) DataPreparation (Step 01–02): Organize raw sequencing data by sample identity, merge datasets from the same sample, and perform standardized renaming; (2) QualityControl (Step 03): Process reads using Trimmomatic v0.39 [40] to remove adapters and filter out low-quality or unpaired sequences; (3) ExtractReads (Step 04): Construct a BLAST database using makeblastdb from quality-controlled reads, align reference sequences against this database via BLASTn, and extract relevant reads using seqtk v1.4 to generate sequence-specific FASTQ files; (4) GeneAssembly (Step 05): Perform de novo assembly of extracted reads using SPAdes v4.2.0 [50]; (5) GeneAnnotation (anno_step): Annotate assembled contigs and reconstruct complete CDSs using a GeneAnnotation module with a CPGAVAS2-based annotation engine; (6) Phylogeny (phy_step): the multiple sequence alignment was performed using MUSCLE. Merge annotated genes into an integrated mitochondrial sequence using Biopython. Phylogenetic analyses were then performed using RAxML v8.2.12 [49], and bootstrap analyses were performed with 1000 replicates.

## Data Availability

The source code, documentation, example commands, test datasets, and curated *Rhodiola* mitochondrial reference database for RhoMitoAnnotator (v1.0.0) and Polypods (v1.0.0) are available at GitHub (https://github.com/YiYiYiDa/RhoMitoAnnotator-and-Polypods.git, accessed on 3 May 2026). The complete mitochondrial genome sequences of *R. crenulata* and *R. sacra* have been submitted to NCBI under temporary submission IDs SUB16147978 and SUB16147912, respectively. Final accession numbers will be provided once the NCBI review process is completed. The *R. rosea* mitogenome used in this study is available under accession number PP024540.1.

## Supplementary Materials

The following supporting information can be downloaded at: https://www.mdpi.com/article/10.3390/ijms27104440/s1.

## References

[1] Gualberto J.M., Newton K.J. (2017). Plant Mitochondrial Genomes: Dynamics and Mechanisms of Mutation. Annu. Rev. Plant Biol..

[2] Møller I.M., Rasmusson A.G., Van Aken O. (2021). Plant mitochondria-past, present and future. Plant J..

[3] Morley S.A., Nielsen B.L. (2017). Plant mitochondrial DNA. Front. Biosci..

[4] Small I.D., Schallenberg-Rüdinger M., Takenaka M., Mireau H., Ostersetzer-Biran O. (2020). Plant organellar RNA editing: What 30 years of research has revealed. Plant J..

[5] Wang J., Kan S., Liao X., Zhou J., Tembrock L.R., Daniell H., Jin S., Wu Z. (2024). Plant organellar genomes: Much done, much more to do. Trends Plant Sci..

[6] Bi C., Shen F., Han F., Qu Y., Hou J., Xu K., Xu L.-a., He W., Wu Z., Yin T. (2024). PMAT: An efficient plant mitogenome assembly toolkit using low-coverage HiFi sequencing data. Hortic. Res..

[7] Milián-García Y., Hempel C.A., Janke L.A.A., Young R.G., Furukawa-Stoffer T., Ambagala A., Steinke D., Hanner R.H. (2022). Mitochondrial genome sequencing, mapping, and assembly benchmarking for *Culicoides* species (Diptera: Ceratopogonidae). BMC Genom..

[8] Liu X.Y., Zhang D.Q., Zhang J.Q. (2023). Plastomic data shed new light on the phylogeny, biogeography, and character evolution of the family Crassulaceae. J. Syst. Evol..

[9] Tao H., Wu X., Cao J., Peng Y., Wang A., Pei J., Xiao J., Wang S., Wang Y. (2019). *Rhodiola* species: A comprehensive review of traditional use, phytochemistry, pharmacology, toxicity, and clinical study. Med. Res. Rev..

[10] Huang L., Yang Y.P., Liu X.Y., Qiu L.F., Li Y.Y., Ma Z.W., Wang S.Y., Wang X.Y., Zhang J.Q. (2025). Integrated analyses reveal extensive cytonuclear discordance and two new members of *Rhodiola*. J. Syst. Evol..

[11] Liu J., Zang E., Tian Y., Zhang L., Li Y., Shi L., Xu L., Xiao P. (2024). Comparative chloroplast genomes: Insights into the identification and phylogeny of rapid radiation genus *Rhodiola*. Front. Plant Sci..

[12] Garewal N., Goyal N., Pathania S., Kaur J., Singh K. (2021). Gauging the trends of pseudogenes in plants. Crit. Rev. Biotechnol..

[13] Grohmann L., Thieck O., Herz U., Schröder W., Brennicke A. (1994). Translation of nad9 mRNAs in mitochondria from Solanum tuberosum is restricted to completely edited transcripts. Nucleic Acids Res..

[14] Morawala-Patell V., Gualberto J.M., Lamattina L., Grienenberger J.M., Bonnard G. (1998). Cis-and trans-splicing and RNA editing are required for the expression of nad2 in wheat mitochondria. Mol. Gen. Genet..

[15] Nugent J.M., Palmer J.D. (1993). Characterization of the Brassica campestris mitochondrial gene for subunit six of NADH dehydrogenase: Nad6 is present in the mitochondrion of a wide range of flowering plants. Curr. Genet..

[16] Lenz H., Hein A., Knoop V. (2018). Plant organelle RNA editing and its specificity factors: Enhancements of analyses and new database features in PREPACT 3.0. BMC Bioinform..

[17] Shi L., Chen H., Jiang M., Wang L., Wu X., Huang L., Liu C. (2019). CPGAVAS2, an integrated plastome sequence annotator and analyzer. Nucleic Acids Res..

[18] Shen W., Sipos B., Zhao L. (2024). SeqKit2: A Swiss army knife for sequence and alignment processing. Imeta.

[19] Lang B.F., Beck N., Prince S., Sarrasin M., Rioux P., Burger G. (2023). Mitochondrial genome annotation with MFannot: A critical analysis of gene identification and gene model prediction. Front. Plant Sci..

[20] Forner J., Kleinschmidt D., Meyer E.H., Gremmels J., Morbitzer R., Lahaye T., Schöttler M.A., Bock R. (2023). Targeted knockout of a conserved plant mitochondrial gene by genome editing. Nat. Plants.

[21] Yu R., Liu L., Jost M., Zhao R., Wanke S., Jiao Y. (2025). Evolution of mitochondrial RNA editing sites and stop codon-lacking transcripts in angiosperms. Commun. Biol..

[22] Forner J., Weber B., Thuss S., Wildum S., Binder S. (2007). Mapping of mitochondrial mRNA termini in Arabidopsis thaliana: T-elements contribute to 5′ and 3′ end formation. Nucleic Acids Res..

[23] Sutton C.A., Conklin P.L., Pruitt K.D., Calfee A.J., Cobb A.G., Hanson M.R. (1993). Editing of rps3/rpl16 transcripts creates a premature truncation of the rpl16 open reading frame. Curr. Genet..

[24] Varré J.-S., D’Agostino N., Touzet P., Gallina S., Tamburino R., Cantarella C., Ubrig E., Cardi T., Drouard L., Gualberto J.M. (2019). Complete Sequence, Multichromosomal Architecture and Transcriptome Analysis of the Solanum tuberosum Mitochondrial Genome. Int. J. Mol. Sci..

[25] Handa H. (2003). The complete nucleotide sequence and RNA editing content of the mitochondrial genome of rapeseed (*Brassica napus* L.): Comparative analysis of the mitochondrial genomes of rapeseed and Arabidopsis thaliana. Nucleic Acids Res..

[26] Ni Y., Li J., Tan Y., Shen G., Liu C. (2025). Advance in the assembly of the plant mitochondrial genomes using high-throughput DNA sequencing data of total cellular DNAs. Plant Biotechnol. J..

[27] You J., Lougheed S.C., Zhao Y., Zhang G., Liu W., Lu F., Wang Y., Zhang W., Yang J., Qiong L. (2022). Comparative phylogeography study reveals introgression and incomplete lineage sorting during rapid diversification of *Rhodiola*. Ann. Bot..

[28] Zhang J.Q., Meng S.Y., Allen G.A., Wen J., Rao G.Y. (2014). Rapid radiation and dispersal out of the Qinghai-Tibetan Plateau of an alpine plant lineage *Rhodiola* (Crassulaceae). Mol. Phylogenet. Evol..

[29] Palmer J.D., Adams K.L., Cho Y., Parkinson C.L., Qiu Y.L., Song K. (2000). Dynamic evolution of plant mitochondrial genomes: Mobile genes and introns and highly variable mutation rates. Proc. Natl. Acad. Sci. USA.

[30] Bentolila S., Stefanov S. (2012). A reevaluation of rice mitochondrial evolution based on the complete sequence of male-fertile and male-sterile mitochondrial genomes. Plant Physiol..

[31] Zwonitzer K.D., Tressel L.G., Wu Z., Kan S., Broz A.K., Mower J.P., Ruhlman T.A., Jansen R.K., Sloan D.B., Havird J.C. (2024). Genome copy number predicts extreme evolutionary rate variation in plant mitochondrial DNA. Proc. Natl. Acad. Sci. USA.

[32] Wang N., Li C., Kuang L., Wu X., Xie K., Zhu A., Xu Q., Larkin R.M., Zhou Y., Deng X. (2022). Pan-mitogenomics reveals the genetic basis of cytonuclear conflicts in citrus hybridization, domestication, and diversification. Proc. Natl. Acad. Sci. USA.

[33] Štorchová H., Krüger M. (2024). Methods for assembling complex mitochondrial genomes in land plants. J. Exp. Bot..

[34] Tillich M., Lehwark P., Pellizzer T., Ulbricht-Jones E.S., Fischer A., Bock R., Greiner S. (2017). GeSeq-versatile and accurate annotation of organelle genomes. Nucleic Acids Res..

[35] Castandet B., Choury D., Bégu D., Jordana X., Araya A. (2010). Intron RNA editing is essential for splicing in plant mitochondria. Nucleic Acids Res..

[36] Li J., Ni Y., Lu Q., Chen H., Liu C. (2025). PMGA: A plant mitochondrial genome annotator. Plant Commun..

[37] Liu J., Zang E., Tian Y., Li X., Xin T., Zeng L., Xu L., Xiao P. (2025). Applications and challenges of DNA barcoding in rapid radiation groups: *Rhodiola* (Crassulaceae) as a case study. Chin. Herb. Med..

[38] Ren C.Q., Zhang D.Q., Liu X.Y., Zhang J.Q. (2023). Genomic data provide a robust phylogeny backbone for *Rhodiola* L. (Crassulaceae) and reveal extensive reticulate evolution during its rapid radiation. Mol. Phylogenet. Evol..

[39] Arseneau J.R., Steeves R., Laflamme M. (2017). Modified low-salt CTAB extraction of high-quality DNA from contaminant-rich tissues. Mol. Ecol. Resour..

[40] Bolger A.M., Lohse M., Usadel B. (2014). Trimmomatic: A flexible trimmer for Illumina sequence data. Bioinformatics.

[41] Kolmogorov M., Yuan J., Lin Y., Pevzner P.A. (2019). Assembly of long, error-prone reads using repeat graphs. Nat. Biotechnol..

[42] Wick R.R., Schultz M.B., Zobel J., Holt K.E. (2015). Bandage: Interactive visualization of de novo genome assemblies. Bioinformatics.

[43] Camacho C., Coulouris G., Avagyan V., Ma N., Papadopoulos J., Bealer K., Madden T.L. (2009). BLAST+: Architecture and applications. BMC Bioinform..

[44] Li H. (2018). Minimap2: Pairwise alignment for nucleotide sequences. Bioinformatics.

[45] Quinlan A.R., Hall I.M. (2010). BEDTools: A flexible suite of utilities for comparing genomic features. Bioinformatics.

[46] Wick R.R., Judd L.M., Gorrie C.L., Holt K.E. (2017). Unicycler: Resolving bacterial genome assemblies from short and long sequencing reads. PLoS Comput. Biol..

[47] Greiner S., Lehwark P., Bock R. (2019). OrganellarGenomeDRAW (OGDRAW) version 1.3.1: Expanded toolkit for the graphical visualization of organellar genomes. Nucleic Acids Res..

[48] Jin J.J., Yu W.B., Yang J.B., Song Y., dePamphilis C.W., Yi T.S., Li D.Z. (2020). GetOrganelle: A fast and versatile toolkit for accurate de novo assembly of organelle genomes. Genome Biol..

[49] Stamatakis A. (2014). RAxML version 8: A tool for phylogenetic analysis and post-analysis of large phylogenies. Bioinformatics.

[50] Prjibelski A., Antipov D., Meleshko D., Lapidus A., Korobeynikov A. (2020). Using SPAdes De Novo Assembler. Curr. Protoc. Bioinform..

