# Supplementary material for: RhoMitoAnnotator and Polypods, Bioinformatics Tools for the Rhodiola Mitochondrial Gene Assembly, Annotation and Phylogenetic Analysis
Source: Int J Mol Sci. 2026 May 15;27(10):4440. doi: 10.3390/ijms27104440 (PMC13207870; doi:10.3390/ijms27104440)

Supplementary Figure S1. The exon boundary curation results of *Rhodiola crenulata* and *R. sacra*. The second-generation lncRNA data were mapped to the mitochondrial genome sequence to identify the exon boundaries, and the incorrect boundary information was annotated in the following subgraphs. The species name, accession number, gene name, and exon boundary number are provided in the upper-left corner. The originally annotated gene sequence is outlined in black, while the corrected sequence is highlighted in red, with the genomic positions of the first or last nucleotide indicated above the corresponding base. Supporting RNA-seq read alignments are displayed directly below the genomic sequence.

***Rhodiola crenulata*(NC_070303.1)** nad1 exon2 start

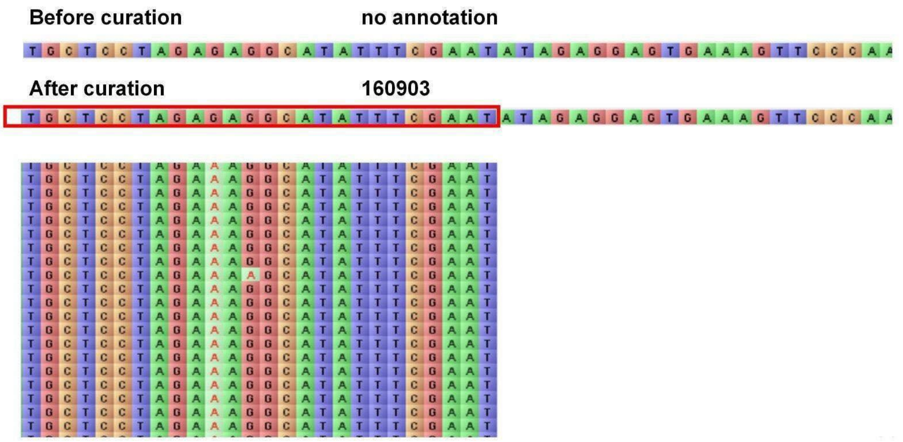

***Rhodiola crenulata*(NC_070303.1)** nad1 exon2 end

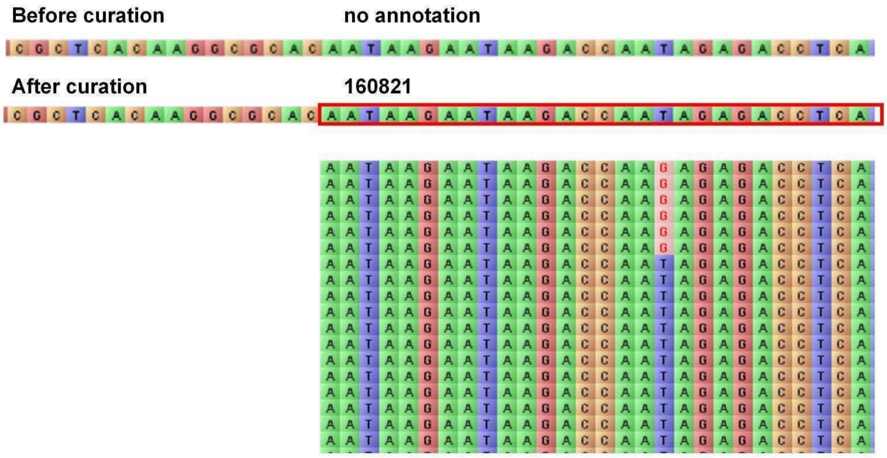

***Rhodiola crenulata*(NC_070303.1)** nad1 exon3 start

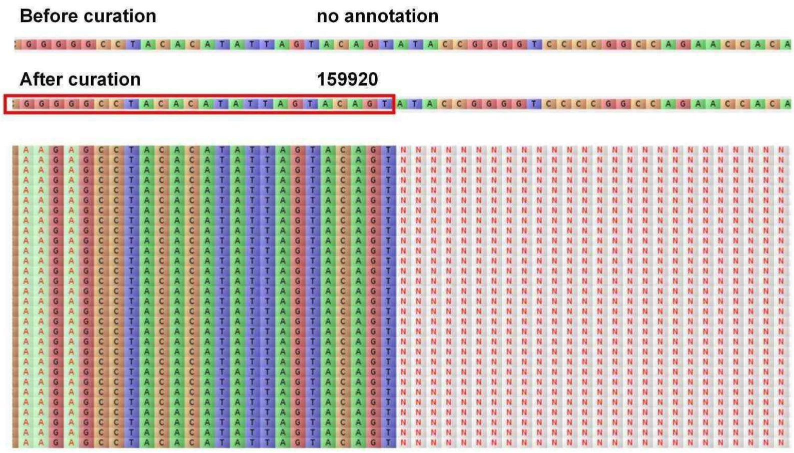

***Rhodiola crenulata*(NC_070303.1)** nad1 exon3 end

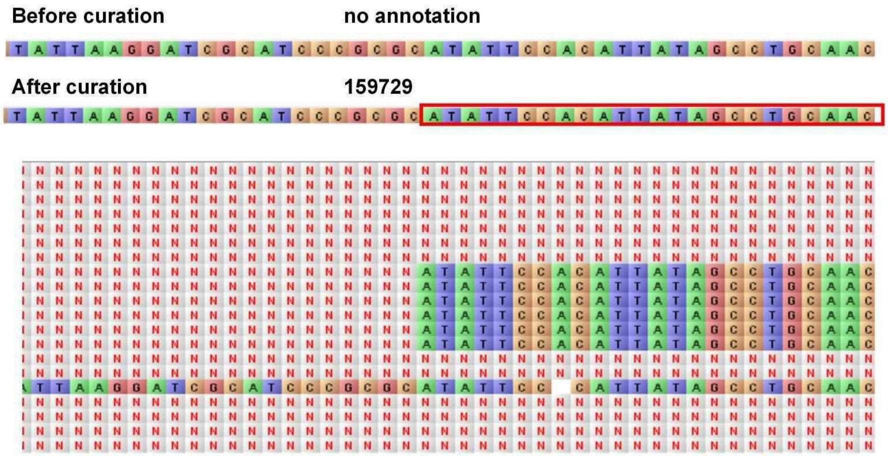

***Rhodiola crenulata*(NC_070303.1)** nad1 exon4 start

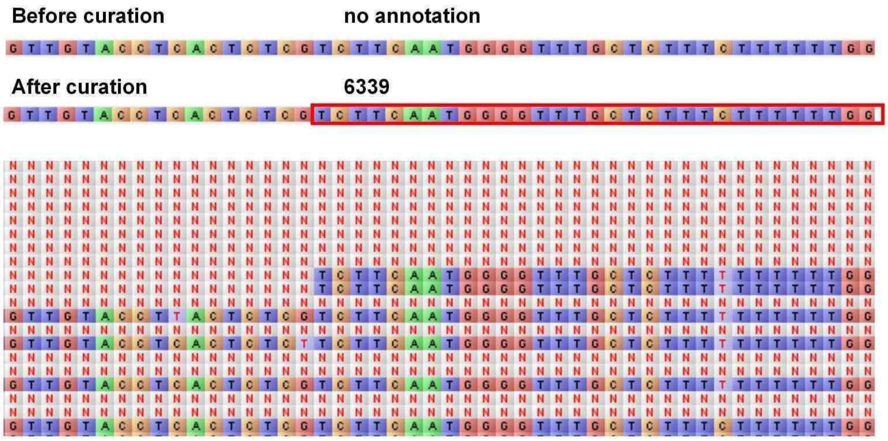

***Rhodiola crenulata*(NC_070303.1)** nad1 exon4 end

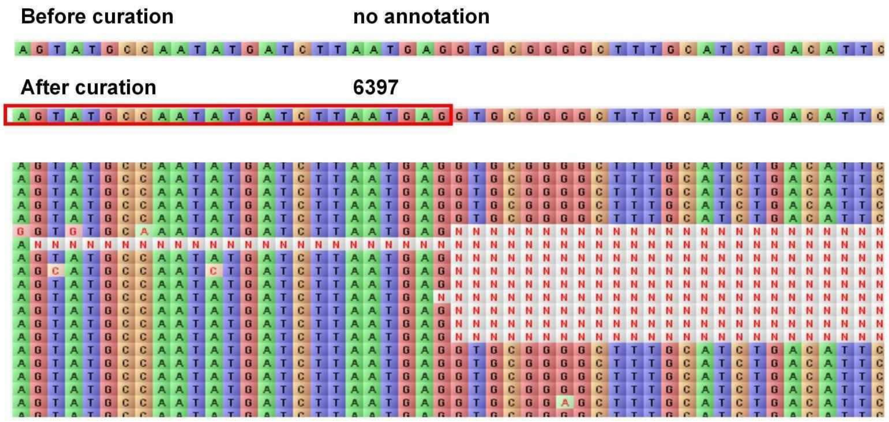

***Rhodiola crenulata*(NC_070303.1)** nad1 exon5 start

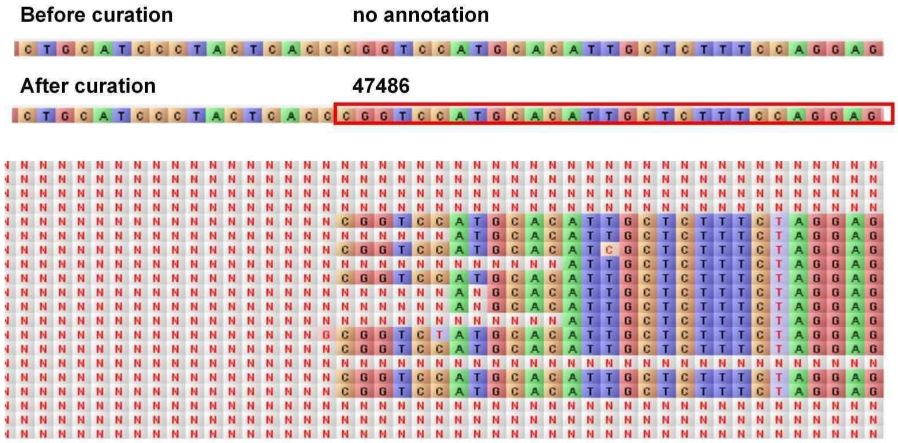

***Rhodiola crenulata*(NC_070303.1)** nad1 exon5 end

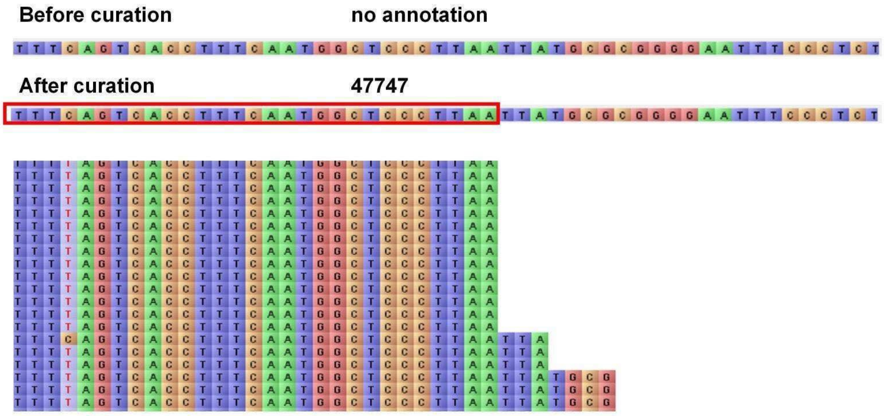

Rhodiola crenulata(**NC_070303.1**) nad2 exon3 end

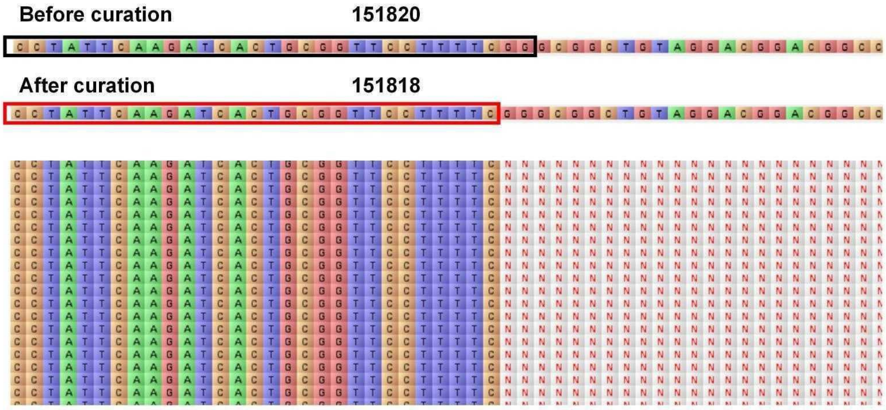

Rhodiola crenulata(**NC_070303.1**) nad2 exon4 start

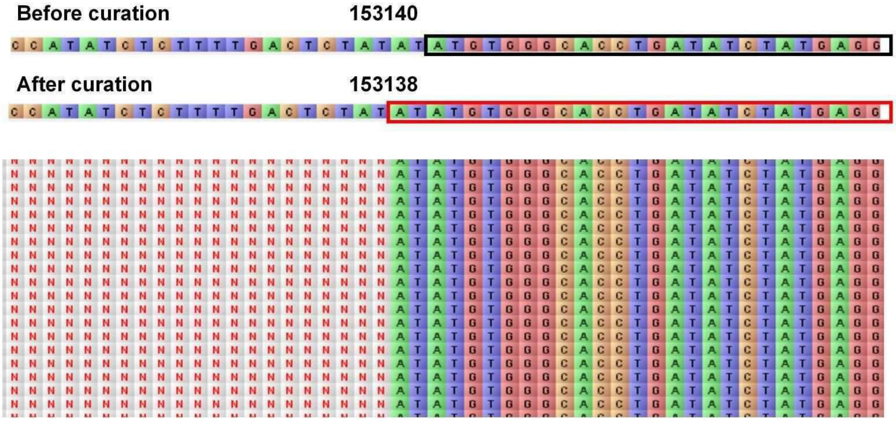

Rhodiola crenulata(**NC_070303.1**) nad2 exon4 end

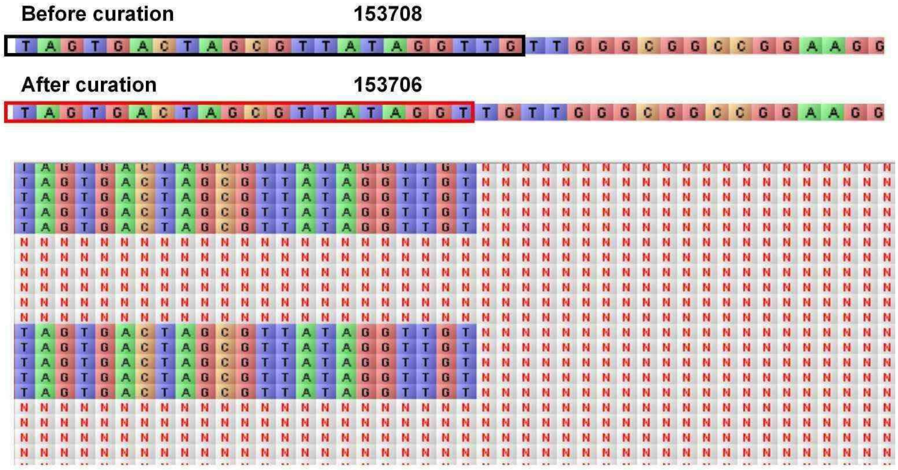

Rhodiola crenulata(NC_070303.1) nad2 exon5 start

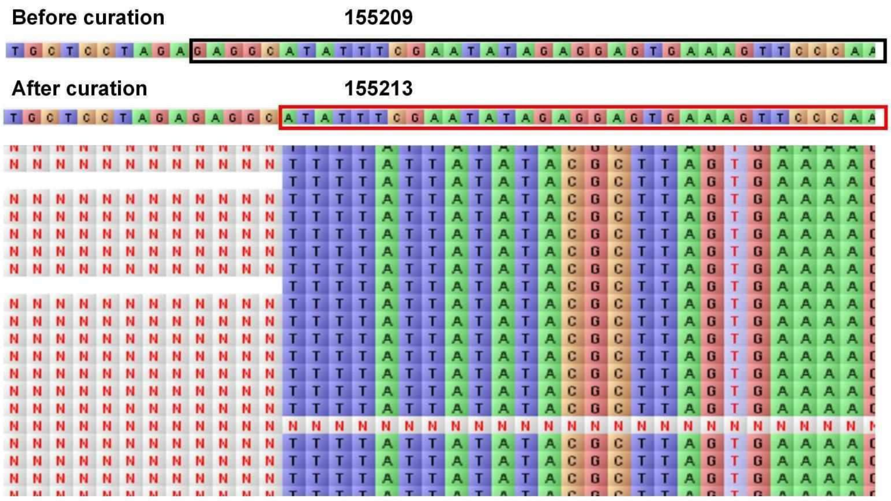

Rhodiola crenulata(NC_070303.1) nad5 exon1 end

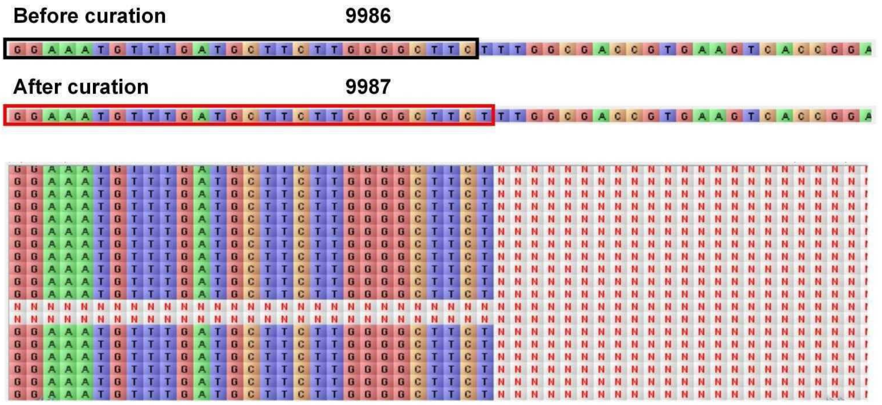

Rhodiola crenulata(NC_070303.1) nad5 exon2 start

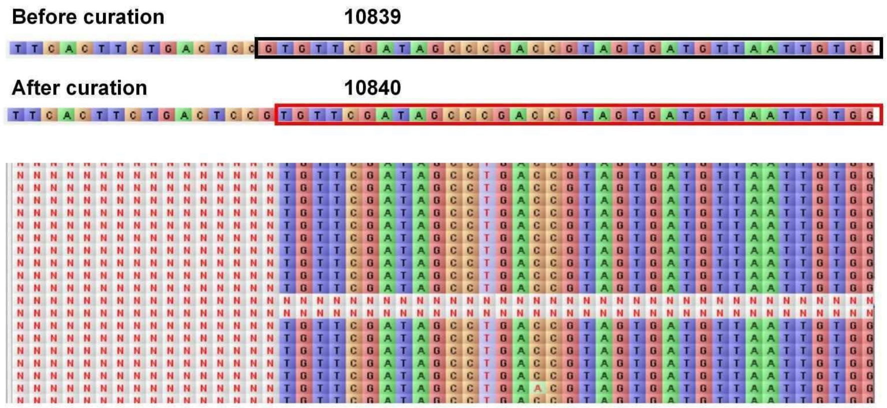

Rhodiola crenulata**(NC_070303.1)** nad5 exon3

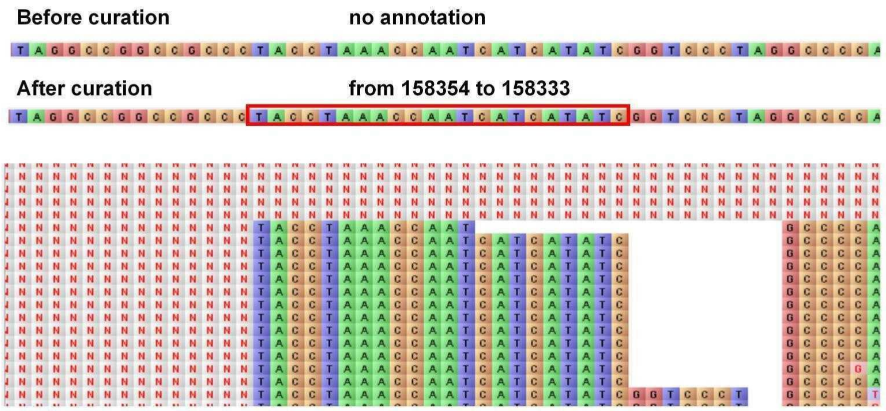

Rhodiola crenulata**(NC_070303.1)** nad5 exon4 start

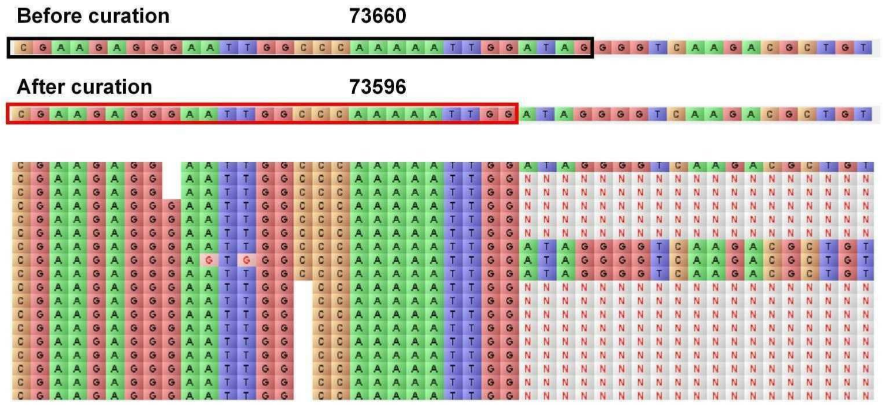

Rhodiola crenulata**(NC_070303.1)** cox2 exon1 start

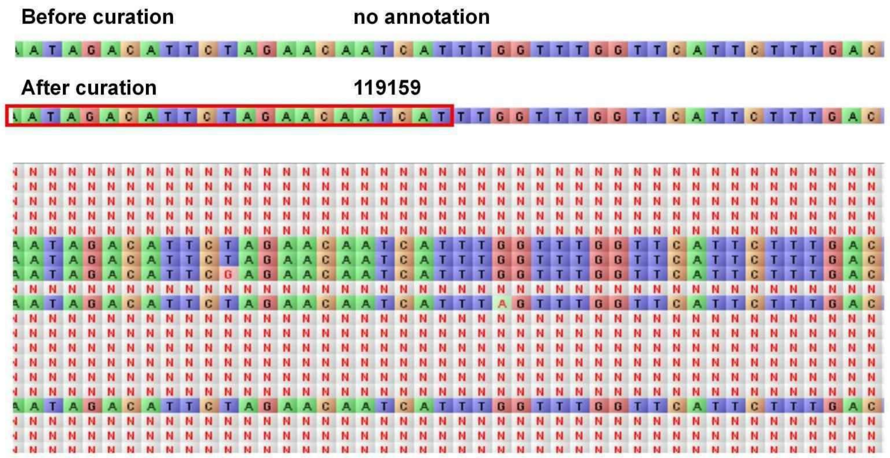

***Rhodiola crenulata*(NC_070303.1)** cox2 exon1 end

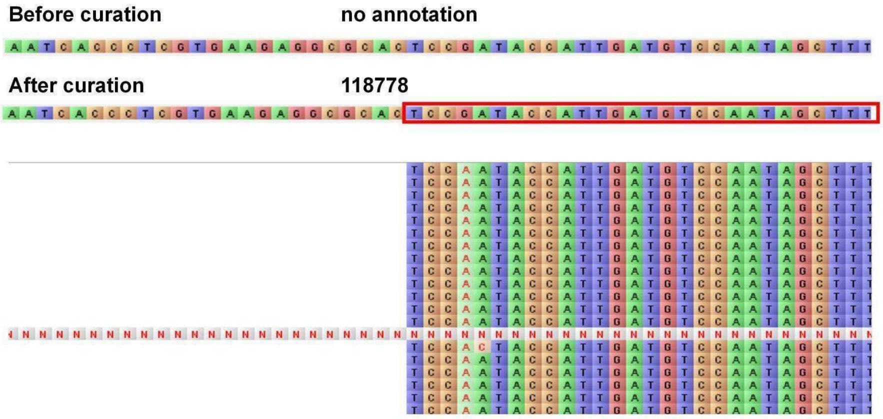

***Rhodiola crenulata*(NC_070303.1)** cox2 exon2 start

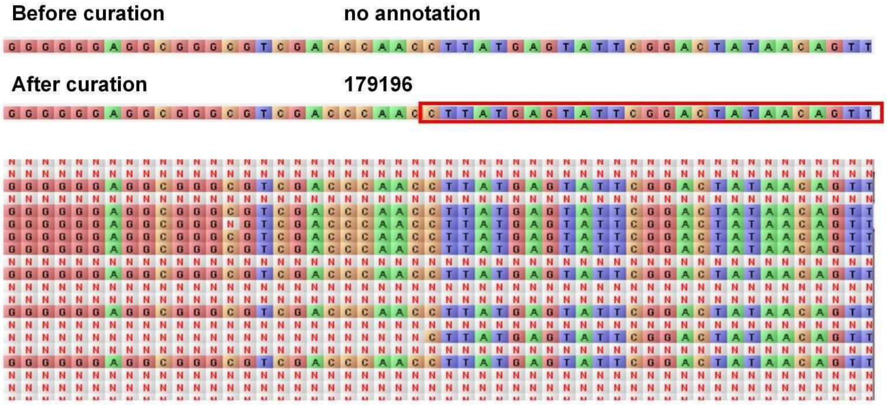

***Rhodiola crenulata*(NC_070303.1)** cox2 exon2 end

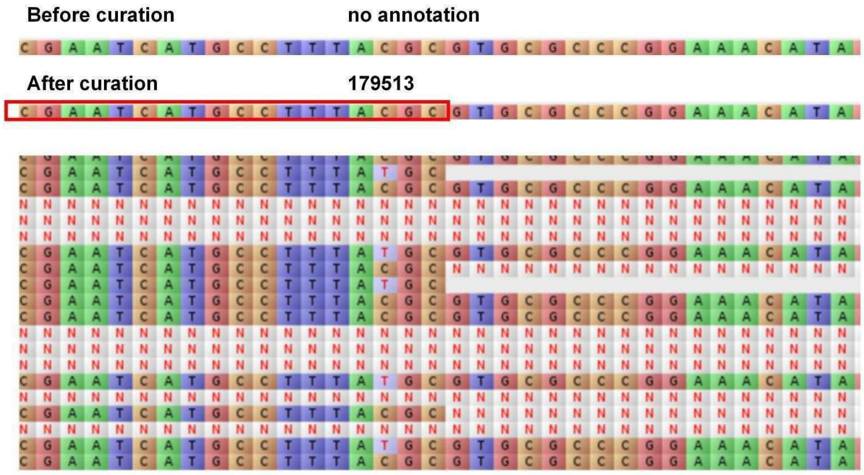

Rhodiola crenulata(**NC_070303.1**) cox2 exon3 start

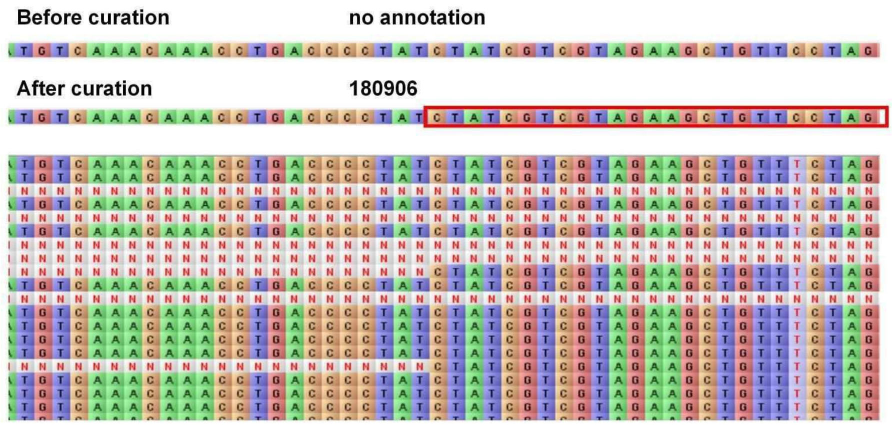

Rhodiola crenulata(**NC_070303.1**) cox2 exon3 end

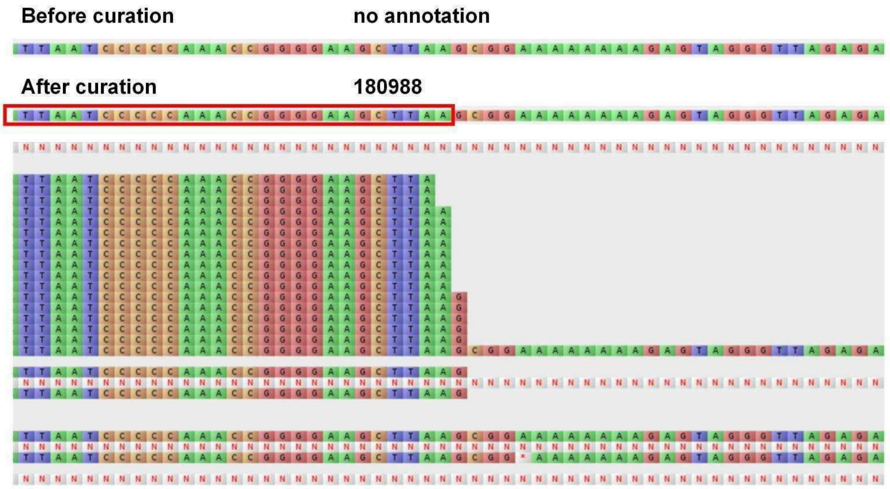

Rhodiola crenulata(**NC_070303.1**) ccmFc exon1 end

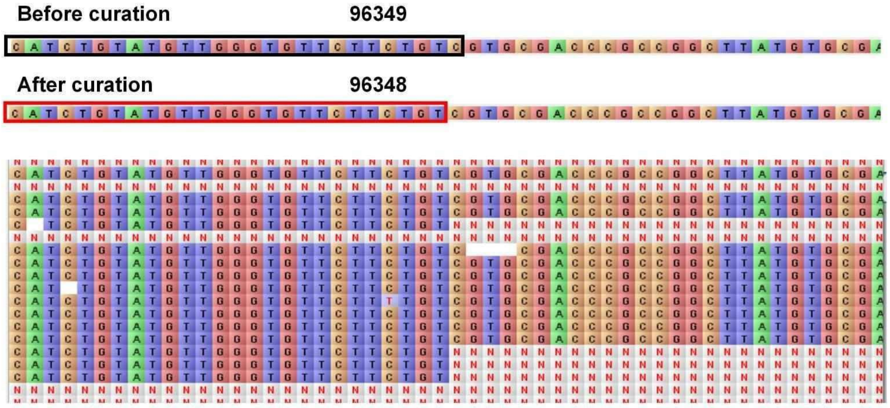

***Rhodiola crenulata*(NC_070303.1)** ccmFc exon2 start

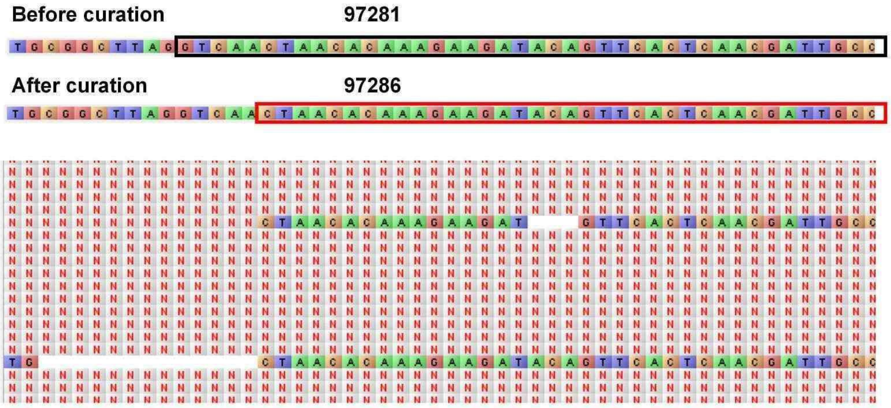

***Rhodiola sacra*(OP312070.1)** nad1 exon2 start

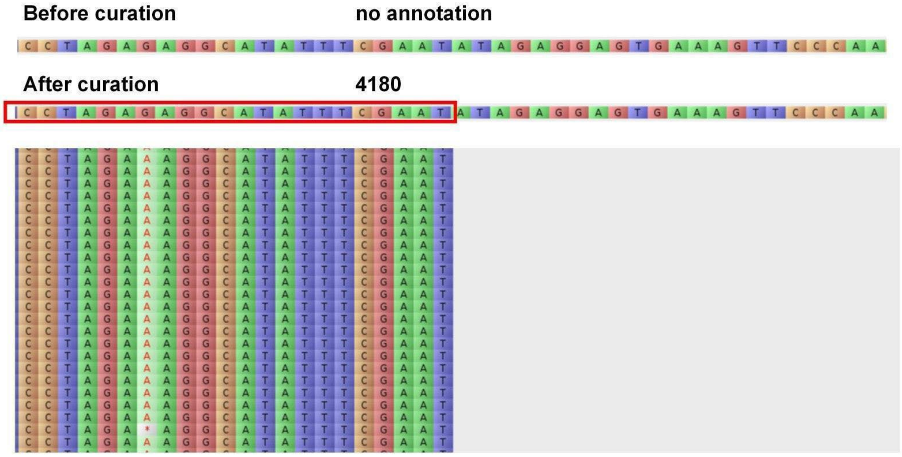

***Rhodiola sacra*(OP312070.1)** nad1 exon2 end

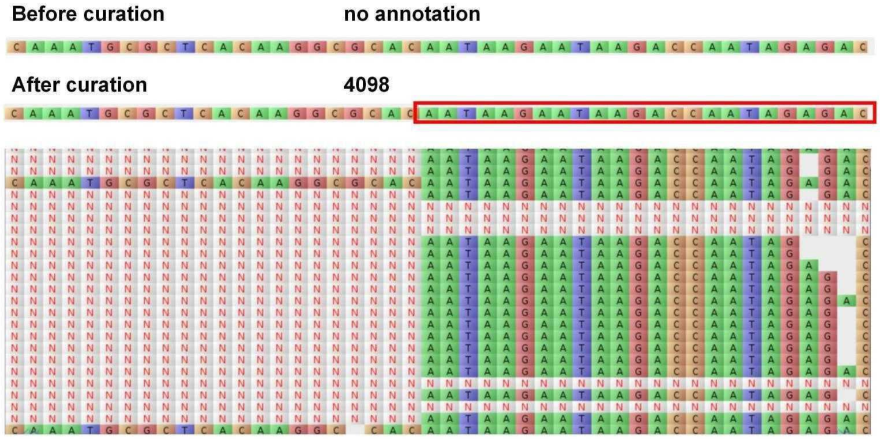

***Rhodiola sacra*(OP312070.1)** nad1 exon3 start

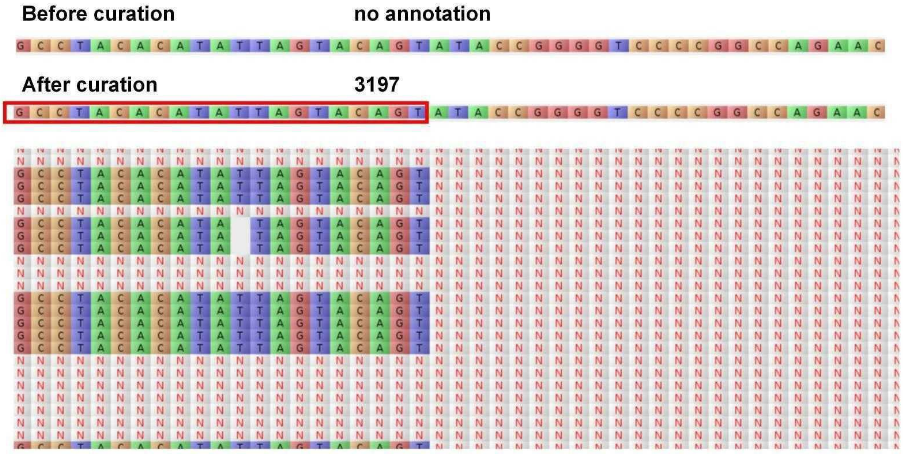

***Rhodiola sacra*(OP312070.1)** nad1 exon3 end

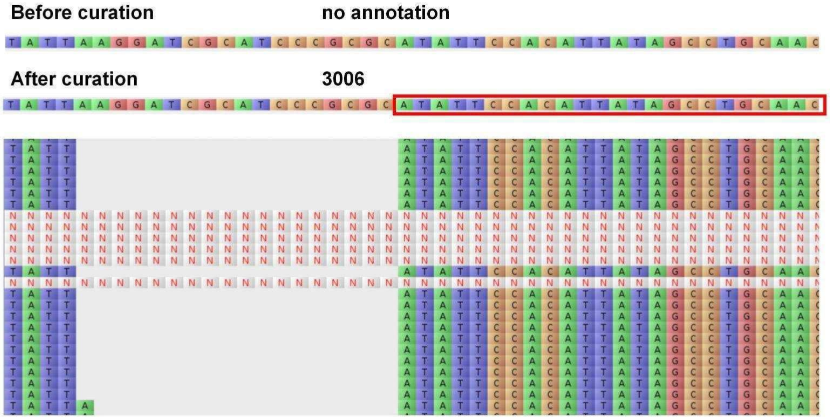

***Rhodiola sacra*(OP312071.1)** nad1 exon4 start

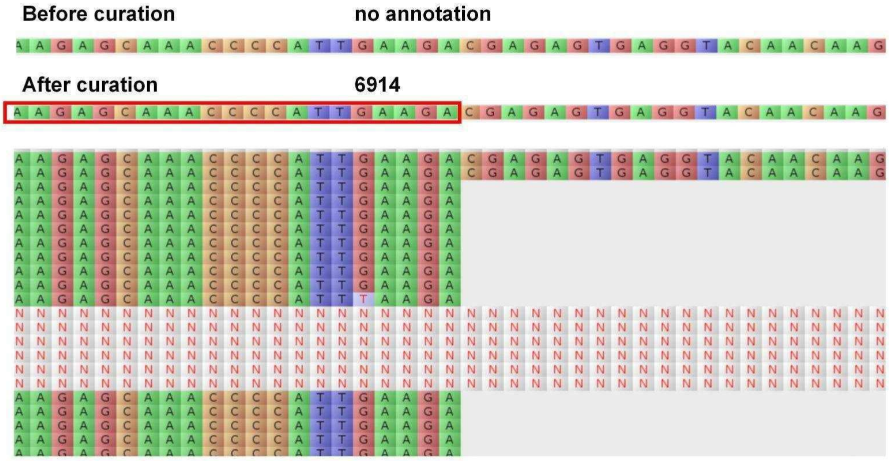

***Rhodiola sacra*(OP312071.1)** nad1 exon4 end

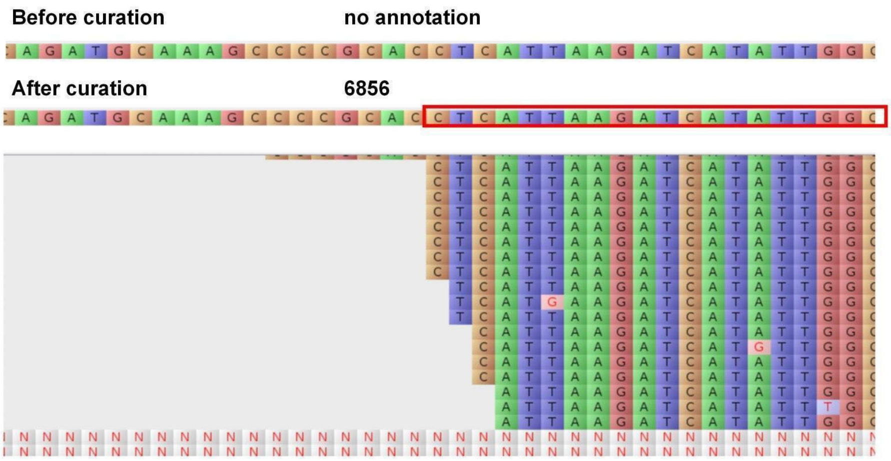

***Rhodiola sacra*(OP312070.1)** nad2 exon3 end

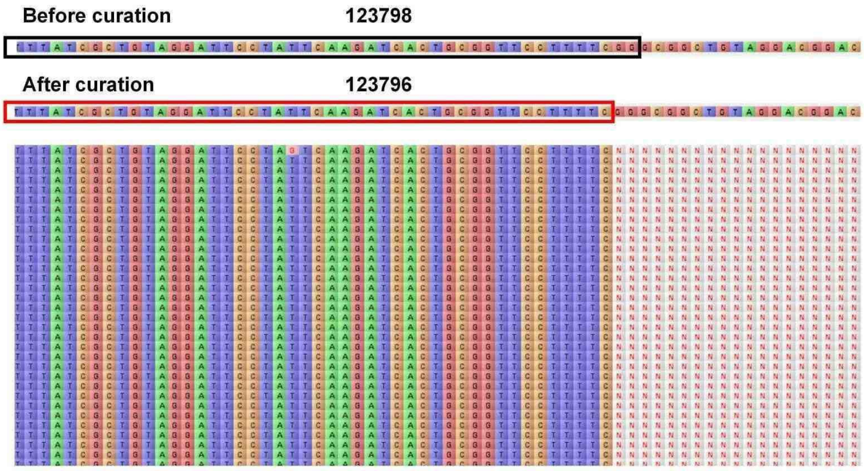

***Rhodiola sacra*(OP312070.1)** nad2 exon4 start

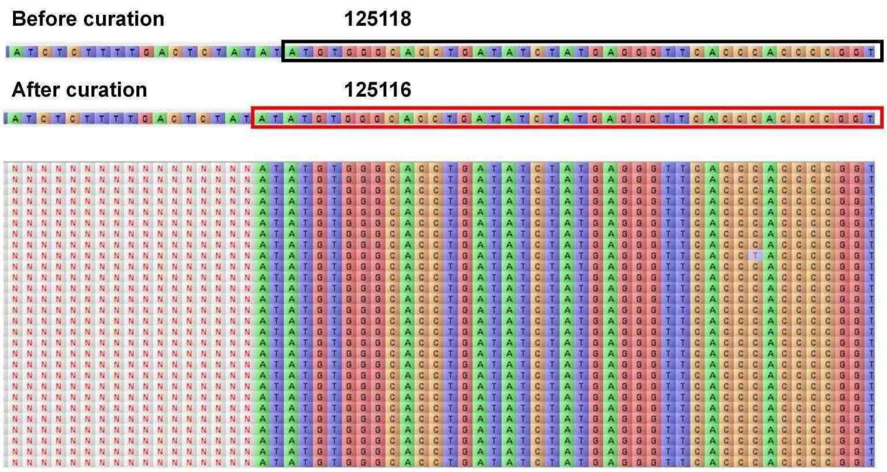

***Rhodiola sacra*(OP312070.1)** nad2 exon4 end

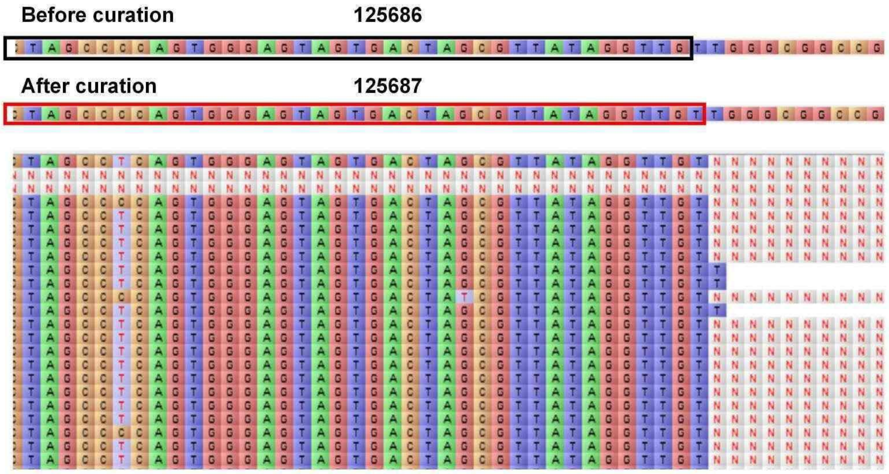

***Rhodiola sacra*(OP312070.1)** nad2 exon5 start

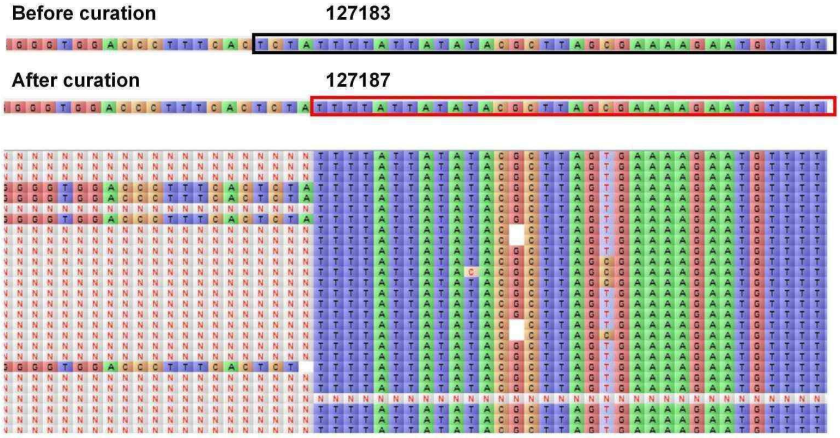

***Rhodiola sacra*(OP312071.1)** nad4 exon3 end

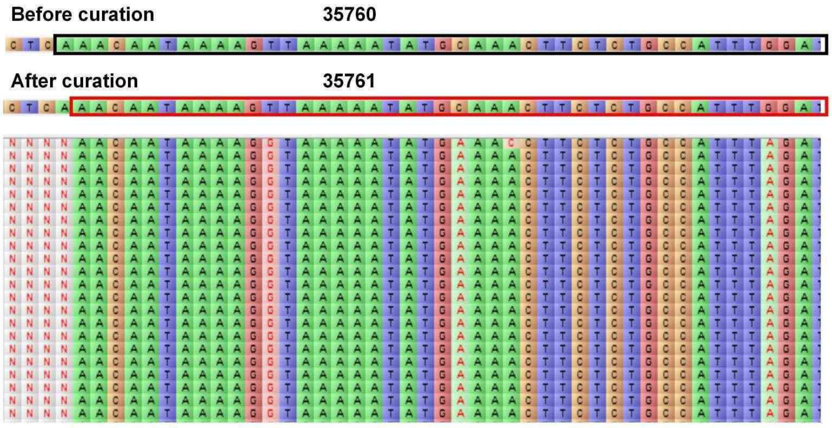

***Rhodiola sacra*(OP312071.1)** nad4 exon4 start

Before curation **38151**

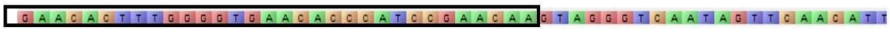

After curation **38152**

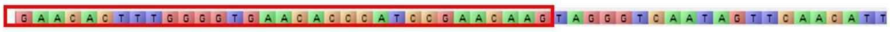

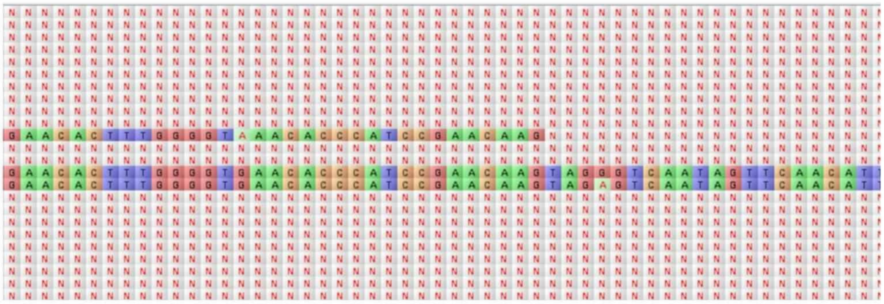

***Rhodiola sacra*(OP312070.1)** nad5 exon3

Before curation **no annotation**

After curation **from 1630 to 1609**

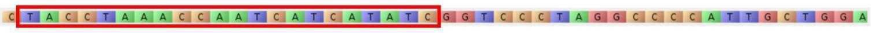

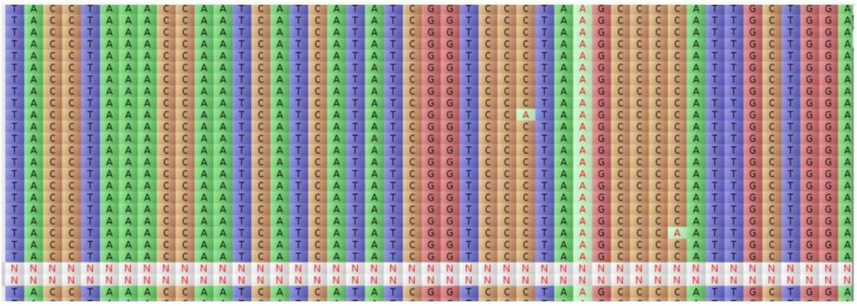

***Rhodiola sacra*(OP312070.1)** nad5 exon4 start

Before curation **no annotation**

After curation **53680**

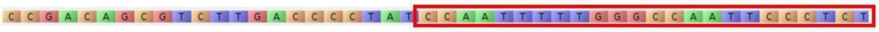

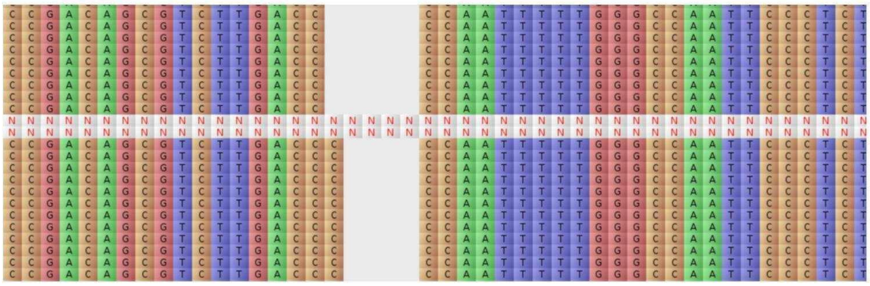

Rhodiola sacra(**OP312070.1**) nad5 exon4 end

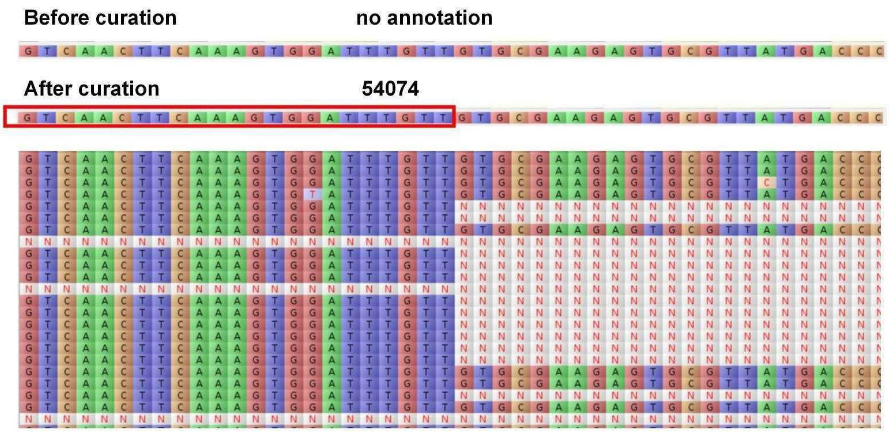

Rhodiola sacra(**OP312070.1**) nad5 exon5 start

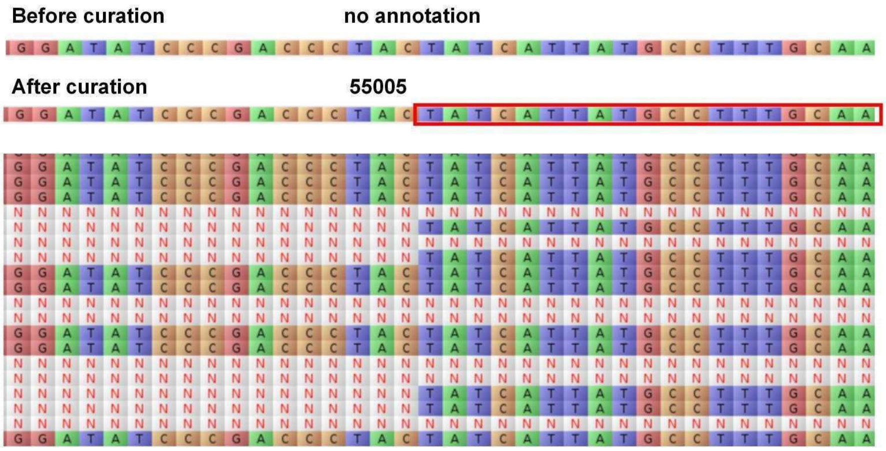

Rhodiola sacra(**OP312070.1**) nad5 exon5 end

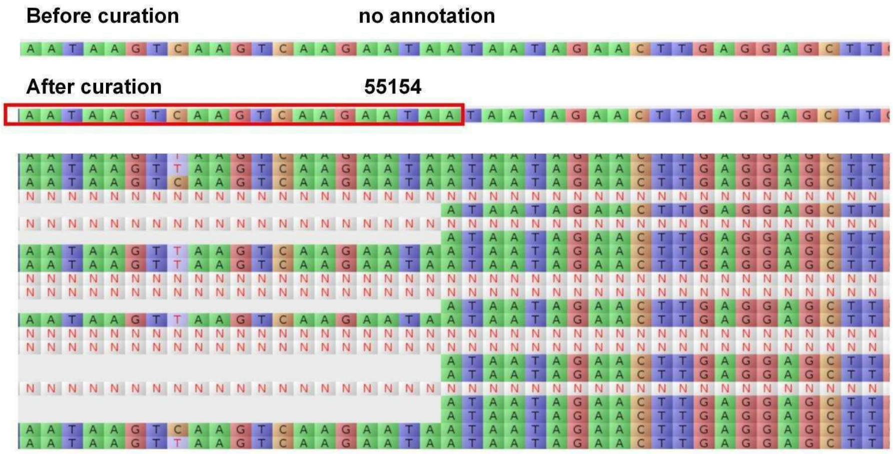

Rhodiola sacra(OP312071.1) ccmfc exon1 end

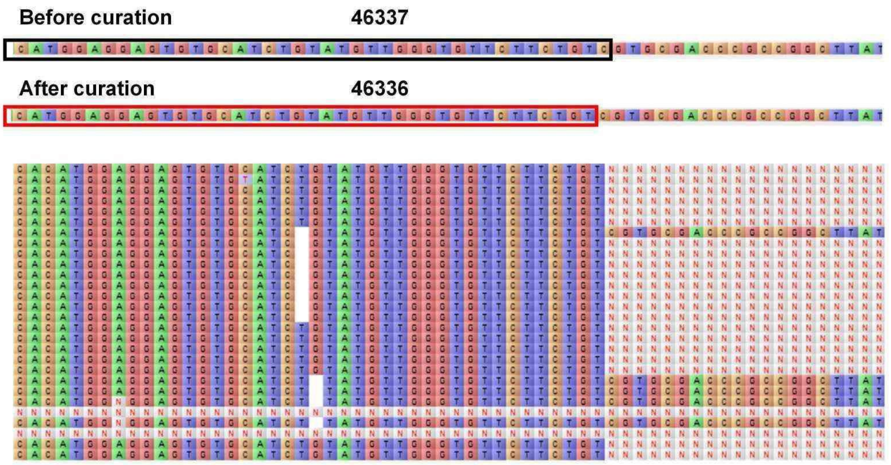

Rhodiola sacra(OP312071.1) ccmfc exon2 start

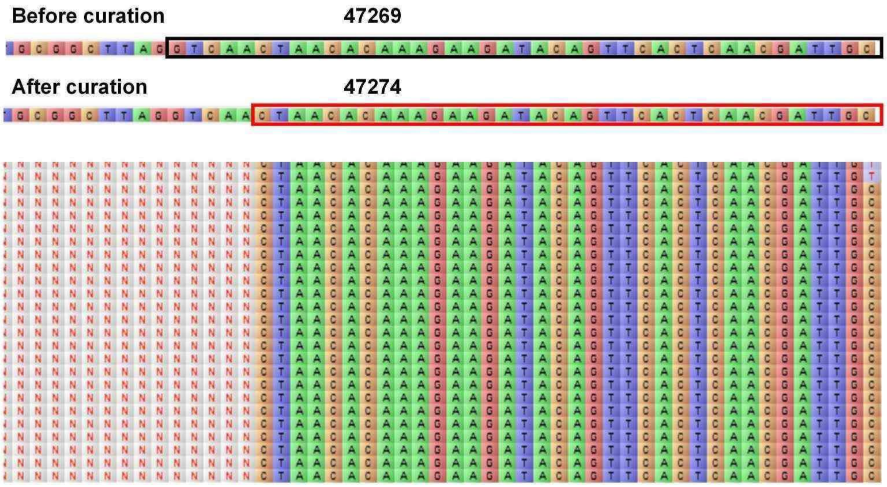

Supplementary Figure S2. Microsynteny analysis of the trans-spliced genes *nad1*, *nad2*, and *nad5* of seven *Rhodiola* species. Red lines indicate exons with multiple copies, while yellow lines denote single-copy exons.

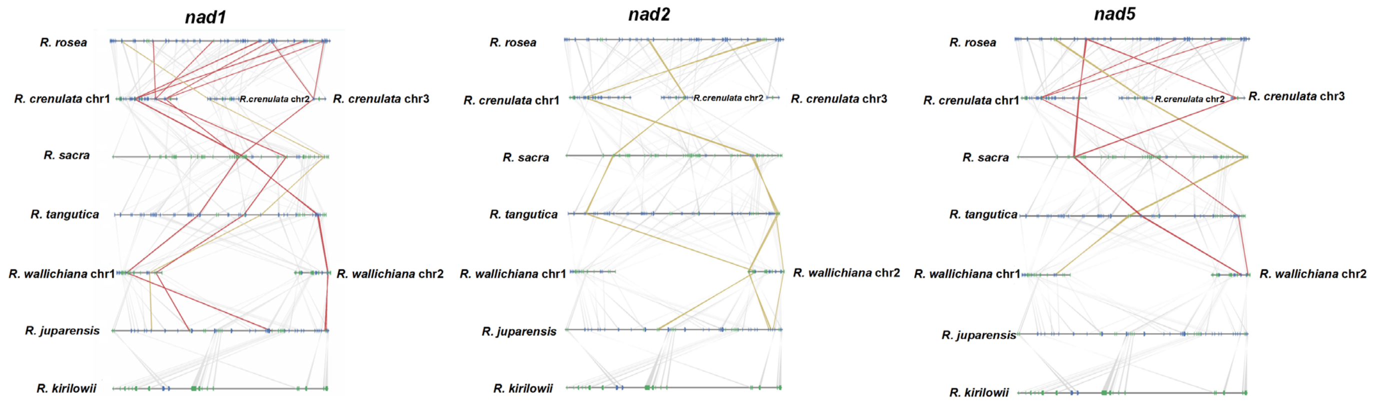

Supplementary Figure S3. Alignment of *nad5*-exon 3 and 150bp upstream and downstream sequences in four *Rhodiola* species.

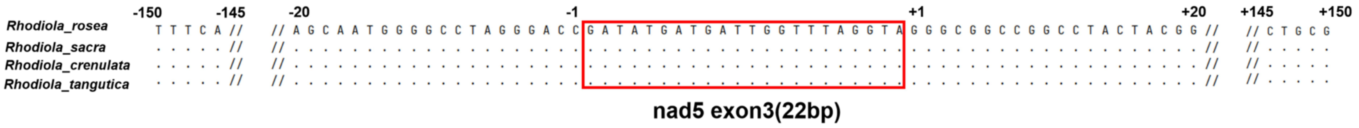

Supplement: Supplementary file 1 [file ijms-27-04440-s001.zip › ijms-4223601 Supplemental Figures.pdf]
