# Supplementary material for: RhoMitoAnnotator and Polypods, Bioinformatics Tools for the Rhodiola Mitochondrial Gene Assembly, Annotation and Phylogenetic Analysis
Source: Int J Mol Sci. 2026 May 15;27(10):4440. doi: 10.3390/ijms27104440 (PMC13207870; doi:10.3390/ijms27104440)
Supplement: Supplementary file 1 [file ijms-27-04440-s001.zip › ijms-4223601 Supplementary Tables.pdf]

Supplementary Table S1. Results of BLAST comparison between mitochondrial gene sequences of the genus *Rhodiola* on NCBI and the full-length lncRNA transcriptome sequencing data of *R. sacra* (similarity greater than 95% or length difference within 5%).

| **Gene** | **Number of blastn reads (similarity greater than 95%)** | **Number of blastn reads (length difference with in 5%)** |
|---|---|---|
| *atp1* | 256791 | 165 |
| *atp4* | 56782 | 9785 |
| *atp6* | 243243 | 44889 |
| *atp8* | 102374 | 14720 |
| *atp9* | 409342 | 318738 |
| *ccmB* | 41041 | 5730 |
| *ccmC* | 129677 | 13990 |
| *ccmFc* | 33563 | 5 |
| *ccmFn* | 98561 | 5 |
| *cob* | 236183 | 9456 |
| *cox1* | 582022 | 4268 |
| *cox2* | 117026 | 10035 |
| *cox3* | 508471 | 31750 |
| *matR* | 86408 | 6 |
| *mttB* | 41169 | 8604 |
| *nad1* | 38421 | 815 |
| *nad2* | 66435 | 1380 |
| *nad3* | 18160 | 10322 |
| *nad4* | 132972 | 57447 |
| *nad4L* | 114827 | 436 |
| *nad5* | 114679 | 124 |
| *nad6* | 74502 | 0 |
| *nad7* | 205425 | 1375 |
| *nad9* | 23210 | 1411 |
| *rpl10* | 7095 | 1265 |
| *rpl16* | 9320 | 331 |
| *rpl5* | 15643 | 1180 |
| *rps12* | 11504 | 836 |
| *rps13* | 116653 | 10326 |
| *rps7* | 20426 | 3516 |

Supplementary Table S2. RNA editing events identified in the mitochondrial PCGs of *R. rosea*.

| Species | Gene | No. | Base | Aa | Triplet pos. | Bases | Codon | Aa change |
|---|---|---|---|---|---|---|---|---|
| *R.rosea* | *atp1* | 1 | 1039 | 347 | 1 | C→U | CCC→UCC | P→S |
| *R.rosea* | *atp1* | 2 | 1168 | 390 | 1 | C→U | CGC→UGC | R→C |
| *R.rosea* | *atp1* | 3 | 1415 | 472 | 2 | C→U | CCA→CUA | P→L |
| *R.rosea* | *atp1* | 4 | 1490 | 497 | 2 | C→U | CCA→CUA | P→L |
| *R.rosea* | *atp4* | 1 | 56 | 19 | 2 | C→U | CCA→CUA | P→L |
| *R.rosea* | *atp4* | 2 | 59 | 20 | 2 | C→U | UCU→UUU | S→F |
| *R.rosea* | *atp4* | 3 | 71 | 24 | 2 | C→U | UCA→UUA | S→L |
| *R.rosea* | *atp4* | 4 | 89 | 30 | 2 | C→U | UCA→UUA | S→L |
| *R.rosea* | *atp4* | 5 | 118 | 40 | 1 | C→U | CGU→UGU | R→C |
| *R.rosea* | *atp4* | 6 | 215 | 72 | 2 | C→U | UCG→UUG | S→L |
| *R.rosea* | *atp4* | 7 | 227 | 76 | 2 | C→U | CCC→CUC | P→L |
| *R.rosea* | *atp4* | 8 | 248 | 83 | 2 | C→U | CCU→CUU | P→L |
| *R.rosea* | *atp4* | 9 | 251 | 84 | 2 | C→U | CCG→CUG | P→L |
| *R.rosea* | *atp4* | 10 | 395 | 132 | 2 | C→U | UCA→UUA | S→L |
| *R.rosea* | *atp4* | 11 | 407 | 136 | 2 | C→U | CCA→CUA | P→L |
| *R.rosea* | *atp4* | 12 | 416 | 139 | 2 | C→U | ACU→AUU | T→I |
| *R.rosea* | *atp6* | 1 | 68 | 23 | 2 | C→U | UCA→UUA | S→L |
| *R.rosea* | *atp6* | 2 | 119 | 40 | 2 | C→U | CCG→CUG | P→L |
| *R.rosea* | *atp6* | 3 | 181 | 61 | 1 | C→U | CGC→UGC | R→C |
| *R.rosea* | *atp6* | 4 | 188 | 63 | 2 | C→U | UCG→UUG | S→L |
| *R.rosea* | *atp6* | 5 | 214 | 72 | 1 | C→U | CGU→UGU | R→C |
| *R.rosea* | *atp6* | 6 | 221 | 74 | 2 | C→U | CCC→CUC | P→L |
| *R.rosea* | *atp6* | 7 | 353 | 118 | 2 | C→U | UCA→UUA | S→L |
| *R.rosea* | *atp6* | 8 | 412 | 138 | 1 | C→U | CCU→UCU | P→S |
| *R.rosea* | *atp6* | 9 | 415 | 139 | 1 | C→U | CAU→UAU | H→Y |
| *R.rosea* | *atp6* | 10 | 437 | 146 | 2 | C→U | UCA→UUA | S→L |
| *R.rosea* | *atp6* | 11 | 479 | 160 | 2 | C→U | UCA→UUA | S→L |
| *R.rosea* | *atp6* | 12 | 551 | 184 | 2 | C→U | CCU→CUU | P→L |
| *R.rosea* | *atp6* | 13 | 587 | 196 | 2 | C→U | CCG→CUG | P→L |
| *R.rosea* | *atp6* | 14 | 608 | 203 | 2 | C→U | UCA→UUA | S→L |
| *R.rosea* | *atp6* | 15 | 616 | 206 | 1 | C→U | CAU→UAU | H→Y |
| *R.rosea* | *atp6* | 16 | 623 | 208 | 2 | C→U | UCU→UUU | S→F |
| *R.rosea* | *atp6* | 17 | 632 | 211 | 2 | C→U | UCA→UUA | S→L |
| *R.rosea* | *atp6* | 18 | 659 | 220 | 2 | C→U | ACA→AUA | T→I |
| *R.rosea* | *atp6* | 19 | 670 | 224 | 1 | C→U | CAA→UAA | Q→* |
| *R.rosea* | *atp8* | 1 | 47 | 16 | 2 | C→U | UCA→UUA | S→L |
| *R.rosea* | *atp8* | 2 | 76 | 26 | 1 | C→U | CCC→UUC | P→F |
| *R.rosea* | *atp8* | | 77 | | 2 | C→U | | |
| *R.rosea* | *atp8* | 3 | 452 | 151 | 2 | C→U | CCA→CUA | P→L |
| *R.rosea* | *atp9* | 1 | 93 | 31 | 3 | C→U | AUC→AUU | I→I |
| *R.rosea* | *ccmB* | 1 | 28 | 10 | 1 | C→U | CAU→UAU | H→Y |
| *R.rosea* | *ccmB* | 2 | 43 | 15 | 1 | C→U | CCC→UCC | P→S |
| *R.rosea* | *ccmB* | 3 | 71 | 24 | 2 | C→U | CCA→CUA | P→L |
| *R.rosea* | *ccmB* | 4 | 80 | 27 | 2 | C→U | UCG→UUG | S→L |
| *R.rosea* | *ccmB* | 5 | 128 | 43 | 2 | C→U | UCA→UUA | S→L |
| *R.rosea* | *ccmB* | 6 | 137 | 46 | 2 | C→U | UCC→UUC | S→F |

| | | | | | | | | |
|---|---|---|---|---|---|---|---|---|
| *R.rosea* | *ccmB* | 7 | 149 | 50 | 2 | C→U | CCG→CUG | P→L |
| *R.rosea* | *ccmB* | 8 | 154 | 52 | 1 | C→U | CGG→UGG | R→W |
| *R.rosea* | *ccmB* | 9 | 160 | 54 | 1 | C→U | CCU→UCU | P→S |
| *R.rosea* | *ccmB* | 10 | 164 | 55 | 2 | C→U | CCG→CUG | P→L |
| *R.rosea* | *ccmB* | 11 | 172 | 58 | 1 | C→U | CCU→UCU | P→S |
| *R.rosea* | *ccmB* | 12 | 179 | 60 | 2 | C→U | CCU→CUU | P→L |
| *R.rosea* | *ccmB* | 13 | 181 | 61 | 1 | C→U | CCC→UCC | P→S |
| *R.rosea* | *ccmB* | 14 | 194 | 65 | 2 | C→U | CCU→CUU | P→L |
| *R.rosea* | *ccmB* | 15 | 286 | 96 | 1 | C→U | CGG→UGG | R→W |
| *R.rosea* | *ccmB* | 16 | 304 | 102 | 1 | C→U | CGU→UGU | R→C |
| *R.rosea* | *ccmB* | 17 | 313 | 105 | 1 | C→U | CGU→UGU | R→C |
| *R.rosea* | *ccmB* | 18 | 367 | 123 | 1 | C→U | CGG→UGG | R→W |
| *R.rosea* | *ccmB* | 19 | 379 | 127 | 1 | C→U | CCA→UUA | P→L |
| *R.rosea* | *ccmB* | | 380 | | 2 | C→U | | |
| *R.rosea* | *ccmB* | 20 | 392 | 131 | 2 | C→U | CCG→CUG | P→L |
| *R.rosea* | *ccmB* | 21 | 424 | 142 | 1 | C→U | CGU→UGU | R→C |
| *R.rosea* | *ccmB* | 22 | 428 | 143 | 2 | C→U | UCG→UUG | S→L |
| *R.rosea* | *ccmB* | 23 | 467 | 156 | 2 | C→U | UCG→UUG | S→L |
| *R.rosea* | *ccmB* | 24 | 475 | 159 | 1 | C→U | CCA→UUA | P→L |
| *R.rosea* | *ccmB* | | 476 | | 2 | C→U | | |
| *R.rosea* | *ccmB* | 25 | 485 | 162 | 2 | C→U | UCA→UUA | S→L |
| *R.rosea* | *ccmB* | 26 | 494 | 165 | 2 | C→U | UCA→UUA | S→L |
| *R.rosea* | *ccmB* | 27 | 502 | 168 | 1 | C→U | CCA→UUA | P→L |
| *R.rosea* | *ccmB* | | 503 | | 2 | C→U | | |
| *R.rosea* | *ccmB* | 28 | 512 | 171 | 2 | C→U | UCU→UUU | S→F |
| *R.rosea* | *ccmB* | 29 | 514 | 172 | 1 | C→U | CGU→UGU | R→C |
| *R.rosea* | *ccmB* | 30 | 548 | 183 | 2 | C→U | CCU→CUU | P→L |
| *R.rosea* | *ccmB* | 31 | 551 | 184 | 2 | C→U | UCA→UUA | S→L |
| *R.rosea* | *ccmB* | 32 | 554 | 185 | 2 | C→U | UCG→UUG | S→L |
| *R.rosea* | *ccmB* | 33 | 566 | 189 | 2 | C→U | UCU→UUU | S→F |
| *R.rosea* | *ccmB* | 34 | 569 | 190 | 2 | C→U | UCU→UUU | S→F |
| *R.rosea* | *ccmB* | 35 | 572 | 191 | 2 | C→U | CCG→CUG | P→L |
| *R.rosea* | *ccmB* | 36 | 596 | 199 | 2 | C→U | UCG→UUG | S→L |
| *R.rosea* | *ccmC* | 1 | 43 | 15 | 1 | C→U | CGG→UGG | R→W |
| *R.rosea* | *ccmC* | 2 | 70 | 24 | 1 | C→U | CAU→UAU | H→Y |
| *R.rosea* | *ccmC* | 3 | 82 | 28 | 1 | C→U | CGG→UGG | R→W |
| *R.rosea* | *ccmC* | 4 | 100 | 34 | 1 | C→U | CUU→UUU | L→F |
| *R.rosea* | *ccmC* | 5 | 128 | 43 | 2 | C→U | CCG→CUG | P→L |
| *R.rosea* | *ccmC* | 6 | 151 | 51 | 1 | C→U | CGG→UGG | R→W |
| *R.rosea* | *ccmC* | 7 | 248 | 83 | 2 | C→U | ACA→AUA | T→I |
| *R.rosea* | *ccmC* | 8 | 266 | 89 | 2 | C→U | UCU→UUU | S→F |
| *R.rosea* | *ccmC* | 9 | 298 | 100 | 1 | C→U | CGG→UGG | R→W |
| *R.rosea* | *ccmC* | 10 | 325 | 109 | 1 | C→U | CGG→UGG | R→W |
| *R.rosea* | *ccmC* | 11 | 362 | 121 | 2 | C→U | UCG→UUG | S→L |
| *R.rosea* | *ccmC* | 12 | 366 | 122 | 3 | C→U | UUC→UUU | F→F |
| *R.rosea* | *ccmC* | 13 | 367 | 123 | 1 | C→U | CUU→UUU | L→F |
| *R.rosea* | *ccmC* | 14 | 385 | 129 | 1 | C→U | CUG→UUG | L→L |
| *R.rosea* | *ccmC* | 15 | 403 | 135 | 1 | C→U | CCU→UCU | P→S |
| *R.rosea* | *ccmC* | 16 | 413 | 138 | 2 | C→U | CCG→CUG | P→L |

| | | | | | | | | |
|---|---|---|---|---|---|---|---|---|
| *R.rosea* | *ccmC* | 17 | 418 | 140 | 1 | C→U | CCU→UCU | P→S |
| *R.rosea* | *ccmC* | 18 | 425 | 142 | 2 | C→U | UCA→UUA | S→L |
| *R.rosea* | *ccmC* | 19 | 430 | 144 | 1 | C→U | CGU→UGU | R→C |
| *R.rosea* | *ccmC* | 20 | 434 | 145 | 2 | C→U | GCU→GUU | A→V |
| *R.rosea* | *ccmC* | 21 | 440 | 147 | 2 | C→U | CCG→CUG | P→L |
| *R.rosea* | *ccmC* | 22 | 488 | 163 | 2 | C→U | UCG→UUG | S→L |
| *R.rosea* | *ccmC* | 23 | 535 | 179 | 1 | C→U | CCU→UCU | P→S |
| *R.rosea* | *ccmC* | 24 | 542 | 181 | 2 | C→U | CCC→CUC | P→L |
| *R.rosea* | *ccmC* | 25 | 572 | 191 | 2 | C→U | UCC→UUC | S→F |
| *R.rosea* | *ccmC* | 26 | 575 | 192 | 2 | C→U | CCC→CUC | P→L |
| *R.rosea* | *ccmC* | 27 | 581 | 194 | 2 | C→U | UCA→UUA | S→L |
| *R.rosea* | *ccmC* | 28 | 586 | 196 | 1 | C→U | CGU→UGU | R→C |
| *R.rosea* | *ccmC* | 29 | 597 | 199 | 3 | C→U | CUC→CUU | L→L |
| *R.rosea* | *ccmC* | 30 | 617 | 206 | 2 | C→U | CCU→CUU | P→L |
| *R.rosea* | *ccmC* | 31 | 623 | 208 | 2 | C→U | CCA→CUA | P→L |
| *R.rosea* | *ccmC* | 32 | 640 | 214 | 1 | C→U | CCC→UCC | P→S |
| *R.rosea* | *ccmFc* | 1 | 38 | 13 | 2 | C→U | UCC→UUC | S→F |
| *R.rosea* | *ccmFc* | 2 | 50 | 17 | 2 | C→U | CCU→CUU | P→L |
| *R.rosea* | *ccmFc* | 3 | 52 | 18 | 1 | C→U | CGU→UGU | R→C |
| *R.rosea* | *ccmFc* | 4 | 103 | 35 | 1 | C→U | CCC→UCC | P→S |
| *R.rosea* | *ccmFc* | 5 | 119 | 40 | 2 | C→U | UCU→UUU | S→F |
| *R.rosea* | *ccmFc* | 6 | 122 | 41 | 2 | C→U | UCC→UUC | S→F |
| *R.rosea* | *ccmFc* | 7 | 146 | 49 | 2 | C→U | CCU→CUU | P→L |
| *R.rosea* | *ccmFc* | 8 | 151 | 51 | 1 | C→U | CCU→UCU | P→S |
| *R.rosea* | *ccmFc* | 9 | 160 | 54 | 1 | C→U | CCU→UCU | P→S |
| *R.rosea* | *ccmFc* | 10 | 296 | 99 | 2 | C→U | UCA→UUA | S→L |
| *R.rosea* | *ccmFc* | 11 | 301 | 101 | 1 | C→U | CGU→UGU | R→C |
| *R.rosea* | *ccmFc* | 12 | 382 | 128 | 1 | C→U | CGU→UGU | R→C |
| *R.rosea* | *ccmFc* | 13 | 397 | 133 | 1 | C→U | CGU→UGU | R→C |
| *R.rosea* | *ccmFc* | 14 | 878 | 293 | 2 | C→U | UCU→UUU | S→F |
| *R.rosea* | *ccmFc* | 15 | 1139 | 380 | 2 | C→U | GCU→GUU | A→V |
| *R.rosea* | *ccmFc* | 16 | 1160 | 387 | 2 | C→U | UCG→UUG | S→L |
| *R.rosea* | *ccmFc* | 17 | 1234 | 412 | 1 | C→U | CGG→UGG | R→W |
| *R.rosea* | *ccmFc* | 18 | 1259 | 420 | 2 | C→U | UCG→UUG | S→L |
| *R.rosea* | *ccmFc* | 19 | 1268 | 423 | 2 | C→U | UCG→UUG | S→L |
| *R.rosea* | *ccmFn* | 1 | 38 | 13 | 2 | C→U | CCG→CUG | P→L |
| *R.rosea* | *ccmFn* | 2 | 98 | 33 | 2 | C→U | CCC→CUC | P→L |
| *R.rosea* | *ccmFn* | 3 | 137 | 46 | 2 | C→U | UCG→UUG | S→L |
| *R.rosea* | *ccmFn* | 4 | 142 | 48 | 1 | C→U | CGU→UGU | R→C |
| *R.rosea* | *ccmFn* | 5 | 151 | 51 | 1 | C→U | CCU→UCU | P→S |
| *R.rosea* | *ccmFn* | 6 | 248 | 83 | 2 | C→U | UCA→UUA | S→L |
| *R.rosea* | *ccmFn* | 7 | 256 | 86 | 1 | C→U | CGG→UGG | R→W |
| *R.rosea* | *ccmFn* | 8 | 263 | 88 | 2 | C→U | CCA→CUA | P→L |
| *R.rosea* | *ccmFn* | 9 | 365 | 122 | 2 | C→U | UCG→UUG | S→L |
| *R.rosea* | *ccmFn* | 10 | 372 | 124 | 3 | C→U | UUC→UUU | F→F |
| *R.rosea* | *ccmFn* | 11 | 707 | 236 | 2 | C→U | CCU→CUU | P→L |
| *R.rosea* | *ccmFn* | 12 | 716 | 239 | 2 | C→U | UCA→UUA | S→L |
| *R.rosea* | *ccmFn* | 13 | 776 | 259 | 2 | C→U | UCA→UUA | S→L |
| *R.rosea* | *ccmFn* | 14 | 788 | 263 | 2 | C→U | CCA→CUA | P→L |

| | | | | | | | | |
|---|---|---|---|---|---|---|---|---|
| *R.rosea* | *ccmFn* | 15 | 803 | 268 | 2 | C→U | UCA→UUA | S→L |
| *R.rosea* | *ccmFn* | 16 | 946 | 316 | 1 | C→U | CGC→UGC | R→C |
| *R.rosea* | *ccmFn* | 17 | 1181 | 394 | 2 | C→U | UCG→UUG | S→L |
| *R.rosea* | *ccmFn* | 18 | 1264 | 422 | 1 | C→U | CGG→UGG | R→W |
| *R.rosea* | *ccmFn* | 19 | 1292 | 431 | 2 | C→U | CCA→CUA | P→L |
| *R.rosea* | *ccmFn* | 20 | 1309 | 437 | 1 | C→U | CAU→UAU | H→Y |
| *R.rosea* | *ccmFn* | 21 | 1324 | 442 | 1 | C→U | CGG→UGG | R→W |
| *R.rosea* | *ccmFn* | 22 | 1342 | 448 | 1 | C→U | CGG→UGG | R→W |
| *R.rosea* | *ccmFn* | 23 | 1375 | 459 | 1 | C→U | CGG→UGG | R→W |
| *R.rosea* | *ccmFn* | 24 | 1456 | 486 | 1 | C→U | CUU→UUU | L→F |
| *R.rosea* | *ccmFn* | 25 | 1460 | 487 | 2 | C→U | CCA→CUA | P→L |
| *R.rosea* | *ccmFn* | 26 | 1472 | 491 | 2 | C→U | UCA→UUA | S→L |
| *R.rosea* | *ccmFn* | 27 | 1507 | 503 | 1 | C→U | CCC→UCC | P→S |
| *R.rosea* | *cob* | 1 | 118 | 40 | 1 | C→U | CCG→UCG | P→S |
| *R.rosea* | *cob* | 2 | 286 | 96 | 1 | C→U | CUC→UUC | L→F |
| *R.rosea* | *cob* | 3 | 325 | 109 | 1 | C→U | CAU→UAU | H→Y |
| *R.rosea* | *cob* | 4 | 358 | 120 | 1 | C→U | CGG→UGG | R→W |
| *R.rosea* | *cob* | 5 | 407 | 136 | 2 | C→U | ACA→AUA | T→I |
| *R.rosea* | *cob* | 6 | 419 | 140 | 2 | C→U | CCA→CUA | P→L |
| *R.rosea* | *cob* | 7 | 568 | 190 | 1 | C→U | CAU→UAU | H→Y |
| *R.rosea* | *cob* | 8 | 580 | 194 | 1 | C→U | CUU→UUU | L→F |
| *R.rosea* | *cob* | 9 | 680 | 227 | 2 | C→U | UCU→UUU | S→F |
| *R.rosea* | *cob* | 10 | 715 | 239 | 1 | C→U | CGG→UGG | R→W |
| *R.rosea* | *cob* | 11 | 725 | 242 | 2 | C→U | UCU→UUU | S→F |
| *R.rosea* | *cob* | 12 | 737 | 246 | 2 | C→U | UCU→UUU | S→F |
| *R.rosea* | *cob* | 13 | 808 | 270 | 1 | C→U | CCC→UCC | P→S |
| *R.rosea* | *cob* | 14 | 853 | 285 | 1 | C→U | CAU→UAU | H→Y |
| *R.rosea* | *cob* | 15 | 908 | 303 | 2 | C→U | CCU→CUU | P→L |
| *R.rosea* | *cob* | 16 | 982 | 328 | 1 | C→U | CAC→UAC | H→Y |
| *R.rosea* | *cob* | 17 | 1084 | 362 | 1 | C→U | CCU→UCU | P→S |
| *R.rosea* | *cox1* | 1 | 196 | 66 | 1 | C→U | CCU→UUU | P→F |
| *R.rosea* | *cox1* | | 197 | | 2 | C→U | | |
| *R.rosea* | *cox1* | 2 | 242 | 81 | 2 | C→U | UCU→UUU | S→F |
| *R.rosea* | *cox1* | 3 | 254 | 85 | 2 | C→U | UCU→UUU | S→F |
| *R.rosea* | *cox1* | 4 | 452 | 151 | 2 | C→U | UCU→UUU | S→F |
| *R.rosea* | *cox1* | 5 | 515 | 172 | 2 | C→U | UCC→UUC | S→F |
| *R.rosea* | *cox1* | 6 | 551 | 184 | 2 | C→U | UCA→UUA | S→L |
| *R.rosea* | *cox1* | 7 | 590 | 197 | 2 | C→U | CCA→CUA | P→L |
| *R.rosea* | *cox1* | 8 | 668 | 223 | 2 | C→U | UCU→UUU | S→F |
| *R.rosea* | *cox1* | 9 | 715 | 239 | 1 | C→U | CGG→UGG | R→W |
| *R.rosea* | *cox1* | 10 | 746 | 249 | 2 | C→U | CCC→CUC | P→L |
| *R.rosea* | *cox1* | 11 | 860 | 287 | 2 | C→U | UCU→UUU | S→F |
| *R.rosea* | *cox1* | 12 | 868 | 290 | 1 | C→U | CGG→UGG | R→W |
| *R.rosea* | *cox1* | 13 | 1079 | 360 | 2 | C→U | CCG→CUG | P→L |
| *R.rosea* | *cox1* | 14 | 1124 | 375 | 2 | C→U | GCG→GUG | A→V |
| *R.rosea* | *cox1* | 15 | 1259 | 420 | 2 | C→U | UCU→UUU | S→F |
| *R.rosea* | *cox1* | 16 | 1274 | 425 | 2 | C→U | CCG→CUG | P→L |
| *R.rosea* | *cox1* | 17 | 1405 | 469 | 1 | C→U | CGU→UGU | R→C |
| *R.rosea* | *cox1* | 18 | 1433 | 478 | 2 | C→U | UCA→UUA | S→L |

| | | | | | | | | |
|---|---|---|---|---|---|---|---|---|
| *R.rosea* | *cox1* | 19 | 1499 | 500 | 2 | C→U | CCG→CUG | P→L |
| *R.rosea* | *cox2* | 1 | 11 | 4 | 2 | C→U | CCU→CUU | P→L |
| *R.rosea* | *cox2* | 2 | 24 | 8 | 3 | C→U | UCC→UCU | S→S |
| *R.rosea* | *cox2* | 3 | 57 | 19 | 3 | C→U | AUC→AUU | I→I |
| *R.rosea* | *cox2* | 4 | 130 | 44 | 1 | C→U | CAU→UAU | H→Y |
| *R.rosea* | *cox2* | 5 | 147 | 49 | 3 | C→U | CUC→CUU | L→L |
| *R.rosea* | *cox2* | 6 | 149 | 50 | 2 | C→U | ACG→AUG | T→M |
| *R.rosea* | *cox2* | 7 | 239 | 80 | 2 | C→U | UCG→UUG | S→L |
| *R.rosea* | *cox2* | 8 | 264 | 88 | 3 | C→U | CCC→CCU | P→P |
| *R.rosea* | *cox2* | 9 | 365 | 122 | 2 | C→U | UCG→UUG | S→L |
| *R.rosea* | *cox2* | 10 | 429 | 143 | 3 | C→U | UAC→UAU | Y→Y |
| *R.rosea* | *cox2* | 11 | 447 | 149 | 3 | C→U | UCC→UCU | S→S |
| *R.rosea* | *cox2* | 12 | 462 | 154 | 3 | C→U | AUC→AUU | I→I |
| *R.rosea* | *cox2* | 13 | 567 | 189 | 3 | C→U | CUC→CUU | L→L |
| *R.rosea* | *cox2* | 14 | 618 | 206 | 3 | C→U | UUC→UUU | F→F |
| *R.rosea* | *cox2* | 15 | 684 | 228 | 3 | C→U | UAC→UAU | Y→Y |
| *R.rosea* | *cox2* | 16 | 707 | 236 | 2 | C→U | UCC→UUC | S→F |
| *R.rosea* | *cox2* | 17 | 728 | 243 | 2 | C→U | UCG→UUG | S→L |
| *R.rosea* | *cox3* | 1 | 245 | 82 | 2 | C→U | CCU→CUU | P→L |
| *R.rosea* | *cox3* | 2 | 257 | 86 | 2 | C→U | UCU→UUU | S→F |
| *R.rosea* | *cox3* | 3 | 263 | 88 | 2 | C→U | CCG→CUG | P→L |
| *R.rosea* | *cox3* | 4 | 289 | 97 | 1 | C→U | CUU→UUU | L→F |
| *R.rosea* | *cox3* | 5 | 298 | 100 | 1 | C→U | CUU→UUU | L→F |
| *R.rosea* | *cox3* | 6 | 304 | 102 | 1 | C→U | CGG→UGG | R→W |
| *R.rosea* | *cox3* | 7 | 311 | 104 | 2 | C→U | UCU→UUU | S→F |
| *R.rosea* | *cox3* | 8 | 314 | 105 | 2 | C→U | UCU→UUU | S→F |
| *R.rosea* | *cox3* | 9 | 413 | 138 | 2 | C→U | CCU→CUU | P→L |
| *R.rosea* | *cox3* | 10 | 422 | 141 | 2 | C→U | CCU→CUU | P→L |
| *R.rosea* | *cox3* | 11 | 512 | 171 | 2 | C→U | UCA→UUA | S→L |
| *R.rosea* | *cox3* | 12 | 527 | 176 | 2 | C→U | UCC→UUC | S→F |
| *R.rosea* | *cox3* | 13 | 602 | 201 | 2 | C→U | UCC→UUC | S→F |
| *R.rosea* | *cox3* | 14 | 653 | 218 | 2 | C→U | UCG→UUG | S→L |
| *R.rosea* | *cox3* | 15 | 764 | 255 | 2 | C→U | CCA→CUA | P→L |
| *R.rosea* | *matR* | 1 | 14 | 5 | 2 | C→U | UCC→UUC | S→F |
| *R.rosea* | *matR* | 2 | 25 | 9 | 1 | C→U | CCC→UCC | P→S |
| *R.rosea* | *matR* | 3 | 175 | 59 | 1 | C→U | CCA→UCA | P→S |
| *R.rosea* | *matR* | 4 | 308 | 103 | 2 | C→U | CCG→CUG | P→L |
| *R.rosea* | *matR* | 5 | 899 | 300 | 2 | C→U | UCA→UUA | S→L |
| *R.rosea* | *matR* | 6 | 1655 | 552 | 2 | C→U | UCC→UUC | S→F |
| *R.rosea* | *matR* | 7 | 1676 | 559 | 2 | C→U | CCU→CUU | P→L |
| *R.rosea* | *matR* | 8 | 1696 | 566 | 1 | C→U | CGC→UGC | R→C |
| *R.rosea* | *matR* | 9 | 1710 | 570 | 3 | C→U | UAC→UAU | Y→Y |
| *R.rosea* | *mttB* | 1 | 31 | 11 | 1 | C→U | CGU→UGU | R→C |
| *R.rosea* | *mttB* | 2 | 43 | 15 | 1 | C→U | CCG→UCG | P→S |
| *R.rosea* | *mttB* | 3 | 59 | 20 | 2 | C→U | UCU→UUU | S→F |
| *R.rosea* | *mttB* | 4 | 62 | 21 | 2 | C→U | CCA→CUA | P→L |
| *R.rosea* | *mttB* | 5 | 109 | 37 | 1 | C→U | CGU→UGU | R→C |
| *R.rosea* | *mttB* | 6 | 133 | 45 | 1 | C→U | CCG→UCG | P→S |
| *R.rosea* | *mttB* | 7 | 193 | 65 | 1 | C→U | CAU→UAU | H→Y |

| | | | | | | | | |
|---|---|---|---|---|---|---|---|---|
| *R.rosea* | *mttB* | 8 | 216 | 72 | 3 | C→U | AUC→AUU | I→I |
| *R.rosea* | *mttB* | 9 | 259 | 87 | 1 | C→U | CUC→UUC | L→F |
| *R.rosea* | *mttB* | 10 | 262 | 88 | 1 | C→U | CAU→UAU | H→Y |
| *R.rosea* | *mttB* | 11 | 277 | 93 | 1 | C→U | CGC→UGC | R→C |
| *R.rosea* | *mttB* | 12 | 282 | 94 | 3 | C→U | UUC→UUU | F→F |
| *R.rosea* | *mttB* | 13 | 284 | 95 | 2 | C→U | UCC→UUC | S→F |
| *R.rosea* | *mttB* | 14 | 304 | 102 | 1 | C→U | CCU→UUU | P→F |
| *R.rosea* | *mttB* | | 305 | | 2 | C→U | | |
| *R.rosea* | *mttB* | 15 | 307 | 103 | 1 | C→U | CCA→UCA | P→S |
| *R.rosea* | *mttB* | 16 | 310 | 104 | 1 | C→U | CGG→UGG | R→W |
| *R.rosea* | *mttB* | 17 | 338 | 113 | 2 | C→U | CCA→CUA | P→L |
| *R.rosea* | *mttB* | 18 | 472 | 158 | 1 | C→U | CGU→UGU | R→C |
| *R.rosea* | *mttB* | 19 | 479 | 160 | 2 | C→U | CCA→CUA | P→L |
| *R.rosea* | *mttB* | 20 | 485 | 162 | 2 | C→U | CCA→CUA | P→L |
| *R.rosea* | *mttB* | 21 | 541 | 181 | 1 | C→U | CCG→UCG | P→S |
| *R.rosea* | *mttB* | 22 | 591 | 197 | 3 | C→U | AUC→AUU | I→I |
| *R.rosea* | *mttB* | 23 | 598 | 200 | 1 | C→U | CGU→UGU | R→C |
| *R.rosea* | *mttB* | 24 | 603 | 201 | 3 | C→U | UUC→UUU | F→F |
| *R.rosea* | *mttB* | 25 | 611 | 204 | 2 | C→U | UCU→UUU | S→F |
| *R.rosea* | *mttB* | 26 | 635 | 212 | 2 | C→U | UCU→UUU | S→F |
| *R.rosea* | *mttB* | 27 | 644 | 215 | 2 | C→U | UCG→UUG | S→L |
| *R.rosea* | *nad1* | 1 | 2 | 1 | 2 | C→U | ACG→AUG | T→M |
| *R.rosea* | *nad1* | 2 | 167 | 56 | 2 | C→U | UCG→UUG | S→L |
| *R.rosea* | *nad1* | 3 | 215 | 72 | 2 | C→U | UCC→UUC | S→F |
| *R.rosea* | *nad1* | 4 | 265 | 89 | 1 | C→U | CGG→UGG | R→W |
| *R.rosea* | *nad1* | 5 | 307 | 103 | 1 | C→U | CUG→UUG | L→L |
| *R.rosea* | *nad1* | 6 | 376 | 126 | 1 | C→U | CGG→UGG | R→W |
| *R.rosea* | *nad1* | 7 | 401 | 134 | 2 | C→U | UCU→UUU | S→F |
| *R.rosea* | *nad1* | 8 | 436 | 146 | 1 | C→U | CCU→UCU | P→S |
| *R.rosea* | *nad1* | 9 | 490 | 164 | 1 | C→U | CCC→UCU | P→S |
| *R.rosea* | *nad1* | | 492 | | 3 | C→U | | |
| *R.rosea* | *nad1* | 10 | 493 | 165 | 1 | C→U | CGU→UGU | R→C |
| *R.rosea* | *nad1* | 11 | 500 | 167 | 2 | C→U | UCG→UUG | S→L |
| *R.rosea* | *nad1* | 12 | 536 | 179 | 2 | C→U | UCC→UUU | S→F |
| *R.rosea* | *nad1* | | 537 | | 3 | C→U | | |
| *R.rosea* | *nad1* | 13 | 571 | 191 | 1 | C→U | CUC→UUU | L→F |
| *R.rosea* | *nad1* | | 573 | | 3 | C→U | | |
| *R.rosea* | *nad1* | 14 | 580 | 194 | 1 | C→U | CGU→UGU | R→C |
| *R.rosea* | *nad1* | 15 | 635 | 212 | 2 | C→U | UCA→UUA | S→L |
| *R.rosea* | *nad1* | 16 | 683 | 228 | 2 | C→U | UCU→UUU | S→F |
| *R.rosea* | *nad1* | 17 | 725 | 242 | 2 | C→U | CCA→CUA | P→L |
| *R.rosea* | *nad1* | 18 | 743 | 248 | 2 | C→U | CCA→CUA | P→L |
| *R.rosea* | *nad1* | 19 | 755 | 252 | 2 | C→U | CCG→CUG | P→L |
| *R.rosea* | *nad1* | 20 | 779 | 260 | 2 | C→U | UCC→UUC | S→F |
| *R.rosea* | *nad1* | 21 | 898 | 300 | 1 | C→U | CGG→UGG | R→W |
| *R.rosea* | *nad1* | 22 | 928 | 310 | 1 | C→U | CGG→UGG | R→W |
| *R.rosea* | *nad1* | 23 | 937 | 313 | 1 | C→U | CCC→UCC | P→S |
| *R.rosea* | *nad1* | 24 | 953 | 318 | 2 | C→U | UCA→UUA | S→L |
| *R.rosea* | *nad2* | 1 | 26 | 9 | 2 | C→U | UCC→UUC | S→F |

| | | | | | | | | |
|---|---|---|---|---|---|---|---|---|
| *R.rosea* | *nad2* | 2 | 223 | 75 | 1 | C→U | CUU→UUU | L→F |
| *R.rosea* | *nad2* | 3 | 308 | 103 | 2 | C→U | UCU→UUU | S→F |
| *R.rosea* | *nad2* | 4 | 311 | 104 | 2 | C→U | UCC→UUC | S→F |
| *R.rosea* | *nad2* | 5 | 341 | 114 | 2 | C→U | UCC→UUC | S→F |
| *R.rosea* | *nad2* | 6 | 356 | 119 | 2 | C→U | CCA→CUA | P→L |
| *R.rosea* | *nad2* | 7 | 361 | 121 | 1 | C→U | CCU→UCU | P→S |
| *R.rosea* | *nad2* | 8 | 367 | 123 | 1 | C→U | CGC→UGC | R→C |
| *R.rosea* | *nad2* | 9 | 394 | 132 | 1 | C→U | CAU→UAU | H→Y |
| *R.rosea* | *nad2* | 10 | 401 | 134 | 2 | C→U | UCA→UUA | S→L |
| *R.rosea* | *nad2* | 11 | 428 | 143 | 2 | C→U | CCU→CUU | P→L |
| *R.rosea* | *nad2* | 12 | 497 | 166 | 2 | C→U | UCG→UUG | S→L |
| *R.rosea* | *nad2* | 13 | 523 | 175 | 1 | C→U | CCC→UCC | P→S |
| *R.rosea* | *nad2* | 14 | 788 | 263 | 2 | C→U | UCU→UUU | S→F |
| *R.rosea* | *nad2* | 15 | 800 | 267 | 2 | C→U | UCA→UUA | S→L |
| *R.rosea* | *nad2* | 16 | 809 | 270 | 2 | C→U | UCU→UUU | S→F |
| *R.rosea* | *nad2* | 17 | 920 | 307 | 2 | C→U | CCU→CUU | P→L |
| *R.rosea* | *nad2* | 18 | 928 | 310 | 1 | C→U | CAU→UAU | H→Y |
| *R.rosea* | *nad2* | 19 | 958 | 320 | 1 | C→U | CGU→UGU | R→C |
| *R.rosea* | *nad2* | 20 | 962 | 321 | 2 | C→U | ACU→AUU | T→I |
| *R.rosea* | *nad2* | 21 | 1028 | 343 | 2 | C→U | UCA→UUA | S→L |
| *R.rosea* | *nad2* | 22 | 1058 | 353 | 2 | C→U | UCA→UUA | S→L |
| *R.rosea* | *nad2* | 23 | 1127 | 376 | 2 | C→U | UCG→UUG | S→L |
| *R.rosea* | *nad2* | 24 | 1246 | 416 | 1 | C→U | CCA→UCA | P→S |
| *R.rosea* | *nad2* | 25 | 1298 | 433 | 2 | C→U | GCG→GUG | A→V |
| *R.rosea* | *nad2* | 26 | 1400 | 467 | 2 | C→U | UCA→UUA | S→L |
| *R.rosea* | *nad2* | 27 | 1403 | 468 | 2 | C→U | UCC→UUC | S→F |
| *R.rosea* | *nad2* | 28 | 1408 | 470 | 1 | C→U | CCA→UUA | P→L |
| *R.rosea* | *nad2* | | 1409 | | 2 | C→U | | |
| *R.rosea* | *nad2* | 29 | 1416 | 472 | 3 | C→U | CCC→CCU | P→P |
| *R.rosea* | *nad2* | 30 | 1457 | 486 | 2 | C→U | UCA→UUA | S→L |
| *R.rosea* | *nad3* | 1 | 5 | 2 | 2 | C→U | UCA→UUA | S→L |
| *R.rosea* | *nad3* | 2 | 44 | 15 | 2 | C→U | CCG→CUG | P→L |
| *R.rosea* | *nad3* | 3 | 62 | 21 | 2 | C→U | CCA→CUA | P→L |
| *R.rosea* | *nad3* | 4 | 80 | 27 | 2 | C→U | CCA→CUA | P→L |
| *R.rosea* | *nad3* | 5 | 124 | 42 | 1 | C→U | CAU→UAU | H→Y |
| *R.rosea* | *nad3* | 6 | 137 | 46 | 2 | C→U | UCC→UUC | S→F |
| *R.rosea* | *nad3* | 7 | 146 | 49 | 2 | C→U | UCC→UUC | S→F |
| *R.rosea* | *nad3* | 8 | 208 | 70 | 1 | C→U | CCU→UUU | P→F |
| *R.rosea* | *nad3* | | 209 | | 2 | C→U | | |
| *R.rosea* | *nad3* | 9 | 215 | 72 | 2 | C→U | CCG→CUG | P→L |
| *R.rosea* | *nad3* | 10 | 230 | 77 | 2 | C→U | UCC→UUC | S→F |
| *R.rosea* | *nad3* | 11 | 247 | 83 | 1 | C→U | CCU→UCU | P→S |
| *R.rosea* | *nad3* | 12 | 251 | 84 | 2 | C→U | CCC→CUC | P→L |
| *R.rosea* | *nad3* | 13 | 266 | 89 | 2 | C→U | CCG→CUG | P→L |
| *R.rosea* | *nad3* | 14 | 275 | 92 | 2 | C→U | UCU→UUU | S→F |
| *R.rosea* | *nad3* | 15 | 317 | 106 | 2 | C→U | UCU→UUU | S→F |
| *R.rosea* | *nad3* | 16 | 344 | 115 | 2 | C→U | UCG→UUG | S→L |
| *R.rosea* | *nad3* | 17 | 349 | 117 | 1 | C→U | CGG→UGG | R→W |
| *R.rosea* | *nad4* | 1 | 50 | 17 | 2 | C→U | CCG→CUG | P→L |

| | | | | | | | | |
|---|---|---|---|---|---|---|---|---|
| *R.rosea* | *nad4* | 2 | 74 | 25 | 2 | C→U | ACU→AUU | T→I |
| *R.rosea* | *nad4* | 3 | 77 | 26 | 2 | C→U | CCU→CUU | P→L |
| *R.rosea* | *nad4* | 4 | 84 | 28 | 3 | C→U | UUC→UUU | F→F |
| *R.rosea* | *nad4* | 5 | 107 | 36 | 2 | C→U | CCG→CUG | P→L |
| *R.rosea* | *nad4* | 6 | 154 | 52 | 1 | C→U | CCC→UCC | P→S |
| *R.rosea* | *nad4* | 7 | 158 | 53 | 2 | C→U | CCU→CUU | P→L |
| *R.rosea* | *nad4* | 8 | 164 | 55 | 2 | C→U | CCU→CUU | P→L |
| *R.rosea* | *nad4* | 9 | 166 | 56 | 1 | C→U | CGG→UGG | R→W |
| *R.rosea* | *nad4* | 10 | 197 | 66 | 2 | C→U | UCU→UUU | S→F |
| *R.rosea* | *nad4* | 11 | 317 | 106 | 2 | C→U | UCA→UUA | S→L |
| *R.rosea* | *nad4* | 12 | 362 | 121 | 2 | C→U | GCA→GUA | A→V |
| *R.rosea* | *nad4* | 13 | 368 | 123 | 2 | C→U | UCU→UUU | S→F |
| *R.rosea* | *nad4* | 14 | 376 | 126 | 1 | C→U | CGU→UGU | R→C |
| *R.rosea* | *nad4* | 15 | 403 | 135 | 1 | C→U | CGC→UGC | R→C |
| *R.rosea* | *nad4* | 16 | 416 | 139 | 2 | C→U | CCU→CUU | P→L |
| *R.rosea* | *nad4* | 17 | 433 | 145 | 1 | C→U | CUU→UUU | L→F |
| *R.rosea* | *nad4* | 18 | 436 | 146 | 1 | C→U | CCC→UUC | P→F |
| *R.rosea* | *nad4* | | 437 | | 2 | C→U | | |
| *R.rosea* | *nad4* | 19 | 449 | 150 | 2 | C→U | CCA→CUA | P→L |
| *R.rosea* | *nad4* | 20 | 608 | 203 | 2 | C→U | UCA→UUA | S→L |
| *R.rosea* | *nad4* | 21 | 659 | 220 | 2 | C→U | UCU→UUU | S→F |
| *R.rosea* | *nad4* | 22 | 767 | 256 | 2 | C→U | CCU→CUU | P→L |
| *R.rosea* | *nad4* | 23 | 836 | 279 | 2 | C→U | UCC→UUC | S→F |
| *R.rosea* | *nad4* | 24 | 856 | 286 | 1 | C→U | CCA→UUA | P→L |
| *R.rosea* | *nad4* | | 857 | | 2 | C→U | | |
| *R.rosea* | *nad4* | 25 | 887 | 296 | 2 | C→U | UCG→UUG | S→L |
| *R.rosea* | *nad4* | 26 | 896 | 299 | 2 | C→U | UCA→UUA | S→L |
| *R.rosea* | *nad4* | 27 | 977 | 326 | 2 | C→U | CCG→CUG | P→L |
| *R.rosea* | *nad4* | 28 | 1006 | 336 | 1 | C→U | CUA→UUA | L→L |
| *R.rosea* | *nad4* | 29 | 1010 | 337 | 2 | C→U | CCG→CUG | P→L |
| *R.rosea* | *nad4* | 30 | 1016 | 339 | 2 | C→U | UCA→UUA | S→L |
| *R.rosea* | *nad4* | 31 | 1033 | 345 | 1 | C→U | CCU→UCU | P→S |
| *R.rosea* | *nad4* | 32 | 1101 | 367 | 3 | C→U | UAC→UAU | Y→Y |
| *R.rosea* | *nad4* | 33 | 1129 | 377 | 1 | C→U | CUC→UUC | L→F |
| *R.rosea* | *nad4* | 34 | 1142 | 381 | 2 | C→U | UCC→UUC | S→F |
| *R.rosea* | *nad4* | 35 | 1148 | 383 | 2 | C→U | UCU→UUU | S→F |
| *R.rosea* | *nad4* | 36 | 1151 | 384 | 2 | C→U | UCC→UUC | S→F |
| *R.rosea* | *nad4* | 37 | 1172 | 391 | 2 | C→U | UCA→UUA | S→L |
| *R.rosea* | *nad4* | 38 | 1211 | 404 | 2 | C→U | UCA→UUA | S→L |
| *R.rosea* | *nad4* | 39 | 1307 | 436 | 2 | C→U | GCG→GUG | A→V |
| *R.rosea* | *nad4* | 40 | 1355 | 452 | 2 | C→U | CCA→CUA | P→L |
| *R.rosea* | *nad4* | 41 | 1373 | 458 | 2 | C→U | UCC→UUC | S→F |
| *R.rosea* | *nad4* | 42 | 1405 | 469 | 1 | C→U | CGG→UGG | R→W |
| *R.rosea* | *nad4* | 43 | 1417 | 473 | 1 | C→U | CAC→UAC | H→Y |
| *R.rosea* | *nad4* | 44 | 1433 | 478 | 2 | C→U | CCG→CUG | P→L |
| *R.rosea* | *nad4* | 45 | 1438 | 480 | 1 | C→U | CGC→UGC | R→C |
| *R.rosea* | *nad4L* | 1 | 2 | 1 | 2 | C→U | ACG→AUG | T→M |
| *R.rosea* | *nad4L* | 2 | 41 | 14 | 2 | C→U | UCU→UUU | S→F |
| *R.rosea* | *nad4L* | 3 | 55 | 19 | 1 | C→U | CGG→UGG | R→W |

| | | | | | | | | |
|---|---|---|---|---|---|---|---|---|
| *R.rosea* | *nad4L* | 4 | 86 | 29 | 2 | C→U | CCU→CUU | P→L |
| *R.rosea* | *nad4L* | 5 | 100 | 34 | 1 | C→U | CCA→UCA | P→S |
| *R.rosea* | *nad4L* | 6 | 110 | 37 | 2 | C→U | UCA→UUA | S→L |
| *R.rosea* | *nad4L* | 7 | 131 | 44 | 2 | C→U | UCG→UUG | S→L |
| *R.rosea* | *nad4L* | 8 | 158 | 53 | 2 | C→U | UCG→UUG | S→L |
| *R.rosea* | *nad4L* | 9 | 179 | 60 | 2 | C→U | UCA→UUA | S→L |
| *R.rosea* | *nad4L* | 10 | 188 | 63 | 2 | C→U | UCA→UUA | S→L |
| *R.rosea* | *nad4L* | 11 | 197 | 66 | 2 | C→U | CCA→CUA | P→L |
| *R.rosea* | *nad4L* | 12 | 281 | 94 | 2 | C→U | UCU→UUU | S→F |
| *R.rosea* | *nad5* | 1 | 110 | 37 | 2 | C→U | UCA→UUA | S→L |
| *R.rosea* | *nad5* | 2 | 155 | 52 | 2 | C→U | UCG→UUG | S→L |
| *R.rosea* | *nad5* | 3 | 242 | 81 | 2 | C→U | CCG→CUG | P→L |
| *R.rosea* | *nad5* | 4 | 272 | 91 | 2 | C→U | UCC→UUC | S→F |
| *R.rosea* | *nad5* | 5 | 315 | 105 | 3 | C→U | UCC→UCU | S→S |
| *R.rosea* | *nad5* | 6 | 358 | 120 | 1 | C→U | CCU→UUU | P→F |
| *R.rosea* | *nad5* | | 359 | | 2 | C→U | | |
| *R.rosea* | *nad5* | 7 | 374 | 125 | 2 | C→U | CCA→CUA | P→L |
| *R.rosea* | *nad5* | 8 | 398 | 133 | 2 | C→U | UCU→UUU | S→F |
| *R.rosea* | *nad5* | 9 | 539 | 180 | 2 | C→U | CCU→CUU | P→L |
| *R.rosea* | *nad5* | 10 | 548 | 183 | 2 | C→U | UCG→UUG | S→L |
| *R.rosea* | *nad5* | 11 | 553 | 185 | 1 | C→U | CGU→UGU | R→C |
| *R.rosea* | *nad5* | 12 | 598 | 200 | 1 | C→U | CGU→UGU | R→C |
| *R.rosea* | *nad5* | 13 | 608 | 203 | 2 | C→U | GCC→GUC | A→V |
| *R.rosea* | *nad5* | 14 | 629 | 210 | 2 | C→U | UCU→UUU | S→F |
| *R.rosea* | *nad5* | 15 | 676 | 226 | 1 | C→U | CUU→UUU | L→F |
| *R.rosea* | *nad5* | 16 | 713 | 238 | 2 | C→U | UCG→UUG | S→L |
| *R.rosea* | *nad5* | 17 | 725 | 242 | 2 | C→U | UCA→UUA | S→L |
| *R.rosea* | *nad5* | 18 | 835 | 279 | 1 | C→U | CCA→UCA | P→S |
| *R.rosea* | *nad5* | 19 | 863 | 288 | 2 | C→U | UCU→UUU | S→F |
| *R.rosea* | *nad5* | 20 | 875 | 292 | 2 | C→U | ACG→AUG | T→M |
| *R.rosea* | *nad5* | 21 | 1184 | 395 | 2 | C→U | CCA→CUA | P→L |
| *R.rosea* | *nad5* | 22 | 1310 | 437 | 2 | C→U | UCA→UUA | S→L |
| *R.rosea* | *nad5* | 23 | 1550 | 517 | 2 | C→U | ACC→AUC | T→I |
| *R.rosea* | *nad5* | 24 | 1568 | 523 | 2 | C→U | CCG→CUG | P→L |
| *R.rosea* | *nad5* | 25 | 1589 | 530 | 2 | C→U | UCU→UUU | S→F |
| *R.rosea* | *nad5* | 26 | 1610 | 537 | 2 | C→U | CCC→CUC | P→L |
| *R.rosea* | *nad5* | 27 | 1895 | 632 | 2 | C→U | UCA→UUA | S→L |
| *R.rosea* | *nad5* | 28 | 1916 | 639 | 2 | C→U | UCU→UUU | S→F |
| *R.rosea* | *nad5* | 29 | 1918 | 640 | 1 | C→U | CGU→UGU | R→C |
| *R.rosea* | *nad5* | 30 | 1958 | 653 | 2 | C→U | UCG→UUG | S→L |
| *R.rosea* | *nad5* | 31 | 2000 | 667 | 2 | C→U | UCA→UUA | S→L |
| *R.rosea* | *nad6* | 1 | 26 | 9 | 2 | C→U | CCU→CUU | P→L |
| *R.rosea* | *nad6* | 2 | 83 | 28 | 2 | C→U | UCG→UUG | S→L |
| *R.rosea* | *nad6* | 3 | 95 | 32 | 2 | C→U | CCU→CUU | P→L |
| *R.rosea* | *nad6* | 4 | 103 | 35 | 1 | C→U | CGC→UGC | R→C |
| *R.rosea* | *nad6* | 5 | 161 | 54 | 2 | C→U | CCA→CUA | P→L |
| *R.rosea* | *nad6* | 6 | 169 | 57 | 1 | C→U | CAU→UAU | H→Y |
| *R.rosea* | *nad6* | 7 | 191 | 64 | 2 | C→U | UCA→UUA | S→L |
| *R.rosea* | *nad6* | 8 | 463 | 155 | 1 | C→U | CCU→UCU | P→S |

| | | | | | | | | |
|---|---|---|---|---|---|---|---|---|
| *R.rosea* | *nad6* | 9 | 569 | 190 | 2 | C→U | UCU→UUU | S→F |
| *R.rosea* | *nad7* | 1 | 38 | 13 | 2 | C→U | UCG→UUG | S→L |
| *R.rosea* | *nad7* | 2 | 44 | 15 | 2 | C→U | UCC→UUC | S→F |
| *R.rosea* | *nad7* | 3 | 77 | 26 | 2 | C→U | UCA→UUA | S→L |
| *R.rosea* | *nad7* | 4 | 137 | 46 | 2 | C→U | UCA→UUA | S→L |
| *R.rosea* | *nad7* | 5 | 200 | 67 | 2 | C→U | UCU→UUU | S→F |
| *R.rosea* | *nad7* | 6 | 209 | 70 | 2 | C→U | UCA→UUA | S→L |
| *R.rosea* | *nad7* | 7 | 224 | 75 | 2 | C→U | ACG→AUG | T→M |
| *R.rosea* | *nad7* | 8 | 244 | 82 | 1 | C→U | CAU→UAU | H→Y |
| *R.rosea* | *nad7* | 9 | 251 | 84 | 2 | C→U | UCA→UUA | S→L |
| *R.rosea* | *nad7* | 10 | 316 | 106 | 1 | C→U | CGU→UGU | R→C |
| *R.rosea* | *nad7* | 11 | 335 | 112 | 2 | C→U | UCA→UUA | S→L |
| *R.rosea* | *nad7* | 12 | 344 | 115 | 2 | C→U | UCA→UUA | S→L |
| *R.rosea* | *nad7* | 13 | 383 | 128 | 2 | C→U | UCA→UUA | S→L |
| *R.rosea* | *nad7* | 14 | 445 | 149 | 1 | C→U | CCG→UCG | P→S |
| *R.rosea* | *nad7* | 15 | 531 | 177 | 3 | C→U | UCC→UCU | S→S |
| *R.rosea* | *nad7* | 16 | 533 | 178 | 2 | C→U | UCC→UUC | S→F |
| *R.rosea* | *nad7* | 17 | 578 | 193 | 2 | C→U | UCA→UUA | S→L |
| *R.rosea* | *nad7* | 18 | 679 | 227 | 1 | C→U | CCA→UCA | P→S |
| *R.rosea* | *nad7* | 19 | 724 | 242 | 1 | C→U | CAU→UAU | H→Y |
| *R.rosea* | *nad7* | 20 | 734 | 245 | 2 | C→U | UCG→UUG | S→L |
| *R.rosea* | *nad7* | 21 | 739 | 247 | 1 | C→U | CCU→UUU | P→F |
| *R.rosea* | *nad7* | | 740 | | 2 | C→U | | |
| *R.rosea* | *nad7* | 22 | 769 | 257 | 1 | C→U | CGC→UGC | R→C |
| *R.rosea* | *nad7* | 23 | 789 | 263 | 3 | C→U | AUC→AUU | I→I |
| *R.rosea* | *nad7* | 24 | 836 | 279 | 2 | C→U | CCU→CUU | P→L |
| *R.rosea* | *nad7* | 25 | 926 | 309 | 2 | C→U | UCA→UUA | S→L |
| *R.rosea* | *nad7* | 26 | 944 | 315 | 2 | C→U | CCU→CUU | P→L |
| *R.rosea* | *nad7* | 27 | 973 | 325 | 1 | C→U | CCU→UCU | P→S |
| *R.rosea* | *nad7* | 28 | 1050 | 350 | 3 | C→U | CCC→CCU | P→P |
| *R.rosea* | *nad7* | 29 | 1057 | 353 | 1 | C→U | CGU→UGU | R→C |
| *R.rosea* | *nad7* | 30 | 1079 | 360 | 2 | C→U | UCU→UUU | S→F |
| *R.rosea* | *nad7* | 31 | 1088 | 363 | 2 | C→U | UCA→UUA | S→L |
| *R.rosea* | *nad7* | 32 | 1103 | 368 | 2 | C→U | UCU→UUU | S→F |
| *R.rosea* | *nad7* | 33 | 1124 | 375 | 2 | C→U | CCA→CUA | P→L |
| *R.rosea* | *nad7* | 34 | 1166 | 389 | 2 | C→U | UCU→UUU | S→F |
| *R.rosea* | *nad9* | 1 | 14 | 5 | 2 | C→U | UCC→UUC | S→F |
| *R.rosea* | *nad9* | 2 | 92 | 31 | 2 | C→U | CCU→CUU | P→L |
| *R.rosea* | *nad9* | 3 | 113 | 38 | 2 | C→U | CCA→CUA | P→L |
| *R.rosea* | *nad9* | 4 | 167 | 56 | 2 | C→U | UCG→UUG | S→L |
| *R.rosea* | *nad9* | 5 | 190 | 64 | 1 | C→U | CAU→UAU | H→Y |
| *R.rosea* | *nad9* | 6 | 195 | 65 | 3 | C→U | CCC→CCU | P→P |
| *R.rosea* | *nad9* | 7 | 298 | 100 | 1 | C→U | CCG→UCG | P→S |
| *R.rosea* | *nad9* | 8 | 311 | 104 | 2 | C→U | CCA→CUA | P→L |
| *R.rosea* | *nad9* | 9 | 328 | 110 | 1 | C→U | CGG→UGG | R→W |
| *R.rosea* | *nad9* | 10 | 368 | 123 | 2 | C→U | UCC→UUC | S→F |
| *R.rosea* | *nad9* | 11 | 398 | 133 | 2 | C→U | UCA→UUA | S→L |
| *R.rosea* | *nad9* | 12 | 439 | 147 | 1 | C→U | CUU→UUU | L→F |
| *R.rosea* | *nad9* | 13 | 539 | 180 | 2 | C→U | UCU→UUU | S→F |

| | | | | | | | | |
|---|---|---|---|---|---|---|---|---|
| *R.rosea* | *rpl10* | 1 | 83 | 28 | 2 | C→U | UCA→UUA | S→L |
| *R.rosea* | *rpl10* | 2 | 101 | 34 | 2 | C→U | UCG→UUG | S→L |
| *R.rosea* | *rpl10* | 3 | 134 | 45 | 2 | C→U | CCA→CUA | P→L |
| *R.rosea* | *rpl10* | 4 | 155 | 52 | 2 | C→U | CCA→CUA | P→L |
| *R.rosea* | *rpl10* | 5 | 239 | 80 | 2 | C→U | UCG→UUG | S→L |
| *R.rosea* | *rpl10* | 6 | 314 | 105 | 2 | C→U | UCA→UUA | S→L |
| *R.rosea* | *rpl16* | 1 | 6 | 2 | 3 | C→U | CUC→CUU | L→L |
| *R.rosea* | *rpl16* | 2 | 37 | 13 | 1 | C→U | CAG→UAG | Q→* |
| *R.rosea* | *rpl16* | 3 | 183 | 61 | 3 | C→U | GGC→GGU | G→G |
| *R.rosea* | *rpl16* | 4 | 185 | 62 | 2 | C→U | ACU→AUU | T→I |
| *R.rosea* | *rpl16* | 5 | 313 | 105 | 1 | C→U | CUC→UUC | L→F |
| *R.rosea* | *rpl16* | 6 | 416 | 139 | 2 | C→U | CCA→CUA | P→L |
| *R.rosea* | *rpl16* | 7 | 482 | 161 | 2 | C→U | CCA→CUA | P→L |
| *R.rosea* | *rpl16* | 8 | 488 | 163 | 2 | C→U | UCG→UUG | S→L |
| *R.rosea* | *rpl5* | 1 | 35 | 12 | 2 | C→U | UCA→UUA | S→L |
| *R.rosea* | *rpl5* | 2 | 47 | 16 | 2 | C→U | CCG→CUG | P→L |
| *R.rosea* | *rpl5* | 3 | 59 | 20 | 2 | C→U | CCG→CUG | P→L |
| *R.rosea* | *rpl5* | 4 | 92 | 31 | 2 | C→U | UCG→UUG | S→L |
| *R.rosea* | *rpl5* | 5 | 166 | 56 | 1 | C→U | CCG→UCG | P→S |
| *R.rosea* | *rpl5* | 6 | 215 | 72 | 2 | C→U | UCG→UUG | S→L |
| *R.rosea* | *rpl5* | 7 | 317 | 106 | 2 | C→U | UCG→UUG | S→L |
| *R.rosea* | *rpl5* | 8 | 329 | 110 | 2 | C→U | UCG→UUG | S→L |
| *R.rosea* | *rpl5* | 9 | 512 | 171 | 2 | C→U | CCA→CUA | P→L |
| *R.rosea* | *rpl5* | 10 | 515 | 172 | 2 | C→U | CCG→CUG | P→L |
| *R.rosea* | *rps12* | 1 | 71 | 24 | 2 | C→U | UCG→UUG | S→L |
| *R.rosea* | *rps12* | 2 | 100 | 34 | 1 | C→U | CGC→UGC | R→C |
| *R.rosea* | *rps12* | 3 | 104 | 35 | 2 | C→U | CCA→CUA | P→L |
| *R.rosea* | *rps12* | 4 | 146 | 49 | 2 | C→U | CCC→CUC | P→L |
| *R.rosea* | *rps12* | 5 | 196 | 66 | 1 | C→U | CAC→UAC | H→Y |
| *R.rosea* | *rps12* | 6 | 221 | 74 | 2 | C→U | UCG→UUG | S→L |
| *R.rosea* | *rps12* | 7 | 284 | 95 | 2 | C→U | UCC→UUC | S→F |
| *R.rosea* | *rps13* | 1 | 56 | 19 | 2 | C→U | UCA→UUA | S→L |
| *R.rosea* | *rps13* | 2 | 100 | 34 | 1 | C→U | CGU→UGU | R→C |
| *R.rosea* | *rps13* | 3 | 287 | 96 | 2 | C→U | UCG→UUG | S→L |
| *R.rosea* | *rps7* | 1 | 116 | 39 | 2 | C→U | CCA→CUA | P→L |
| *R.rosea* | *rps7* | 2 | 332 | 111 | 2 | C→U | UCA→UUA | S→L |

Supplementary Table S3. RNA editing events identified in the mitochondrial PCGs of *R. crenulata.*

| Species | Gene | No. | Base | Aa | Triplet pos. | Bases | Codon | Aa change |
|---|---|---|---|---|---|---|---|---|
| *R.crenulata* | *atp1* | 1 | 1039 | 347 | 1 | C→U | CCC→UCC | P→S |
| *R.crenulata* | *atp1* | 2 | 1168 | 390 | 1 | C→U | CGC→UGC | R→C |
| *R.crenulata* | *atp1* | 3 | 1415 | 472 | 2 | C→U | CCA→CUA | P→L |
| *R.crenulata* | *atp1* | 4 | 1490 | 497 | 2 | C→U | CCA→CUA | P→L |
| *R.crenulata* | *atp4* | 1 | 15 | 5 | 3 | C→U | UCC→UCU | S→S |
| *R.crenulata* | *atp4* | 2 | 56 | 19 | 2 | C→U | CCA→CUA | P→L |
| *R.crenulata* | *atp4* | 3 | 59 | 20 | 2 | C→U | UCU→UUU | S→F |
| *R.crenulata* | *atp4* | 4 | 71 | 24 | 2 | C→U | UCA→UUA | S→L |
| *R.crenulata* | *atp4* | 5 | 89 | 30 | 2 | C→U | UCA→UUA | S→L |
| *R.crenulata* | *atp4* | 6 | 118 | 40 | 1 | C→U | CGU→UGU | R→C |
| *R.crenulata* | *atp4* | 7 | 138 | 46 | 3 | C→U | AUC→AUU | I→I |
| *R.crenulata* | *atp4* | 8 | 215 | 72 | 2 | C→U | UCG→UUG | S→L |
| *R.crenulata* | *atp4* | 9 | 227 | 76 | 2 | C→U | CCC→CUC | P→L |
| *R.crenulata* | *atp4* | 10 | 248 | 83 | 2 | C→U | CCU→CUU | P→L |
| *R.crenulata* | *atp4* | 11 | 251 | 84 | 2 | C→U | CCG→CUG | P→L |
| *R.crenulata* | *atp4* | 12 | 395 | 132 | 2 | C→U | UCA→UUA | S→L |
| *R.crenulata* | *atp4* | 13 | 407 | 136 | 2 | C→U | CCA→CUA | P→L |
| *R.crenulata* | *atp4* | 14 | 416 | 139 | 2 | C→U | ACU→AUU | T→I |
| *R.crenulata* | *atp6* | 1 | 68 | 23 | 2 | C→U | UCA→UUA | S→L |
| *R.crenulata* | *atp6* | 2 | 119 | 40 | 2 | C→U | CCG→CUG | P→L |
| *R.crenulata* | *atp6* | 3 | 181 | 61 | 1 | C→U | CGC→UGC | R→C |
| *R.crenulata* | *atp6* | 4 | 188 | 63 | 2 | C→U | UCG→UUG | S→L |
| *R.crenulata* | *atp6* | 5 | 214 | 72 | 1 | C→U | CGU→UGU | R→C |
| *R.crenulata* | *atp6* | 6 | 221 | 74 | 2 | C→U | CCC→CUC | P→L |
| *R.crenulata* | *atp6* | 7 | 353 | 118 | 2 | C→U | UCA→UUA | S→L |
| *R.crenulata* | *atp6* | 8 | 412 | 138 | 1 | C→U | CCU→UCU | P→S |
| *R.crenulata* | *atp6* | 9 | 415 | 139 | 1 | C→U | CAU→UAU | H→Y |
| *R.crenulata* | *atp6* | 10 | 437 | 146 | 2 | C→U | UCA→UUA | S→L |
| *R.crenulata* | *atp6* | 11 | 479 | 160 | 2 | C→U | UCA→UUA | S→L |
| *R.crenulata* | *atp6* | 12 | 551 | 184 | 2 | C→U | CCU→CUU | P→L |
| *R.crenulata* | *atp6* | 13 | 587 | 196 | 2 | C→U | CCG→CUG | P→L |
| *R.crenulata* | *atp6* | 14 | 608 | 203 | 2 | C→U | UCA→UUA | S→L |
| *R.crenulata* | *atp6* | 15 | 616 | 206 | 1 | C→U | CAU→UAU | H→Y |
| *R.crenulata* | *atp6* | 16 | 623 | 208 | 2 | C→U | UCU→UUU | S→F |
| *R.crenulata* | *atp6* | 17 | 632 | 211 | 2 | C→U | UCA→UUA | S→L |
| *R.crenulata* | *atp6* | 18 | 659 | 220 | 2 | C→U | ACA→AUA | T→I |
| *R.crenulata* | *atp6* | 19 | 670 | 224 | 1 | C→U | CAA→UAA | Q→* |
| *R.crenulata* | *atp8* | 1 | 47 | 16 | 2 | C→U | UCA→UUA | S→L |
| *R.crenulata* | *atp8* | 2 | 76 | 26 | 1 | C→U | CCC→UUC | P→F |
| *R.crenulata* | *atp8* | | 77 | | 2 | C→U | | |
| *R.crenulata* | *atp8* | 3 | 452 | 151 | 2 | C→U | CCA→CUA | P→L |
| *R.crenulata* | *ccmB* | 1 | 28 | 10 | 1 | C→U | CAU→UAU | H→Y |
| *R.crenulata* | *ccmB* | 2 | 43 | 15 | 1 | C→U | CCC→UCC | P→S |
| *R.crenulata* | *ccmB* | 3 | 71 | 24 | 2 | C→U | CCA→CUA | P→L |
| *R.crenulata* | *ccmB* | 4 | 80 | 27 | 2 | C→U | UCG→UUG | S→L |
| *R.crenulata* | *ccmB* | 5 | 149 | 50 | 2 | C→U | CCG→CUG | P→L |

| | | | | | | | | |
|---|---|---|---|---|---|---|---|---|
| *R.crenulata* | *ccmB* | 6 | 154 | 52 | 1 | C→U | CGG→UGG | R→W |
| *R.crenulata* | *ccmB* | 7 | 160 | 54 | 1 | C→U | CCU→UCU | P→S |
| *R.crenulata* | *ccmB* | 8 | 164 | 55 | 2 | C→U | CCG→CUG | P→L |
| *R.crenulata* | *ccmB* | 9 | 172 | 58 | 1 | C→U | CCU→UCU | P→S |
| *R.crenulata* | *ccmB* | 10 | 179 | 60 | 2 | C→U | CCU→CUU | P→L |
| *R.crenulata* | *ccmB* | 11 | 181 | 61 | 1 | C→U | CCC→UCC | P→S |
| *R.crenulata* | *ccmB* | 12 | 194 | 65 | 2 | C→U | CCU→CUU | P→L |
| *R.crenulata* | *ccmB* | 13 | 367 | 123 | 1 | C→U | CGG→UGG | R→W |
| *R.crenulata* | *ccmB* | 14 | 392 | 131 | 2 | C→U | CCG→CUG | P→L |
| *R.crenulata* | *ccmB* | 15 | 424 | 142 | 1 | C→U | CGU→UGU | R→C |
| *R.crenulata* | *ccmB* | 16 | 428 | 143 | 2 | C→U | UCG→UUG | S→L |
| *R.crenulata* | *ccmB* | 17 | 467 | 156 | 2 | C→U | UCG→UUG | S→L |
| *R.crenulata* | *ccmB* | 18 | 494 | 165 | 2 | C→U | UCA→UUA | S→L |
| *R.crenulata* | *ccmB* | 19 | 503 | 168 | 2 | C→U | CCA→CUA | P→L |
| *R.crenulata* | *ccmB* | 20 | 512 | 171 | 2 | C→U | UCU→UUU | S→F |
| *R.crenulata* | *ccmB* | 21 | 514 | 172 | 1 | C→U | CGU→UGU | R→C |
| *R.crenulata* | *ccmB* | 22 | 548 | 183 | 2 | C→U | CCU→CUU | P→L |
| *R.crenulata* | *ccmB* | 23 | 551 | 184 | 2 | C→U | UCA→UUA | S→L |
| *R.crenulata* | *ccmB* | 24 | 554 | 185 | 2 | C→U | UCG→UUG | S→L |
| *R.crenulata* | *ccmB* | 25 | 566 | 189 | 2 | C→U | UCU→UUU | S→F |
| *R.crenulata* | *ccmB* | 26 | 569 | 190 | 2 | C→U | UCU→UUU | S→F |
| *R.crenulata* | *ccmB* | 27 | 572 | 191 | 2 | C→U | CCG→CUG | P→L |
| *R.crenulata* | *ccmB* | 28 | 596 | 199 | 2 | C→U | UCG→UUG | S→L |
| *R.crenulata* | *ccmC* | 1 | 76 | 26 | 1 | C→U | CGG→UGG | R→W |
| *R.crenulata* | *ccmC* | 2 | 103 | 35 | 1 | C→U | CAU→UAU | H→Y |
| *R.crenulata* | *ccmC* | 3 | 115 | 39 | 1 | C→U | CGG→UGG | R→W |
| *R.crenulata* | *ccmC* | 4 | 133 | 45 | 1 | C→U | CUU→UUU | L→F |
| *R.crenulata* | *ccmC* | 5 | 161 | 54 | 2 | C→U | CCG→CUG | P→L |
| *R.crenulata* | *ccmC* | 6 | 184 | 62 | 1 | C→U | CGG→UGG | R→W |
| *R.crenulata* | *ccmC* | 7 | 281 | 94 | 2 | C→U | ACA→AUA | T→I |
| *R.crenulata* | *ccmC* | 8 | 299 | 100 | 2 | C→U | UCU→UUU | S→F |
| *R.crenulata* | *ccmC* | 9 | 331 | 111 | 1 | C→U | CGG→UGG | R→W |
| *R.crenulata* | *ccmC* | 10 | 358 | 120 | 1 | C→U | CGG→UGG | R→W |
| *R.crenulata* | *ccmC* | 11 | 395 | 132 | 2 | C→U | UCG→UUG | S→L |
| *R.crenulata* | *ccmC* | 12 | 399 | 133 | 3 | C→U | UUC→UUU | F→F |
| *R.crenulata* | *ccmC* | 13 | 400 | 134 | 1 | C→U | CUU→UUU | L→F |
| *R.crenulata* | *ccmC* | 14 | 418 | 140 | 1 | C→U | CUG→UUG | L→L |
| *R.crenulata* | *ccmC* | 15 | 436 | 146 | 1 | C→U | CCU→UCU | P→S |
| *R.crenulata* | *ccmC* | 16 | 446 | 149 | 2 | C→U | CCG→CUG | P→L |
| *R.crenulata* | *ccmC* | 17 | 451 | 151 | 1 | C→U | CCU→UCU | P→S |
| *R.crenulata* | *ccmC* | 18 | 458 | 153 | 2 | C→U | UCA→UUA | S→L |
| *R.crenulata* | *ccmC* | 19 | 463 | 155 | 1 | C→U | CGU→UGU | R→C |
| *R.crenulata* | *ccmC* | 20 | 467 | 156 | 2 | C→U | GCU→GUU | A→V |
| *R.crenulata* | *ccmC* | 21 | 473 | 158 | 2 | C→U | CCG→CUG | P→L |
| *R.crenulata* | *ccmC* | 22 | 521 | 174 | 2 | C→U | UCG→UUG | S→L |
| *R.crenulata* | *ccmC* | 23 | 568 | 190 | 1 | C→U | CCU→UCU | P→S |
| *R.crenulata* | *ccmC* | 24 | 575 | 192 | 2 | C→U | CCC→CUU | P→L |
| *R.crenulata* | *ccmC* | | 576 | | 3 | C→U | | |
| *R.crenulata* | *ccmC* | 25 | 605 | 202 | 2 | C→U | UCC→UUC | S→F |

| | | | | | | | | |
|---|---|---|---|---|---|---|---|---|
| *R.crenulata* | *ccmC* | 26 | 608 | 203 | 2 | C→U | CCC→CUC | P→L |
| *R.crenulata* | *ccmC* | 27 | 614 | 205 | 2 | C→U | UCA→UUA | S→L |
| *R.crenulata* | *ccmC* | 28 | 619 | 207 | 1 | C→U | CGU→UGU | R→C |
| *R.crenulata* | *ccmC* | 29 | 630 | 210 | 3 | C→U | CUC→CUU | L→L |
| *R.crenulata* | *ccmC* | 30 | 650 | 217 | 2 | C→U | CCU→CUU | P→L |
| *R.crenulata* | *ccmC* | 31 | 656 | 219 | 2 | C→U | CCA→CUA | P→L |
| *R.crenulata* | *ccmC* | 32 | 673 | 225 | 1 | C→U | CCC→UCC | P→S |
| *R.crenulata* | *ccmFc* | 1 | 38 | 13 | 2 | C→U | UCC→UUC | S→F |
| *R.crenulata* | *ccmFc* | 2 | 50 | 17 | 2 | C→U | CCU→CUU | P→L |
| *R.crenulata* | *ccmFc* | 3 | 52 | 18 | 1 | C→U | CGU→UGU | R→C |
| *R.crenulata* | *ccmFc* | 4 | 103 | 35 | 1 | C→U | CCC→UCC | P→S |
| *R.crenulata* | *ccmFc* | 5 | 119 | 40 | 2 | C→U | UCU→UUU | S→F |
| *R.crenulata* | *ccmFc* | 6 | 122 | 41 | 2 | C→U | UCC→UUC | S→F |
| *R.crenulata* | *ccmFc* | 7 | 146 | 49 | 2 | C→U | CCU→CUU | P→L |
| *R.crenulata* | *ccmFc* | 8 | 151 | 51 | 1 | C→U | CCU→UCU | P→S |
| *R.crenulata* | *ccmFc* | 9 | 296 | 99 | 2 | C→U | UCA→UUA | S→L |
| *R.crenulata* | *ccmFc* | 10 | 301 | 101 | 1 | C→U | CGU→UGU | R→C |
| *R.crenulata* | *ccmFc* | 11 | 382 | 128 | 1 | C→U | CGU→UGU | R→C |
| *R.crenulata* | *ccmFc* | 12 | 397 | 133 | 1 | C→U | CGU→UGU | R→C |
| *R.crenulata* | *ccmFc* | 13 | 878 | 293 | 2 | C→U | UCU→UUU | S→F |
| *R.crenulata* | *ccmFc* | 14 | 1139 | 380 | 2 | C→U | UCU→UUU | S→F |
| *R.crenulata* | *ccmFc* | 15 | 1160 | 387 | 2 | C→U | UCG→UUG | S→L |
| *R.crenulata* | *ccmFc* | 16 | 1234 | 412 | 1 | C→U | CGG→UGG | R→W |
| *R.crenulata* | *ccmFc* | 17 | 1259 | 420 | 2 | C→U | UCG→UUG | S→L |
| *R.crenulata* | *ccmFc* | 18 | 1268 | 423 | 2 | C→U | UCG→UUG | S→L |
| *R.crenulata* | *ccmFn* | 1 | 36 | 12 | 3 | C→U | UCC→UCU | S→S |
| *R.crenulata* | *ccmFn* | 2 | 96 | 32 | 3 | C→U | ACC→ACU | T→T |
| *R.crenulata* | *ccmFn* | 3 | 135 | 45 | 3 | C→U | UUC→UUU | F→F |
| *R.crenulata* | *ccmFn* | 4 | 140 | 47 | 2 | C→U | CCG→CUG | P→L |
| *R.crenulata* | *ccmFn* | 5 | 149 | 50 | 2 | C→U | UCC→UUC | S→F |
| *R.crenulata* | *ccmFn* | 6 | 246 | 82 | 3 | C→U | AUC→AUU | I→I |
| *R.crenulata* | *ccmFn* | 7 | 254 | 85 | 2 | C→U | UCG→UUG | S→L |
| *R.crenulata* | *ccmFn* | 8 | 261 | 87 | 3 | C→U | CCC→CCU | P→P |
| *R.crenulata* | *ccmFn* | 9 | 363 | 121 | 3 | C→U | CUC→CUU | L→L |
| *R.crenulata* | *ccmFn* | 10 | 370 | 124 | 1 | C→U | CGU→UGU | R→C |
| *R.crenulata* | *ccmFn* | 11 | 705 | 235 | 3 | C→U | UCC→UCU | S→S |
| *R.crenulata* | *ccmFn* | 12 | 714 | 238 | 3 | C→U | AUC→AUU | I→I |
| *R.crenulata* | *ccmFn* | 13 | 774 | 258 | 3 | C→U | AUC→AUU | I→I |
| *R.crenulata* | *ccmFn* | 14 | 786 | 262 | 3 | C→U | UCC→UCU | S→S |
| *R.crenulata* | *ccmFn* | 15 | 801 | 267 | 3 | C→U | AUC→AUU | I→I |
| *R.crenulata* | *ccmFn* | 16 | 944 | 315 | 2 | C→U | UCG→UUG | S→L |
| *R.crenulata* | *ccmFn* | 17 | 1179 | 393 | 3 | C→U | UUC→UUU | F→F |
| *R.crenulata* | *ccmFn* | 18 | 1262 | 421 | 2 | C→U | UCG→UUG | S→L |
| *R.crenulata* | *ccmFn* | 19 | 1290 | 430 | 3 | C→U | GCC→GCU | A→A |
| *R.crenulata* | *ccmFn* | 20 | 1307 | 436 | 2 | C→U | UCA→UUA | S→L |
| *R.crenulata* | *ccmFn* | 21 | 1322 | 441 | 2 | C→U | UCG→UUG | S→L |
| *R.crenulata* | *ccmFn* | 22 | 1340 | 447 | 2 | C→U | UCG→UUG | S→L |
| *R.crenulata* | *ccmFn* | 23 | 1373 | 458 | 2 | C→U | UCG→UUG | S→L |
| *R.crenulata* | *ccmFn* | 24 | 1458 | 486 | 3 | C→U | UCC→UCU | S→S |

| | | | | | | | | |
|---|---|---|---|---|---|---|---|---|
| *R.crenulata* | *ccmFn* | 25 | 1470 | 490 | 3 | C→U | CUC→CUU | L→L |
| *R.crenulata* | *ccmFn* | 26 | 1505 | 502 | 2 | C→U | UCC→UUC | S→F |
| *R.crenulata* | *cob* | 1 | 53 | 18 | 2 | C→U | ACA→AUA | T→I |
| *R.crenulata* | *cob* | 2 | 118 | 40 | 1 | C→U | CCG→UCG | P→S |
| *R.crenulata* | *cob* | 3 | 286 | 96 | 1 | C→U | CUC→UUC | L→F |
| *R.crenulata* | *cob* | 4 | 325 | 109 | 1 | C→U | CAU→UAU | H→Y |
| *R.crenulata* | *cob* | 5 | 358 | 120 | 1 | C→U | CGG→UGG | R→W |
| *R.crenulata* | *cob* | 6 | 407 | 136 | 2 | C→U | ACA→AUA | T→I |
| *R.crenulata* | *cob* | 7 | 419 | 140 | 2 | C→U | CCA→CUA | P→L |
| *R.crenulata* | *cob* | 8 | 568 | 190 | 1 | C→U | CAU→UAU | H→Y |
| *R.crenulata* | *cob* | 9 | 580 | 194 | 1 | C→U | CUU→UUU | L→F |
| *R.crenulata* | *cob* | 10 | 680 | 227 | 2 | C→U | UCU→UUU | S→F |
| *R.crenulata* | *cob* | 11 | 715 | 239 | 1 | C→U | CGG→UGG | R→W |
| *R.crenulata* | *cob* | 12 | 725 | 242 | 2 | C→U | UCU→UUU | S→F |
| *R.crenulata* | *cob* | 13 | 737 | 246 | 2 | C→U | UCU→UUU | S→F |
| *R.crenulata* | *cob* | 14 | 808 | 270 | 1 | C→U | CCC→UCC | P→S |
| *R.crenulata* | *cob* | 15 | 853 | 285 | 1 | C→U | CAU→UAU | H→Y |
| *R.crenulata* | *cob* | 16 | 908 | 303 | 2 | C→U | CCU→CUU | P→L |
| *R.crenulata* | *cob* | 17 | 982 | 328 | 1 | C→U | CAC→UAC | H→Y |
| *R.crenulata* | *cob* | 18 | 1084 | 362 | 1 | C→U | CCU→UCU | P→S |
| *R.crenulata* | *cox1* | 1 | 196 | 66 | 1 | C→U | CCU→UUU | P→F |
| *R.crenulata* | *cox1* | | 197 | | 2 | C→U | | |
| *R.crenulata* | *cox1* | 2 | 242 | 81 | 2 | C→U | UCU→UUU | S→F |
| *R.crenulata* | *cox1* | 3 | 254 | 85 | 2 | C→U | UCU→UUU | S→F |
| *R.crenulata* | *cox1* | 4 | 452 | 151 | 2 | C→U | UCU→UUU | S→F |
| *R.crenulata* | *cox1* | 5 | 515 | 172 | 2 | C→U | UCC→UUC | S→F |
| *R.crenulata* | *cox1* | 6 | 551 | 184 | 2 | C→U | UCA→UUA | S→L |
| *R.crenulata* | *cox1* | 7 | 590 | 197 | 2 | C→U | CCA→CUA | P→L |
| *R.crenulata* | *cox1* | 8 | 668 | 223 | 2 | C→U | UCU→UUU | S→F |
| *R.crenulata* | *cox1* | 9 | 715 | 239 | 1 | C→U | CGG→UGG | R→W |
| *R.crenulata* | *cox1* | 10 | 746 | 249 | 2 | C→U | CCC→CUC | P→L |
| *R.crenulata* | *cox1* | 11 | 860 | 287 | 2 | C→U | UCU→UUU | S→F |
| *R.crenulata* | *cox1* | 12 | 868 | 290 | 1 | C→U | CGG→UGG | R→W |
| *R.crenulata* | *cox1* | 13 | 1079 | 360 | 2 | C→U | CCG→CUG | P→L |
| *R.crenulata* | *cox1* | 14 | 1124 | 375 | 2 | C→U | GCG→GUG | A→V |
| *R.crenulata* | *cox1* | 15 | 1259 | 420 | 2 | C→U | UCU→UUU | S→F |
| *R.crenulata* | *cox1* | 16 | 1274 | 425 | 2 | C→U | CCG→CUG | P→L |
| *R.crenulata* | *cox1* | 17 | 1405 | 469 | 1 | C→U | CGU→UGU | R→C |
| *R.crenulata* | *cox1* | 18 | 1433 | 478 | 2 | C→U | UCA→UUA | S→L |
| *R.crenulata* | *cox1* | 19 | 1499 | 500 | 2 | C→U | CCG→CUG | P→L |
| *R.crenulata* | *cox2* | 1 | 11 | 4 | 2 | C→U | CCU→CUU | P→L |
| *R.crenulata* | *cox2* | 2 | 24 | 8 | 3 | C→U | UCC→UCU | S→S |
| *R.crenulata* | *cox2* | 3 | 57 | 19 | 3 | C→U | AUC→AUU | I→I |
| *R.crenulata* | *cox2* | 4 | 130 | 44 | 1 | C→U | CAU→UAU | H→Y |
| *R.crenulata* | *cox2* | 5 | 147 | 49 | 3 | C→U | CUC→CUU | L→L |
| *R.crenulata* | *cox2* | 6 | 149 | 50 | 2 | C→U | ACG→AUG | T→M |
| *R.crenulata* | *cox2* | 7 | 239 | 80 | 2 | C→U | UCG→UUG | S→L |
| *R.crenulata* | *cox2* | 8 | 264 | 88 | 3 | C→U | CCC→CCU | P→P |
| *R.crenulata* | *cox2* | 9 | 365 | 122 | 2 | C→U | UCG→UUG | S→L |

| | | | | | | | | |
|---|---|---|---|---|---|---|---|---|
| *R.crenulata* | *cox2* | 10 | 429 | 143 | 3 | C→U | UAC→UAU | Y→Y |
| *R.crenulata* | *cox2* | 11 | 447 | 149 | 3 | C→U | UCC→UCU | S→S |
| *R.crenulata* | *cox2* | 12 | 462 | 154 | 3 | C→U | AUC→AUU | I→I |
| *R.crenulata* | *cox2* | 13 | 567 | 189 | 3 | C→U | CUC→CUU | L→L |
| *R.crenulata* | *cox2* | 14 | 618 | 206 | 3 | C→U | UUC→UUU | F→F |
| *R.crenulata* | *cox2* | 15 | 684 | 228 | 3 | C→U | UAC→UAU | Y→Y |
| *R.crenulata* | *cox2* | 16 | 707 | 236 | 2 | C→U | UCC→UUC | S→F |
| *R.crenulata* | *cox2* | 17 | 728 | 243 | 2 | C→U | UCG→UUG | S→L |
| *R.crenulata* | *cox3* | 1 | 245 | 82 | 2 | C→U | CCU→CUU | P→L |
| *R.crenulata* | *cox3* | 2 | 257 | 86 | 2 | C→U | UCU→UUU | S→F |
| *R.crenulata* | *cox3* | 3 | 263 | 88 | 2 | C→U | CCG→CUG | P→L |
| *R.crenulata* | *cox3* | 4 | 289 | 97 | 1 | C→U | CUU→UUU | L→F |
| *R.crenulata* | *cox3* | 5 | 298 | 100 | 1 | C→U | CUU→UUU | L→F |
| *R.crenulata* | *cox3* | 6 | 304 | 102 | 1 | C→U | CGG→UGG | R→W |
| *R.crenulata* | *cox3* | 7 | 311 | 104 | 2 | C→U | UCU→UUU | S→F |
| *R.crenulata* | *cox3* | 8 | 314 | 105 | 2 | C→U | UCU→UUU | S→F |
| *R.crenulata* | *cox3* | 9 | 413 | 138 | 2 | C→U | CCU→CUU | P→L |
| *R.crenulata* | *cox3* | 10 | 422 | 141 | 2 | C→U | CCU→CUU | P→L |
| *R.crenulata* | *cox3* | 11 | 512 | 171 | 2 | C→U | UCA→UUA | S→L |
| *R.crenulata* | *cox3* | 12 | 527 | 176 | 2 | C→U | UCC→UUC | S→F |
| *R.crenulata* | *cox3* | 13 | 602 | 201 | 2 | C→U | UCC→UUC | S→F |
| *R.crenulata* | *cox3* | 14 | 653 | 218 | 2 | C→U | UCG→UUG | S→L |
| *R.crenulata* | *cox3* | 15 | 764 | 255 | 2 | C→U | CCA→CUA | P→L |
| *R.crenulata* | *matR* | 1 | 14 | 5 | 2 | C→U | UCC→UUC | S→F |
| *R.crenulata* | *matR* | 2 | 25 | 9 | 1 | C→U | CCC→UCC | P→S |
| *R.crenulata* | *matR* | 3 | 175 | 59 | 1 | C→U | CCA→UCA | P→S |
| *R.crenulata* | *matR* | 4 | 308 | 103 | 2 | C→U | CCG→CUG | P→L |
| *R.crenulata* | *matR* | 5 | 899 | 300 | 2 | C→U | UCA→UUA | S→L |
| *R.crenulata* | *matR* | 6 | 1295 | 432 | 2 | C→U | CCC→CUC | P→L |
| *R.crenulata* | *matR* | 7 | 1655 | 552 | 2 | C→U | UCC→UUC | S→F |
| *R.crenulata* | *matR* | 8 | 1696 | 566 | 1 | C→U | CGC→UGC | R→C |
| *R.crenulata* | *matR* | 9 | 1732 | 578 | 1 | C→U | CAC→UAC | H→Y |
| *R.crenulata* | *matR* | 10 | 1820 | 607 | 2 | C→U | UCU→UUU | S→F |
| *R.crenulata* | *mttB* | 1 | 259 | 87 | 1 | C→U | CUC→UUC | L→F |
| *R.crenulata* | *mttB* | 2 | 262 | 88 | 1 | C→U | CAU→UAU | H→Y |
| *R.crenulata* | *mttB* | 3 | 277 | 93 | 1 | C→U | CGC→UGC | R→C |
| *R.crenulata* | *mttB* | 4 | 284 | 95 | 2 | C→U | UCC→UUC | S→F |
| *R.crenulata* | *mttB* | 5 | 304 | 102 | 1 | C→U | CCU→UUU | P→F |
| *R.crenulata* | *mttB* | | 305 | | 2 | C→U | | |
| *R.crenulata* | *mttB* | 6 | 307 | 103 | 1 | C→U | CCA→UCA | P→S |
| *R.crenulata* | *mttB* | 7 | 310 | 104 | 1 | C→U | CGG→UGG | R→W |
| *R.crenulata* | *mttB* | 8 | 338 | 113 | 2 | C→U | CCA→CUA | P→L |
| *R.crenulata* | *mttB* | 9 | 368 | 123 | 2 | C→U | UCG→UUG | S→L |
| *R.crenulata* | *mttB* | 10 | 472 | 158 | 1 | C→U | CGU→UGU | R→C |
| *R.crenulata* | *mttB* | 11 | 479 | 160 | 2 | C→U | CCA→CUA | P→L |
| *R.crenulata* | *mttB* | 12 | 485 | 162 | 2 | C→U | CCA→CUA | P→L |
| *R.crenulata* | *mttB* | 13 | 541 | 181 | 1 | C→U | CCG→UCG | P→S |
| *R.crenulata* | *mttB* | 14 | 598 | 200 | 1 | C→U | CGU→UGU | R→C |
| *R.crenulata* | *mttB* | 15 | 611 | 204 | 2 | C→U | UCU→UUU | S→F |

| | | | | | | | | |
|---|---|---|---|---|---|---|---|---|
| *R.crenulata* | *mttB* | 16 | 635 | 212 | 2 | C→U | UCU→UUU | S→F |
| *R.crenulata* | *mttB* | 17 | 644 | 215 | 2 | C→U | UCG→UUG | S→L |
| *R.crenulata* | *nad1* | 1 | 167 | 56 | 2 | C→U | UCG→UUG | S→L |
| *R.crenulata* | *nad1* | 2 | 215 | 72 | 2 | C→U | UCC→UUC | S→F |
| *R.crenulata* | *nad1* | 3 | 265 | 89 | 1 | C→U | CGG→UGG | R→W |
| *R.crenulata* | *nad1* | 4 | 307 | 103 | 1 | C→U | CUG→UUG | L→L |
| *R.crenulata* | *nad1* | 5 | 376 | 126 | 1 | C→U | CGG→UGG | R→W |
| *R.crenulata* | *nad1* | 6 | 401 | 134 | 2 | C→U | UCU→UUU | S→F |
| *R.crenulata* | *nad1* | 7 | 436 | 146 | 1 | C→U | CCU→UCU | P→S |
| *R.crenulata* | *nad1* | 8 | 490 | 164 | 1 | C→U | CCC→UCU | P→S |
| *R.crenulata* | *nad1* | | 492 | | 3 | C→U | | |
| *R.crenulata* | *nad1* | 9 | 493 | 165 | 1 | C→U | CGU→UGU | R→C |
| *R.crenulata* | *nad1* | 10 | 500 | 167 | 2 | C→U | UCG→UUG | S→L |
| *R.crenulata* | *nad1* | 11 | 536 | 179 | 2 | C→U | UCC→UUC | S→F |
| *R.crenulata* | *nad1* | 12 | 571 | 191 | 1 | C→U | CUC→UUU | L→F |
| *R.crenulata* | *nad1* | | 573 | | 3 | C→U | | |
| *R.crenulata* | *nad1* | 13 | 580 | 194 | 1 | C→U | CGU→UGU | R→C |
| *R.crenulata* | *nad1* | 14 | 635 | 212 | 2 | C→U | UCA→UUA | S→L |
| *R.crenulata* | *nad1* | 15 | 683 | 228 | 2 | C→U | UCU→UUU | S→F |
| *R.crenulata* | *nad1* | 16 | 725 | 242 | 2 | C→U | CCA→CUA | P→L |
| *R.crenulata* | *nad1* | 17 | 743 | 248 | 2 | C→U | CCA→CUA | P→L |
| *R.crenulata* | *nad1* | 18 | 755 | 252 | 2 | C→U | CCG→CUG | P→L |
| *R.crenulata* | *nad1* | 19 | 779 | 260 | 2 | C→U | UCC→UUC | S→F |
| *R.crenulata* | *nad1* | 20 | 898 | 300 | 1 | C→U | CGG→UGG | R→W |
| *R.crenulata* | *nad1* | 21 | 928 | 310 | 1 | C→U | CGG→UGG | R→W |
| *R.crenulata* | *nad1* | 22 | 937 | 313 | 1 | C→U | CCC→UCC | P→S |
| *R.crenulata* | *nad1* | 23 | 953 | 318 | 2 | C→U | UCA→UUA | S→L |
| *R.crenulata* | *nad2* | 1 | 26 | 9 | 2 | C→U | UCC→UUC | S→F |
| *R.crenulata* | *nad2* | 2 | 223 | 75 | 1 | C→U | CUU→UUU | L→F |
| *R.crenulata* | *nad2* | 3 | 308 | 103 | 2 | C→U | UCU→UUU | S→F |
| *R.crenulata* | *nad2* | 4 | 311 | 104 | 2 | C→U | UCC→UUC | S→F |
| *R.crenulata* | *nad2* | 5 | 341 | 114 | 2 | C→U | UCC→UUC | S→F |
| *R.crenulata* | *nad2* | 6 | 356 | 119 | 2 | C→U | CCA→CUA | P→L |
| *R.crenulata* | *nad2* | 7 | 361 | 121 | 1 | C→U | CCU→UCU | P→S |
| *R.crenulata* | *nad2* | 8 | 367 | 123 | 1 | C→U | CGC→UGC | R→C |
| *R.crenulata* | *nad2* | 9 | 394 | 132 | 1 | C→U | CAU→UAU | H→Y |
| *R.crenulata* | *nad2* | 10 | 401 | 134 | 2 | C→U | UCA→UUA | S→L |
| *R.crenulata* | *nad2* | 11 | 428 | 143 | 2 | C→U | CCU→CUU | P→L |
| *R.crenulata* | *nad2* | 12 | 497 | 166 | 2 | C→U | UCG→UUG | S→L |
| *R.crenulata* | *nad2* | 13 | 523 | 175 | 1 | C→U | CCC→UCC | P→S |
| *R.crenulata* | *nad2* | 14 | 788 | 263 | 2 | C→U | UCU→UUU | S→F |
| *R.crenulata* | *nad2* | 15 | 800 | 267 | 2 | C→U | UCA→UUA | S→L |
| *R.crenulata* | *nad2* | 16 | 809 | 270 | 2 | C→U | UCU→UUU | S→F |
| *R.crenulata* | *nad2* | 17 | 920 | 307 | 2 | C→U | CCU→CUU | P→L |
| *R.crenulata* | *nad2* | 18 | 928 | 310 | 1 | C→U | CAU→UAU | H→Y |
| *R.crenulata* | *nad2* | 19 | 958 | 320 | 1 | C→U | CGU→UGU | R→C |
| *R.crenulata* | *nad2* | 20 | 962 | 321 | 2 | C→U | ACU→AUU | T→I |
| *R.crenulata* | *nad2* | 21 | 1028 | 343 | 2 | C→U | UCA→UUA | S→L |
| *R.crenulata* | *nad2* | 22 | 1058 | 353 | 2 | C→U | UCA→UUA | S→L |

| | | | | | | | | |
|---|---|---|---|---|---|---|---|---|
| *R.crenulata* | *nad2* | 23 | 1127 | 376 | 2 | C→U | UCG→UUG | S→L |
| *R.crenulata* | *nad2* | 24 | 1246 | 416 | 1 | C→U | CCA→UCA | P→S |
| *R.crenulata* | *nad2* | 25 | 1298 | 433 | 2 | C→U | GCG→GUG | A→V |
| *R.crenulata* | *nad2* | 26 | 1400 | 467 | 2 | C→U | UCA→UUA | S→L |
| *R.crenulata* | *nad2* | 27 | 1403 | 468 | 2 | C→U | UCC→UUC | S→F |
| *R.crenulata* | *nad2* | 28 | 1408 | 470 | 1 | C→U | CCA→UUA | P→L |
| *R.crenulata* | *nad2* | | 1409 | | 2 | C→U | | |
| *R.crenulata* | *nad2* | 29 | 1416 | 472 | 3 | C→U | CCC→CCU | P→P |
| *R.crenulata* | *nad2* | 30 | 1457 | 486 | 2 | C→U | UCA→UUA | S→L |
| *R.crenulata* | *nad3* | 1 | 5 | 2 | 2 | C→U | UCA→UUA | S→L |
| *R.crenulata* | *nad3* | 2 | 44 | 15 | 2 | C→U | CCG→CUG | P→L |
| *R.crenulata* | *nad3* | 3 | 62 | 21 | 2 | C→U | CCA→CUA | P→L |
| *R.crenulata* | *nad3* | 4 | 80 | 27 | 2 | C→U | CCA→CUA | P→L |
| *R.crenulata* | *nad3* | 5 | 124 | 42 | 1 | C→U | CAU→UAU | H→Y |
| *R.crenulata* | *nad3* | 6 | 137 | 46 | 2 | C→U | UCC→UUC | S→F |
| *R.crenulata* | *nad3* | 7 | 146 | 49 | 2 | C→U | UCC→UUC | S→F |
| *R.crenulata* | *nad3* | 8 | 208 | 70 | 1 | C→U | CCU→UUU | P→F |
| *R.crenulata* | *nad3* | | 209 | | 2 | C→U | | |
| *R.crenulata* | *nad3* | 9 | 215 | 72 | 2 | C→U | CCG→CUG | P→L |
| *R.crenulata* | *nad3* | 10 | 230 | 77 | 2 | C→U | UCC→UUC | S→F |
| *R.crenulata* | *nad3* | 11 | 247 | 83 | 1 | C→U | CCU→UCU | P→S |
| *R.crenulata* | *nad3* | 12 | 251 | 84 | 2 | C→U | CCC→CUC | P→L |
| *R.crenulata* | *nad3* | 13 | 266 | 89 | 2 | C→U | CCG→CUG | P→L |
| *R.crenulata* | *nad3* | 14 | 275 | 92 | 2 | C→U | UCU→UUU | S→F |
| *R.crenulata* | *nad3* | 15 | 317 | 106 | 2 | C→U | UCU→UUU | S→F |
| *R.crenulata* | *nad3* | 16 | 344 | 115 | 2 | C→U | UCG→UUG | S→L |
| *R.crenulata* | *nad3* | 17 | 349 | 117 | 1 | C→U | CGG→UGG | R→W |
| *R.crenulata* | *nad4* | 1 | 197 | 66 | 2 | C→U | UCU→UUU | S→F |
| *R.crenulata* | *nad4* | 2 | 317 | 106 | 2 | C→U | UCA→UUA | S→L |
| *R.crenulata* | *nad4* | 3 | 362 | 121 | 2 | C→U | GCA→GUA | A→V |
| *R.crenulata* | *nad4* | 4 | 368 | 123 | 2 | C→U | UCU→UUU | S→F |
| *R.crenulata* | *nad4* | 5 | 376 | 126 | 1 | C→U | CGU→UGU | R→C |
| *R.crenulata* | *nad4* | 6 | 403 | 135 | 1 | C→U | CGC→UGC | R→C |
| *R.crenulata* | *nad4* | 7 | 416 | 139 | 2 | C→U | CCU→CUU | P→L |
| *R.crenulata* | *nad4* | 8 | 433 | 145 | 1 | C→U | CUU→UUU | L→F |
| *R.crenulata* | *nad4* | 9 | 436 | 146 | 1 | C→U | CCC→UUC | P→F |
| *R.crenulata* | *nad4* | | 437 | | 2 | C→U | | |
| *R.crenulata* | *nad4* | 10 | 449 | 150 | 2 | C→U | CCA→CUA | P→L |
| *R.crenulata* | *nad4* | 11 | 608 | 203 | 2 | C→U | UCA→UUA | S→L |
| *R.crenulata* | *nad4* | 12 | 659 | 220 | 2 | C→U | UCU→UUU | S→F |
| *R.crenulata* | *nad4* | 13 | 767 | 256 | 2 | C→U | CCU→CUU | P→L |
| *R.crenulata* | *nad4* | 14 | 836 | 279 | 2 | C→U | UCC→UUC | S→F |
| *R.crenulata* | *nad4* | 15 | 839 | 280 | 2 | C→U | ACU→AUU | T→I |
| *R.crenulata* | *nad4* | 16 | 856 | 286 | 1 | C→U | CCA→UUA | P→L |
| *R.crenulata* | *nad4* | | 857 | | 2 | C→U | | |
| *R.crenulata* | *nad4* | 17 | 887 | 296 | 2 | C→U | UCG→UUG | S→L |
| *R.crenulata* | *nad4* | 18 | 896 | 299 | 2 | C→U | UCA→UUA | S→L |
| *R.crenulata* | *nad4* | 19 | 977 | 326 | 2 | C→U | CCG→CUG | P→L |
| *R.crenulata* | *nad4* | 20 | 1006 | 336 | 1 | C→U | CUA→UUA | L→L |

| | | | | | | | | |
|---|---|---|---|---|---|---|---|---|
| *R.crenulata* | *nad4* | 21 | 1010 | 337 | 2 | C→U | CCG→CUG | P→L |
| *R.crenulata* | *nad4* | 22 | 1016 | 339 | 2 | C→U | UCA→UUA | S→L |
| *R.crenulata* | *nad4* | 23 | 1033 | 345 | 1 | C→U | CCU→UCU | P→S |
| *R.crenulata* | *nad4* | 24 | 1129 | 377 | 1 | C→U | CUC→UUC | L→F |
| *R.crenulata* | *nad4* | 25 | 1142 | 381 | 2 | C→U | UCC→UUC | S→F |
| *R.crenulata* | *nad4* | 26 | 1148 | 383 | 2 | C→U | UCU→UUU | S→F |
| *R.crenulata* | *nad4* | 27 | 1151 | 384 | 2 | C→U | UCC→UUC | S→F |
| *R.crenulata* | *nad4* | 28 | 1172 | 391 | 2 | C→U | UCA→UUA | S→L |
| *R.crenulata* | *nad4* | 29 | 1307 | 436 | 2 | C→U | GCG→GUG | A→V |
| *R.crenulata* | *nad4* | 30 | 1355 | 452 | 2 | C→U | CCA→CUA | P→L |
| *R.crenulata* | *nad4* | 31 | 1373 | 458 | 2 | C→U | UCC→UUC | S→F |
| *R.crenulata* | *nad4* | 32 | 1405 | 469 | 1 | C→U | CGG→UGG | R→W |
| *R.crenulata* | *nad4* | 33 | 1417 | 473 | 1 | C→U | CAC→UAC | H→Y |
| *R.crenulata* | *nad4* | 34 | 1433 | 478 | 2 | C→U | CCG→CUG | P→L |
| *R.crenulata* | *nad4* | 35 | 1438 | 480 | 1 | C→U | CGC→UGC | R→C |
| *R.crenulata* | *nad4L* | 1 | 2 | 1 | 2 | C→U | ACG→AUG | T→M |
| *R.crenulata* | *nad4L* | 2 | 36 | 12 | 3 | C→U | AUC→AUU | I→I |
| *R.crenulata* | *nad4L* | 3 | 41 | 14 | 2 | C→U | UCU→UUU | S→F |
| *R.crenulata* | *nad4L* | 4 | 55 | 19 | 1 | C→U | CGG→UGG | R→W |
| *R.crenulata* | *nad4L* | 5 | 86 | 29 | 2 | C→U | CCU→CUU | P→L |
| *R.crenulata* | *nad4L* | 6 | 100 | 34 | 1 | C→U | CCA→UCA | P→S |
| *R.crenulata* | *nad4L* | 7 | 110 | 37 | 2 | C→U | UCA→UUA | S→L |
| *R.crenulata* | *nad4L* | 8 | 131 | 44 | 2 | C→U | UCG→UUG | S→L |
| *R.crenulata* | *nad4L* | 9 | 158 | 53 | 2 | C→U | UCG→UUG | S→L |
| *R.crenulata* | *nad4L* | 10 | 179 | 60 | 2 | C→U | UCA→UUA | S→L |
| *R.crenulata* | *nad4L* | 11 | 188 | 63 | 2 | C→U | UCA→UUA | S→L |
| *R.crenulata* | *nad4L* | 12 | 197 | 66 | 2 | C→U | CCA→CUA | P→L |
| *R.crenulata* | *nad4L* | 13 | 281 | 94 | 2 | C→U | UCU→UUU | S→F |
| *R.crenulata* | *nad5* | 1 | 155 | 52 | 2 | C→U | UCG→UUG | S→L |
| *R.crenulata* | *nad5* | 2 | 242 | 81 | 2 | C→U | CCG→CUG | P→L |
| *R.crenulata* | *nad5* | 3 | 272 | 91 | 2 | C→U | UCC→UUC | S→F |
| *R.crenulata* | *nad5* | 4 | 358 | 120 | 1 | C→U | CCU→UUU | P→F |
| *R.crenulata* | *nad5* | | 359 | | 2 | C→U | | |
| *R.crenulata* | *nad5* | 5 | 374 | 125 | 2 | C→U | CCA→CUA | P→L |
| *R.crenulata* | *nad5* | 6 | 398 | 133 | 2 | C→U | UCU→UUU | S→F |
| *R.crenulata* | *nad5* | 7 | 539 | 180 | 2 | C→U | CCU→CUU | P→L |
| *R.crenulata* | *nad5* | 8 | 548 | 183 | 2 | C→U | UCG→UUG | S→L |
| *R.crenulata* | *nad5* | 9 | 553 | 185 | 1 | C→U | CGU→UGU | R→C |
| *R.crenulata* | *nad5* | 10 | 598 | 200 | 1 | C→U | CGU→UGU | R→C |
| *R.crenulata* | *nad5* | 11 | 608 | 203 | 2 | C→U | GCC→GUC | A→V |
| *R.crenulata* | *nad5* | 12 | 629 | 210 | 2 | C→U | UCU→UUU | S→F |
| *R.crenulata* | *nad5* | 13 | 676 | 226 | 1 | C→U | CUU→UUU | L→F |
| *R.crenulata* | *nad5* | 14 | 713 | 238 | 2 | C→U | UCG→UUG | S→L |
| *R.crenulata* | *nad5* | 15 | 725 | 242 | 2 | C→U | UCA→UUA | S→L |
| *R.crenulata* | *nad5* | 16 | 835 | 279 | 1 | C→U | CCA→UCA | P→S |
| *R.crenulata* | *nad5* | 17 | 863 | 288 | 2 | C→U | UCU→UUU | S→F |
| *R.crenulata* | *nad5* | 18 | 875 | 292 | 2 | C→U | ACG→AUG | T→M |
| *R.crenulata* | *nad5* | 19 | 1184 | 395 | 2 | C→U | CCA→CUA | P→L |
| *R.crenulata* | *nad5* | 20 | 1310 | 437 | 2 | C→U | UCA→UUA | S→L |

| | | | | | | | | |
|---|---|---|---|---|---|---|---|---|
| *R.crenulata* | *nad5* | 21 | 1550 | 517 | 2 | C→U | ACC→AUC | T→I |
| *R.crenulata* | *nad5* | 22 | 1568 | 523 | 2 | C→U | CCG→CUG | P→L |
| *R.crenulata* | *nad5* | 23 | 1589 | 530 | 2 | C→U | UCU→UUU | S→F |
| *R.crenulata* | *nad5* | 24 | 1610 | 537 | 2 | C→U | CCC→CUC | P→L |
| *R.crenulata* | *nad5* | 25 | 1895 | 632 | 2 | C→U | UCA→UUA | S→L |
| *R.crenulata* | *nad5* | 26 | 1916 | 639 | 2 | C→U | UCU→UUU | S→F |
| *R.crenulata* | *nad5* | 27 | 1918 | 640 | 1 | C→U | CGU→UGU | R→C |
| *R.crenulata* | *nad5* | 28 | 1958 | 653 | 2 | C→U | UCG→UUG | S→L |
| *R.crenulata* | *nad5* | 29 | 2000 | 667 | 2 | C→U | UCA→UUA | S→L |
| *R.crenulata* | *nad6* | 1 | 26 | 9 | 2 | C→U | CCU→CUU | P→L |
| *R.crenulata* | *nad6* | 2 | 83 | 28 | 2 | C→U | UCG→UUG | S→L |
| *R.crenulata* | *nad6* | 3 | 95 | 32 | 2 | C→U | CCU→CUU | P→L |
| *R.crenulata* | *nad6* | 4 | 103 | 35 | 1 | C→U | CGC→UGC | R→C |
| *R.crenulata* | *nad6* | 5 | 161 | 54 | 2 | C→U | CCA→CUA | P→L |
| *R.crenulata* | *nad6* | 6 | 169 | 57 | 1 | C→U | CAU→UAU | H→Y |
| *R.crenulata* | *nad6* | 7 | 191 | 64 | 2 | C→U | UCA→UUA | S→L |
| *R.crenulata* | *nad6* | 8 | 463 | 155 | 1 | C→U | CCU→UCU | P→S |
| *R.crenulata* | *nad6* | 9 | 569 | 190 | 2 | C→U | UCU→UUU | S→F |
| *R.crenulata* | *nad7* | 1 | 38 | 13 | 2 | C→U | UCG→UUG | S→L |
| *R.crenulata* | *nad7* | 2 | 44 | 15 | 2 | C→U | UCC→UUC | S→F |
| *R.crenulata* | *nad7* | 3 | 77 | 26 | 2 | C→U | UCA→UUA | S→L |
| *R.crenulata* | *nad7* | 4 | 137 | 46 | 2 | C→U | UCA→UUA | S→L |
| *R.crenulata* | *nad7* | 5 | 200 | 67 | 2 | C→U | UCU→UUU | S→F |
| *R.crenulata* | *nad7* | 6 | 209 | 70 | 2 | C→U | UCA→UUA | S→L |
| *R.crenulata* | *nad7* | 7 | 224 | 75 | 2 | C→U | ACG→AUG | T→M |
| *R.crenulata* | *nad7* | 8 | 244 | 82 | 1 | C→U | CAU→UAU | H→Y |
| *R.crenulata* | *nad7* | 9 | 251 | 84 | 2 | C→U | UCA→UUA | S→L |
| *R.crenulata* | *nad7* | 10 | 316 | 106 | 1 | C→U | CGU→UGU | R→C |
| *R.crenulata* | *nad7* | 11 | 335 | 112 | 2 | C→U | UCA→UUA | S→L |
| *R.crenulata* | *nad7* | 12 | 344 | 115 | 2 | C→U | UCA→UUA | S→L |
| *R.crenulata* | *nad7* | 13 | 383 | 128 | 2 | C→U | UCA→UUA | S→L |
| *R.crenulata* | *nad7* | 14 | 445 | 149 | 1 | C→U | CCG→UCG | P→S |
| *R.crenulata* | *nad7* | 15 | 531 | 177 | 3 | C→U | UCC→UCU | S→S |
| *R.crenulata* | *nad7* | 16 | 533 | 178 | 2 | C→U | UCC→UUC | S→F |
| *R.crenulata* | *nad7* | 17 | 578 | 193 | 2 | C→U | UCA→UUA | S→L |
| *R.crenulata* | *nad7* | 18 | 679 | 227 | 1 | C→U | CCA→UCA | P→S |
| *R.crenulata* | *nad7* | 19 | 724 | 242 | 1 | C→U | CAU→UAU | H→Y |
| *R.crenulata* | *nad7* | 20 | 734 | 245 | 2 | C→U | UCG→UUG | S→L |
| *R.crenulata* | *nad7* | 21 | 739 | 247 | 1 | C→U | CCU→UUU | P→F |
| *R.crenulata* | *nad7* | | 740 | | 2 | C→U | | |
| *R.crenulata* | *nad7* | 22 | 769 | 257 | 1 | C→U | CGC→UGC | R→C |
| *R.crenulata* | *nad7* | 23 | 836 | 279 | 2 | C→U | CCU→CUU | P→L |
| *R.crenulata* | *nad7* | 24 | 926 | 309 | 2 | C→U | UCA→UUA | S→L |
| *R.crenulata* | *nad7* | 25 | 944 | 315 | 2 | C→U | CCU→CUU | P→L |
| *R.crenulata* | *nad7* | 26 | 973 | 325 | 1 | C→U | CCU→UCU | P→S |
| *R.crenulata* | *nad7* | 27 | 1050 | 350 | 3 | C→U | CCC→CCU | P→P |
| *R.crenulata* | *nad7* | 28 | 1057 | 353 | 1 | C→U | CGU→UGU | R→C |
| *R.crenulata* | *nad7* | 29 | 1079 | 360 | 2 | C→U | UCU→UUU | S→F |
| *R.crenulata* | *nad7* | 30 | 1088 | 363 | 2 | C→U | UCA→UUA | S→L |

| | | | | | | | | |
|---|---|---|---|---|---|---|---|---|
| *R.crenulata* | *nad7* | 31 | 1103 | 368 | 2 | C→U | UCU→UUU | S→F |
| *R.crenulata* | *nad7* | 32 | 1124 | 375 | 2 | C→U | CCA→CUA | P→L |
| *R.crenulata* | *nad7* | 33 | 1166 | 389 | 2 | C→U | UCU→UUU | S→F |
| *R.crenulata* | *nad9* | 1 | 29 | 10 | 2 | C→U | CCU→CUU | P→L |
| *R.crenulata* | *nad9* | 2 | 50 | 17 | 2 | C→U | CCA→CUA | P→L |
| *R.crenulata* | *nad9* | 3 | 104 | 35 | 2 | C→U | UCG→UUG | S→L |
| *R.crenulata* | *nad9* | 4 | 127 | 43 | 1 | C→U | CAU→UAU | H→Y |
| *R.crenulata* | *nad9* | 5 | 235 | 79 | 1 | C→U | CCG→UCG | P→S |
| *R.crenulata* | *nad9* | 6 | 248 | 83 | 2 | C→U | CCA→CUA | P→L |
| *R.crenulata* | *nad9* | 7 | 265 | 89 | 1 | C→U | CGG→UGG | R→W |
| *R.crenulata* | *nad9* | 8 | 305 | 102 | 2 | C→U | UCC→UUC | S→F |
| *R.crenulata* | *nad9* | 9 | 335 | 112 | 2 | C→U | UCA→UUA | S→L |
| *R.crenulata* | *nad9* | 10 | 376 | 126 | 1 | C→U | CUU→UUU | L→F |
| *R.crenulata* | *nad9* | 11 | 476 | 159 | 2 | C→U | UCU→UUU | S→F |
| *R.crenulata* | *rpl10* | 1 | 83 | 28 | 2 | C→U | UCA→UUA | S→L |
| *R.crenulata* | *rpl10* | 2 | 101 | 34 | 2 | C→U | UCG→UUG | S→L |
| *R.crenulata* | *rpl10* | 3 | 134 | 45 | 2 | C→U | CCA→CUA | P→L |
| *R.crenulata* | *rpl10* | 4 | 155 | 52 | 2 | C→U | CCA→CUA | P→L |
| *R.crenulata* | *rpl10* | 5 | 228 | 76 | 3 | C→U | CCC→CCU | P→P |
| *R.crenulata* | *rpl10* | 6 | 239 | 80 | 2 | C→U | UCG→UUG | S→L |
| *R.crenulata* | *rpl10* | 7 | 314 | 105 | 2 | C→U | UCA→UUA | S→L |
| *R.crenulata* | *rpl10* | 8 | 330 | 110 | 3 | C→U | UAC→UAU | Y→Y |
| *R.crenulata* | *rpl16* | 1 | 6 | 2 | 3 | C→U | CUC→CUU | L→L |
| *R.crenulata* | *rpl16* | 2 | 37 | 13 | 1 | C→U | CAG→UAG | Q→* |
| *R.crenulata* | *rpl16* | 3 | 185 | 62 | 2 | C→U | ACU→AUU | T→I |
| *R.crenulata* | *rpl16* | 4 | 313 | 105 | 1 | C→U | CUC→UUC | L→F |
| *R.crenulata* | *rpl16* | 5 | 416 | 139 | 2 | C→U | CCA→CUA | P→L |
| *R.crenulata* | *rpl16* | 6 | 482 | 161 | 2 | C→U | CCA→CUA | P→L |
| *R.crenulata* | *rpl16* | 7 | 488 | 163 | 2 | C→U | UCG→UUG | S→L |
| *R.crenulata* | *rpl5* | 1 | 35 | 12 | 2 | C→U | UCA→UUA | S→L |
| *R.crenulata* | *rpl5* | 2 | 47 | 16 | 2 | C→U | CCG→CUG | P→L |
| *R.crenulata* | *rpl5* | 3 | 59 | 20 | 2 | C→U | CCG→CUG | P→L |
| *R.crenulata* | *rpl5* | 4 | 64 | 22 | 1 | C→U | CAC→UAC | H→Y |
| *R.crenulata* | *rpl5* | 5 | 92 | 31 | 2 | C→U | UCG→UUG | S→L |
| *R.crenulata* | *rpl5* | 6 | 166 | 56 | 1 | C→U | CCG→UCG | P→S |
| *R.crenulata* | *rpl5* | 7 | 215 | 72 | 2 | C→U | UCG→UUG | S→L |
| *R.crenulata* | *rpl5* | 8 | 317 | 106 | 2 | C→U | UCG→UUG | S→L |
| *R.crenulata* | *rpl5* | 9 | 329 | 110 | 2 | C→U | UCG→UUG | S→L |
| *R.crenulata* | *rpl5* | 10 | 512 | 171 | 2 | C→U | CCA→CUA | P→L |
| *R.crenulata* | *rpl5* | 11 | 515 | 172 | 2 | C→U | CCG→CUG | P→L |
| *R.crenulata* | *rps12* | 1 | 71 | 24 | 2 | C→U | UCG→UUG | S→L |
| *R.crenulata* | *rps12* | 2 | 100 | 34 | 1 | C→U | CGC→UGC | R→C |
| *R.crenulata* | *rps12* | 3 | 104 | 35 | 2 | C→U | CCA→CUA | P→L |
| *R.crenulata* | *rps12* | 4 | 146 | 49 | 2 | C→U | CCC→CUC | P→L |
| *R.crenulata* | *rps12* | 5 | 196 | 66 | 1 | C→U | CAC→UAC | H→Y |
| *R.crenulata* | *rps12* | 6 | 284 | 95 | 2 | C→U | UCC→UUC | S→F |
| *R.crenulata* | *rps13* | 1 | 56 | 19 | 2 | C→U | UCA→UUA | S→L |
| *R.crenulata* | *rps13* | 2 | 100 | 34 | 1 | C→U | CGU→UGU | R→C |
| *R.crenulata* | *rps13* | 3 | 287 | 96 | 2 | C→U | UCG→UUG | S→L |

| | | | | | | | | |
|---|---|---|---|---|---|---|---|---|
| *R.crenulata* | *rps7* | 1 | 116 | 39 | 2 | C→U | CCA→CUA | P→L |
| *R.crenulata* | *rps7* | 2 | 332 | 111 | 2 | C→U | UCA→UUA | S→L |

Supplementary Table S4. RNA editing events identified in the mitochondrial PCGs of *R. sacra*.

| **Species** | **Gene** | **No.** | **Base** | **Aa** | **Triplet pos.** | **Bases** | **Codon** | **Aa change** |
|---|---|---|---|---|---|---|---|---|
| *R.sacra* | *atp1* | 1 | 1039 | 347 | 1 | C→U | CCC→UCC | P→S |
| *R.sacra* | *atp1* | 2 | 1168 | 390 | 1 | C→U | CGC→UGC | R→C |
| *R.sacra* | *atp1* | 3 | 1415 | 472 | 2 | C→U | CCA→CUA | P→L |
| *R.sacra* | *atp1* | 4 | 1490 | 497 | 2 | C→U | CCA→CUA | P→L |
| *R.sacra* | *atp4* | 1 | 56 | 19 | 2 | C→U | CCA→CUA | P→L |
| *R.sacra* | *atp4* | 2 | 59 | 20 | 2 | C→U | UCU→UUU | S→F |
| *R.sacra* | *atp4* | 3 | 71 | 24 | 2 | C→U | UCA→UUA | S→L |
| *R.sacra* | *atp4* | 4 | 89 | 30 | 2 | C→U | UCA→UUA | S→L |
| *R.sacra* | *atp4* | 5 | 118 | 40 | 1 | C→U | CGU→UGU | R→C |
| *R.sacra* | *atp4* | 6 | 138 | 46 | 3 | C→U | AUC→AUU | I→I |
| *R.sacra* | *atp4* | 7 | 215 | 72 | 2 | C→U | UCG→UUG | S→L |
| *R.sacra* | *atp4* | 8 | 227 | 76 | 2 | C→U | CCC→CUC | P→L |
| *R.sacra* | *atp4* | 9 | 248 | 83 | 2 | C→U | CCU→CUU | P→L |
| *R.sacra* | *atp4* | 10 | 251 | 84 | 2 | C→U | CCG→CUG | P→L |
| *R.sacra* | *atp4* | 11 | 395 | 132 | 2 | C→U | UCA→UUA | S→L |
| *R.sacra* | *atp4* | 12 | 407 | 136 | 2 | C→U | CCA→CUA | P→L |
| *R.sacra* | *atp4* | 13 | 416 | 139 | 2 | C→U | ACU→AUU | T→I |
| *R.sacra* | *atp6* | 1 | 68 | 23 | 2 | C→U | UCA→UUA | S→L |
| *R.sacra* | *atp6* | 2 | 119 | 40 | 2 | C→U | CCG→CUG | P→L |
| *R.sacra* | *atp6* | 3 | 181 | 61 | 1 | C→U | CGC→UGC | R→C |
| *R.sacra* | *atp6* | 4 | 188 | 63 | 2 | C→U | UCG→UUG | S→L |
| *R.sacra* | *atp6* | 5 | 214 | 72 | 1 | C→U | CGU→UGU | R→C |
| *R.sacra* | *atp6* | 6 | 221 | 74 | 2 | C→U | CCC→CUC | P→L |
| *R.sacra* | *atp6* | 7 | 353 | 118 | 2 | C→U | UCA→UUA | S→L |
| *R.sacra* | *atp6* | 8 | 412 | 138 | 1 | C→U | CCU→UCU | P→S |
| *R.sacra* | *atp6* | 9 | 415 | 139 | 1 | C→U | CAU→UAU | H→Y |
| *R.sacra* | *atp6* | 10 | 437 | 146 | 2 | C→U | UCA→UUA | S→L |
| *R.sacra* | *atp6* | 11 | 479 | 160 | 2 | C→U | UCA→UUA | S→L |
| *R.sacra* | *atp6* | 12 | 551 | 184 | 2 | C→U | CCU→CUU | P→L |
| *R.sacra* | *atp6* | 13 | 587 | 196 | 2 | C→U | CCG→CUG | P→L |
| *R.sacra* | *atp6* | 14 | 608 | 203 | 2 | C→U | UCA→UUA | S→L |
| *R.sacra* | *atp6* | 15 | 616 | 206 | 1 | C→U | CAU→UAU | H→Y |
| *R.sacra* | *atp6* | 16 | 623 | 208 | 2 | C→U | UCU→UUU | S→F |
| *R.sacra* | *atp6* | 17 | 632 | 211 | 2 | C→U | UCA→UUA | S→L |
| *R.sacra* | *atp6* | 18 | 659 | 220 | 2 | C→U | ACA→AUA | T→I |
| *R.sacra* | *atp6* | 19 | 670 | 224 | 1 | C→U | CAA→UAA | Q→* |
| *R.sacra* | *atp8* | 1 | 47 | 16 | 2 | C→U | UCA→UUA | S→L |
| *R.sacra* | *atp8* | 2 | 76 | 26 | 1 | C→U | CCC→UUC | P→F |
| *R.sacra* | *atp8* | | 77 | | 2 | C→U | | |
| *R.sacra* | *atp8* | 3 | 452 | 151 | 2 | C→U | CCA→CUA | P→L |
| *R.sacra* | *ccmB* | 1 | 28 | 10 | 1 | C→U | CAU→UAU | H→Y |

| | | | | | | | | |
|---|---|---|---|---|---|---|---|---|
| *R.sacra* | *ccmB* | 2 | 43 | 15 | 1 | C→U | CCC→UCC | P→S |
| *R.sacra* | *ccmB* | 3 | 71 | 24 | 2 | C→U | CCA→CUA | P→L |
| *R.sacra* | *ccmB* | 4 | 80 | 27 | 2 | C→U | UCG→UUG | S→L |
| *R.sacra* | *ccmB* | 5 | 128 | 43 | 2 | C→U | UCA→UUA | S→L |
| *R.sacra* | *ccmB* | 6 | 137 | 46 | 2 | C→U | UCC→UUC | S→F |
| *R.sacra* | *ccmB* | 7 | 149 | 50 | 2 | C→U | CCG→CUG | P→L |
| *R.sacra* | *ccmB* | 8 | 154 | 52 | 1 | C→U | CGG→UGG | R→W |
| *R.sacra* | *ccmB* | 9 | 160 | 54 | 1 | C→U | CCU→UCU | P→S |
| *R.sacra* | *ccmB* | 10 | 164 | 55 | 2 | C→U | CCG→CUG | P→L |
| *R.sacra* | *ccmB* | 11 | 172 | 58 | 1 | C→U | CCU→UCU | P→S |
| *R.sacra* | *ccmB* | 12 | 179 | 60 | 2 | C→U | CCU→CUU | P→L |
| *R.sacra* | *ccmB* | 13 | 181 | 61 | 1 | C→U | CCC→UCC | P→S |
| *R.sacra* | *ccmB* | 14 | 194 | 65 | 2 | C→U | CCU→CUU | P→L |
| *R.sacra* | *ccmB* | 15 | 286 | 96 | 1 | C→U | CGG→UGG | R→W |
| *R.sacra* | *ccmB* | 16 | 304 | 102 | 1 | C→U | CGU→UGU | R→C |
| *R.sacra* | *ccmB* | 17 | 313 | 105 | 1 | C→U | CGU→UGU | R→C |
| *R.sacra* | *ccmB* | 18 | 367 | 123 | 1 | C→U | CGG→UGG | R→W |
| *R.sacra* | *ccmB* | 19 | 392 | 131 | 2 | C→U | CCG→CUG | P→L |
| *R.sacra* | *ccmB* | 20 | 475 | 159 | 1 | C→U | CCA→UUA | P→L |
| *R.sacra* | *ccmB* | | 476 | | 2 | C→U | | |
| *R.sacra* | *ccmB* | 21 | 566 | 189 | 2 | C→U | UCU→UUU | S→F |
| *R.sacra* | *ccmB* | 22 | 569 | 190 | 2 | C→U | UCU→UUU | S→F |
| *R.sacra* | *ccmB* | 23 | 572 | 191 | 2 | C→U | CCG→CUG | P→L |
| *R.sacra* | *ccmB* | 24 | 596 | 199 | 2 | C→U | UCG→UUG | S→L |
| *R.sacra* | *ccmC* | 1 | 76 | 26 | 1 | C→U | CGG→UGG | R→W |
| *R.sacra* | *ccmC* | 2 | 103 | 35 | 1 | C→U | CAU→UAU | H→Y |
| *R.sacra* | *ccmC* | 3 | 115 | 39 | 1 | C→U | CGG→UGG | R→W |
| *R.sacra* | *ccmC* | 4 | 133 | 45 | 1 | C→U | CUU→UUU | L→F |
| *R.sacra* | *ccmC* | 5 | 161 | 54 | 2 | C→U | CCG→CUG | P→L |
| *R.sacra* | *ccmC* | 6 | 184 | 62 | 1 | C→U | CGG→UGG | R→W |
| *R.sacra* | *ccmC* | 7 | 281 | 94 | 2 | C→U | ACA→AUA | T→I |
| *R.sacra* | *ccmC* | 8 | 299 | 100 | 2 | C→U | UCU→UUU | S→F |
| *R.sacra* | *ccmC* | 9 | 331 | 111 | 1 | C→U | CGG→UGG | R→W |
| *R.sacra* | *ccmC* | 10 | 358 | 120 | 1 | C→U | CGG→UGG | R→W |
| *R.sacra* | *ccmC* | 11 | 395 | 132 | 2 | C→U | UCG→UUG | S→L |
| *R.sacra* | *ccmC* | 12 | 399 | 133 | 3 | C→U | UUC→UUU | F→F |
| *R.sacra* | *ccmC* | 13 | 400 | 134 | 1 | C→U | CUU→UUU | L→F |
| *R.sacra* | *ccmC* | 14 | 418 | 140 | 1 | C→U | CUG→UUG | L→L |
| *R.sacra* | *ccmC* | 15 | 436 | 146 | 1 | C→U | CCU→UCU | P→S |
| *R.sacra* | *ccmC* | 16 | 446 | 149 | 2 | C→U | CCG→CUG | P→L |
| *R.sacra* | *ccmC* | 17 | 451 | 151 | 1 | C→U | CCU→UCU | P→S |
| *R.sacra* | *ccmC* | 18 | 458 | 153 | 2 | C→U | UCA→UUA | S→L |
| *R.sacra* | *ccmC* | 19 | 463 | 155 | 1 | C→U | CGU→UGU | R→C |
| *R.sacra* | *ccmC* | 20 | 473 | 158 | 2 | C→U | CCG→CUG | P→L |
| *R.sacra* | *ccmC* | 21 | 521 | 174 | 2 | C→U | UCG→UUG | S→L |
| *R.sacra* | *ccmC* | 22 | 568 | 190 | 1 | C→U | CCU→UCU | P→S |
| *R.sacra* | *ccmC* | 23 | 575 | 192 | 2 | C→U | CCC→CUC | P→L |
| *R.sacra* | *ccmC* | 24 | 605 | 202 | 2 | C→U | UCC→UUC | S→F |
| *R.sacra* | *ccmC* | 25 | 608 | 203 | 2 | C→U | CCC→CUC | P→L |

| | | | | | | | | |
|---|---|---|---|---|---|---|---|---|
| *R.sacra* | *ccmC* | 26 | 614 | 205 | 2 | C→U | UCA→UUA | S→L |
| *R.sacra* | *ccmC* | 27 | 619 | 207 | 1 | C→U | CGU→UGU | R→C |
| *R.sacra* | *ccmC* | 28 | 624 | 208 | 3 | C→U | AUC→AUU | I→I |
| *R.sacra* | *ccmC* | 29 | 630 | 210 | 3 | C→U | CUC→CUU | L→L |
| *R.sacra* | *ccmC* | 30 | 650 | 217 | 2 | C→U | CCU→CUU | P→L |
| *R.sacra* | *ccmC* | 31 | 656 | 219 | 2 | C→U | CCA→CUA | P→L |
| *R.sacra* | *ccmC* | 32 | 673 | 225 | 1 | C→U | CCC→UCC | P→S |
| *R.sacra* | *ccmFc* | 1 | 38 | 13 | 2 | C→U | UCC→UUC | S→F |
| *R.sacra* | *ccmFc* | 2 | 50 | 17 | 2 | C→U | CCU→CUU | P→L |
| *R.sacra* | *ccmFc* | 3 | 52 | 18 | 1 | C→U | CGU→UGU | R→C |
| *R.sacra* | *ccmFc* | 4 | 103 | 35 | 1 | C→U | CCC→UCC | P→S |
| *R.sacra* | *ccmFc* | 5 | 119 | 40 | 2 | C→U | UCU→UUU | S→F |
| *R.sacra* | *ccmFc* | 6 | 122 | 41 | 2 | C→U | UCC→UUC | S→F |
| *R.sacra* | *ccmFc* | 7 | 146 | 49 | 2 | C→U | CCU→CUU | P→L |
| *R.sacra* | *ccmFc* | 8 | 151 | 51 | 1 | C→U | CCU→UCU | P→S |
| *R.sacra* | *ccmFc* | 9 | 160 | 54 | 1 | C→U | CCU→UCU | P→S |
| *R.sacra* | *ccmFc* | 10 | 296 | 99 | 2 | C→U | UCA→UUA | S→L |
| *R.sacra* | *ccmFc* | 11 | 301 | 101 | 1 | C→U | CGU→UGU | R→C |
| *R.sacra* | *ccmFc* | 12 | 382 | 128 | 1 | C→U | CGU→UGU | R→C |
| *R.sacra* | *ccmFc* | 13 | 397 | 133 | 1 | C→U | CGU→UGU | R→C |
| *R.sacra* | *ccmFc* | 14 | 878 | 293 | 2 | C→U | UCU→UUU | S→F |
| *R.sacra* | *ccmFc* | 15 | 1139 | 380 | 2 | C→U | UCU→UUU | S→F |
| *R.sacra* | *ccmFc* | 16 | 1160 | 387 | 2 | C→U | UCG→UUG | S→L |
| *R.sacra* | *ccmFc* | 17 | 1234 | 412 | 1 | C→U | CGG→UGG | R→W |
| *R.sacra* | *ccmFc* | 18 | 1259 | 420 | 2 | C→U | UCG→UUG | S→L |
| *R.sacra* | *ccmFc* | 19 | 1268 | 423 | 2 | C→U | UCG→UUG | S→L |
| *R.sacra* | *ccmFn* | 1 | 38 | 13 | 2 | C→U | CCG→CUG | P→L |
| *R.sacra* | *ccmFn* | 2 | 98 | 33 | 2 | C→U | CCC→CUC | P→L |
| *R.sacra* | *ccmFn* | 3 | 137 | 46 | 2 | C→U | UCG→UUG | S→L |
| *R.sacra* | *ccmFn* | 4 | 142 | 48 | 1 | C→U | CGU→UGU | R→C |
| *R.sacra* | *ccmFn* | 5 | 151 | 51 | 1 | C→U | CCU→UCU | P→S |
| *R.sacra* | *ccmFn* | 6 | 248 | 83 | 2 | C→U | UCA→UUA | S→L |
| *R.sacra* | *ccmFn* | 7 | 256 | 86 | 1 | C→U | CGG→UGG | R→W |
| *R.sacra* | *ccmFn* | 8 | 263 | 88 | 2 | C→U | CCA→CUA | P→L |
| *R.sacra* | *ccmFn* | 9 | 365 | 122 | 2 | C→U | UCG→UUG | S→L |
| *R.sacra* | *ccmFn* | 10 | 372 | 124 | 3 | C→U | UUC→UUU | F→F |
| *R.sacra* | *ccmFn* | 11 | 707 | 236 | 2 | C→U | CCU→CUU | P→L |
| *R.sacra* | *ccmFn* | 12 | 716 | 239 | 2 | C→U | UCA→UUA | S→L |
| *R.sacra* | *ccmFn* | 13 | 776 | 259 | 2 | C→U | UCA→UUA | S→L |
| *R.sacra* | *ccmFn* | 14 | 788 | 263 | 2 | C→U | CCA→CUA | P→L |
| *R.sacra* | *ccmFn* | 15 | 803 | 268 | 2 | C→U | UCA→UUA | S→L |
| *R.sacra* | *ccmFn* | 16 | 946 | 316 | 1 | C→U | CGC→UGC | R→C |
| *R.sacra* | *ccmFn* | 17 | 1181 | 394 | 2 | C→U | UCG→UUG | S→L |
| *R.sacra* | *ccmFn* | 18 | 1264 | 422 | 1 | C→U | CGG→UGG | R→W |
| *R.sacra* | *ccmFn* | 19 | 1292 | 431 | 2 | C→U | CCA→CUA | P→L |
| *R.sacra* | *ccmFn* | 20 | 1309 | 437 | 1 | C→U | CAU→UAU | H→Y |
| *R.sacra* | *ccmFn* | 21 | 1324 | 442 | 1 | C→U | CGG→UGG | R→W |
| *R.sacra* | *ccmFn* | 22 | 1342 | 448 | 1 | C→U | CGG→UGG | R→W |
| *R.sacra* | *ccmFn* | 23 | 1456 | 486 | 1 | C→U | CUU→UUU | L→F |

| | | | | | | | | |
|---|---|---|---|---|---|---|---|---|
| *R.sacra* | *ccmFn* | 24 | 1460 | 487 | 2 | C→U | CCA→CUA | P→L |
| *R.sacra* | *ccmFn* | 25 | 1472 | 491 | 2 | C→U | UCA→UUA | S→L |
| *R.sacra* | *ccmFn* | 26 | 1507 | 503 | 1 | C→U | CCC→UCC | P→S |
| *R.sacra* | *cob* | 1 | 118 | 40 | 1 | C→U | CCG→UCG | P→S |
| *R.sacra* | *cob* | 2 | 286 | 96 | 1 | C→U | CUC→UUC | L→F |
| *R.sacra* | *cob* | 3 | 325 | 109 | 1 | C→U | CAU→UAU | H→Y |
| *R.sacra* | *cob* | 4 | 358 | 120 | 1 | C→U | CGG→UGG | R→W |
| *R.sacra* | *cob* | 5 | 407 | 136 | 2 | C→U | ACA→AUA | T→I |
| *R.sacra* | *cob* | 6 | 419 | 140 | 2 | C→U | CCA→CUA | P→L |
| *R.sacra* | *cob* | 7 | 568 | 190 | 1 | C→U | CAU→UAU | H→Y |
| *R.sacra* | *cob* | 8 | 580 | 194 | 1 | C→U | CUU→UUU | L→F |
| *R.sacra* | *cob* | 9 | 680 | 227 | 2 | C→U | UCU→UUU | S→F |
| *R.sacra* | *cob* | 10 | 715 | 239 | 1 | C→U | CGG→UGG | R→W |
| *R.sacra* | *cob* | 11 | 725 | 242 | 2 | C→U | UCU→UUU | S→F |
| *R.sacra* | *cob* | 12 | 737 | 246 | 2 | C→U | UCU→UUU | S→F |
| *R.sacra* | *cob* | 13 | 808 | 270 | 1 | C→U | CCC→UCC | P→S |
| *R.sacra* | *cob* | 14 | 853 | 285 | 1 | C→U | CAU→UAU | H→Y |
| *R.sacra* | *cob* | 15 | 908 | 303 | 2 | C→U | CCU→CUU | P→L |
| *R.sacra* | *cob* | 16 | 982 | 328 | 1 | C→U | CAC→UAC | H→Y |
| *R.sacra* | *cob* | 17 | 1084 | 362 | 1 | C→U | CCU→UCU | P→S |
| *R.sacra* | *cox1* | 1 | 196 | 66 | 1 | C→U | CCU→UUU | P→F |
| *R.sacra* | *cox1* | | 197 | | 2 | C→U | | |
| *R.sacra* | *cox1* | 2 | 242 | 81 | 2 | C→U | UCU→UUU | S→F |
| *R.sacra* | *cox1* | 3 | 254 | 85 | 2 | C→U | UCU→UUU | S→F |
| *R.sacra* | *cox1* | 4 | 452 | 151 | 2 | C→U | UCU→UUU | S→F |
| *R.sacra* | *cox1* | 5 | 515 | 172 | 2 | C→U | UCC→UUC | S→F |
| *R.sacra* | *cox1* | 6 | 551 | 184 | 2 | C→U | UCA→UUA | S→L |
| *R.sacra* | *cox1* | 7 | 590 | 197 | 2 | C→U | CCA→CUA | P→L |
| *R.sacra* | *cox1* | 8 | 668 | 223 | 2 | C→U | UCU→UUU | S→F |
| *R.sacra* | *cox1* | 9 | 715 | 239 | 1 | C→U | CGG→UGG | R→W |
| *R.sacra* | *cox1* | 10 | 746 | 249 | 2 | C→U | CCC→CUC | P→L |
| *R.sacra* | *cox1* | 11 | 860 | 287 | 2 | C→U | UCU→UUU | S→F |
| *R.sacra* | *cox1* | 12 | 868 | 290 | 1 | C→U | CGG→UGG | R→W |
| *R.sacra* | *cox1* | 13 | 1079 | 360 | 2 | C→U | CCG→CUG | P→L |
| *R.sacra* | *cox1* | 14 | 1124 | 375 | 2 | C→U | GCG→GUG | A→V |
| *R.sacra* | *cox1* | 15 | 1259 | 420 | 2 | C→U | UCU→UUU | S→F |
| *R.sacra* | *cox1* | 16 | 1274 | 425 | 2 | C→U | CCG→CUG | P→L |
| *R.sacra* | *cox1* | 17 | 1405 | 469 | 1 | C→U | CGU→UGU | R→C |
| *R.sacra* | *cox1* | 18 | 1433 | 478 | 2 | C→U | UCA→UUA | S→L |
| *R.sacra* | *cox1* | 19 | 1499 | 500 | 2 | C→U | CCG→CUG | P→L |
| *R.sacra* | *cox2* | 1 | 11 | 4 | 2 | C→U | CCU→CUU | P→L |
| *R.sacra* | *cox2* | 2 | 24 | 8 | 3 | C→U | UCC→UCU | S→S |
| *R.sacra* | *cox2* | 3 | 57 | 19 | 3 | C→U | AUC→AUU | I→I |
| *R.sacra* | *cox2* | 4 | 130 | 44 | 1 | C→U | CAU→UAU | H→Y |
| *R.sacra* | *cox2* | 5 | 147 | 49 | 3 | C→U | CUC→CUU | L→L |
| *R.sacra* | *cox2* | 6 | 149 | 50 | 2 | C→U | ACG→AUG | T→M |
| *R.sacra* | *cox2* | 7 | 239 | 80 | 2 | C→U | UCG→UUG | S→L |
| *R.sacra* | *cox2* | 8 | 264 | 88 | 3 | C→U | CCC→CCU | P→P |
| *R.sacra* | *cox2* | 9 | 365 | 122 | 2 | C→U | UCG→UUG | S→L |

| | | | | | | | | |
|---|---|---|---|---|---|---|---|---|
| *R.sacra* | *cox2* | 10 | 429 | 143 | 3 | C→U | UAC→UAU | Y→Y |
| *R.sacra* | *cox2* | 11 | 447 | 149 | 3 | C→U | UCC→UCU | S→S |
| *R.sacra* | *cox2* | 12 | 462 | 154 | 3 | C→U | AUC→AUU | I→I |
| *R.sacra* | *cox2* | 13 | 567 | 189 | 3 | C→U | CUC→CUU | L→L |
| *R.sacra* | *cox2* | 14 | 618 | 206 | 3 | C→U | UUC→UUU | F→F |
| *R.sacra* | *cox2* | 15 | 684 | 228 | 3 | C→U | UAC→UAU | Y→Y |
| *R.sacra* | *cox2* | 16 | 707 | 236 | 2 | C→U | UCC→UUC | S→F |
| *R.sacra* | *cox2* | 17 | 728 | 243 | 2 | C→U | UCG→UUG | S→L |
| *R.sacra* | *cox3* | 1 | 245 | 82 | 2 | C→U | CCU→CUU | P→L |
| *R.sacra* | *cox3* | 2 | 257 | 86 | 2 | C→U | UCU→UUU | S→F |
| *R.sacra* | *cox3* | 3 | 263 | 88 | 2 | C→U | CCG→CUG | P→L |
| *R.sacra* | *cox3* | 4 | 289 | 97 | 1 | C→U | CUU→UUU | L→F |
| *R.sacra* | *cox3* | 5 | 298 | 100 | 1 | C→U | CUU→UUU | L→F |
| *R.sacra* | *cox3* | 6 | 304 | 102 | 1 | C→U | CGG→UGG | R→W |
| *R.sacra* | *cox3* | 7 | 311 | 104 | 2 | C→U | UCU→UUU | S→F |
| *R.sacra* | *cox3* | 8 | 314 | 105 | 2 | C→U | UCU→UUU | S→F |
| *R.sacra* | *cox3* | 9 | 413 | 138 | 2 | C→U | CCU→CUU | P→L |
| *R.sacra* | *cox3* | 10 | 422 | 141 | 2 | C→U | CCU→CUU | P→L |
| *R.sacra* | *cox3* | 11 | 512 | 171 | 2 | C→U | UCA→UUA | S→L |
| *R.sacra* | *cox3* | 12 | 527 | 176 | 2 | C→U | UCC→UUC | S→F |
| *R.sacra* | *cox3* | 13 | 602 | 201 | 2 | C→U | UCC→UUC | S→F |
| *R.sacra* | *cox3* | 14 | 653 | 218 | 2 | C→U | UCG→UUG | S→L |
| *R.sacra* | *cox3* | 15 | 764 | 255 | 2 | C→U | CCA→CUA | P→L |
| *R.sacra* | *matR* | 1 | 14 | 5 | 2 | C→U | UCC→UUC | S→F |
| *R.sacra* | *matR* | 2 | 25 | 9 | 1 | C→U | CCC→UCC | P→S |
| *R.sacra* | *matR* | 3 | 175 | 59 | 1 | C→U | CCA→UCA | P→S |
| *R.sacra* | *matR* | 4 | 308 | 103 | 2 | C→U | CCG→CUG | P→L |
| *R.sacra* | *matR* | 5 | 1802 | 601 | 2 | C→U | CCA→CUA | P→L |
| *R.sacra* | *matR* | 6 | 1820 | 607 | 2 | C→U | UCU→UUU | S→F |
| *R.sacra* | *mttB* | 1 | 43 | 15 | 1 | C→U | CCG→UCG | P→S |
| *R.sacra* | *mttB* | 2 | 193 | 65 | 1 | C→U | CAU→UAU | H→Y |
| *R.sacra* | *nad1* | 1 | 167 | 56 | 2 | C→U | UCG→UUG | S→L |
| *R.sacra* | *nad1* | 2 | 215 | 72 | 2 | C→U | UCC→UUC | S→F |
| *R.sacra* | *nad1* | 3 | 265 | 89 | 1 | C→U | CGG→UGG | R→W |
| *R.sacra* | *nad1* | 4 | 307 | 103 | 1 | C→U | CUG→UUG | L→L |
| *R.sacra* | *nad1* | 5 | 376 | 126 | 1 | C→U | CGG→UGG | R→W |
| *R.sacra* | *nad1* | 6 | 401 | 134 | 2 | C→U | UCU→UUU | S→F |
| *R.sacra* | *nad1* | 7 | 436 | 146 | 1 | C→U | CCU→UCU | P→S |
| *R.sacra* | *nad1* | 8 | 490 | 164 | 1 | C→U | CCC→UCU | P→S |
| *R.sacra* | *nad1* | | 492 | | 3 | C→U | | |
| *R.sacra* | *nad1* | 9 | 493 | 165 | 1 | C→U | CGU→UGU | R→C |
| *R.sacra* | *nad1* | 10 | 500 | 167 | 2 | C→U | UCG→UUG | S→L |
| *R.sacra* | *nad1* | 11 | 536 | 179 | 2 | C→U | UCC→UUC | S→F |
| *R.sacra* | *nad1* | 12 | 571 | 191 | 1 | C→U | CUC→UUU | L→F |
| *R.sacra* | *nad1* | | 573 | | 3 | C→U | | |
| *R.sacra* | *nad1* | 13 | 580 | 194 | 1 | C→U | CGU→UGU | R→C |
| *R.sacra* | *nad1* | 14 | 635 | 212 | 2 | C→U | UCA→UUA | S→L |
| *R.sacra* | *nad1* | 15 | 683 | 228 | 2 | C→U | UCU→UUU | S→F |
| *R.sacra* | *nad1* | 16 | 725 | 242 | 2 | C→U | CCA→CUA | P→L |

| | | | | | | | | |
|---|---|---|---|---|---|---|---|---|
| *R.sacra* | *nad1* | 17 | 743 | 248 | 2 | C→U | CCA→CUA | P→L |
| *R.sacra* | *nad1* | 18 | 755 | 252 | 2 | C→U | CCG→CUG | P→L |
| *R.sacra* | *nad1* | 19 | 779 | 260 | 2 | C→U | UCC→UUC | S→F |
| *R.sacra* | *nad1* | 20 | 898 | 300 | 1 | C→U | CGG→UGG | R→W |
| *R.sacra* | *nad1* | 21 | 909 | 303 | 3 | C→U | UUC→UUU | F→F |
| *R.sacra* | *nad1* | 22 | 928 | 310 | 1 | C→U | CGG→UGG | R→W |
| *R.sacra* | *nad1* | 23 | 937 | 313 | 1 | C→U | CCC→UCC | P→S |
| *R.sacra* | *nad1* | 24 | 953 | 318 | 2 | C→U | UCA→UUA | S→L |
| *R.sacra* | *nad2* | 1 | 26 | 9 | 2 | C→U | UCC→UUC | S→F |
| *R.sacra* | *nad2* | 2 | 223 | 75 | 1 | C→U | CUU→UUU | L→F |
| *R.sacra* | *nad2* | 3 | 308 | 103 | 2 | C→U | UCU→UUU | S→F |
| *R.sacra* | *nad2* | 4 | 311 | 104 | 2 | C→U | UCC→UUC | S→F |
| *R.sacra* | *nad2* | 5 | 341 | 114 | 2 | C→U | UCC→UUC | S→F |
| *R.sacra* | *nad2* | 6 | 356 | 119 | 2 | C→U | CCA→CUA | P→L |
| *R.sacra* | *nad2* | 7 | 361 | 121 | 1 | C→U | CCU→UCU | P→S |
| *R.sacra* | *nad2* | 8 | 367 | 123 | 1 | C→U | CGC→UGC | R→C |
| *R.sacra* | *nad2* | 9 | 394 | 132 | 1 | C→U | CAU→UAU | H→Y |
| *R.sacra* | *nad2* | 10 | 401 | 134 | 2 | C→U | UCA→UUA | S→L |
| *R.sacra* | *nad2* | 11 | 428 | 143 | 2 | C→U | CCU→CUU | P→L |
| *R.sacra* | *nad2* | 12 | 497 | 166 | 2 | C→U | UCG→UUG | S→L |
| *R.sacra* | *nad2* | 13 | 523 | 175 | 1 | C→U | CCC→UCC | P→S |
| *R.sacra* | *nad2* | 14 | 788 | 263 | 2 | C→U | UCU→UUU | S→F |
| *R.sacra* | *nad2* | 15 | 800 | 267 | 2 | C→U | UCA→UUA | S→L |
| *R.sacra* | *nad2* | 16 | 809 | 270 | 2 | C→U | UCU→UUU | S→F |
| *R.sacra* | *nad2* | 17 | 920 | 307 | 2 | C→U | CCU→CUU | P→L |
| *R.sacra* | *nad2* | 18 | 928 | 310 | 1 | C→U | CAU→UAU | H→Y |
| *R.sacra* | *nad2* | 19 | 958 | 320 | 1 | C→U | CGU→UGU | R→C |
| *R.sacra* | *nad2* | 20 | 962 | 321 | 2 | C→U | ACU→AUU | T→I |
| *R.sacra* | *nad2* | 21 | 1028 | 343 | 2 | C→U | UCA→UUA | S→L |
| *R.sacra* | *nad2* | 22 | 1058 | 353 | 2 | C→U | UCA→UUA | S→L |
| *R.sacra* | *nad2* | 23 | 1127 | 376 | 2 | C→U | UCG→UUG | S→L |
| *R.sacra* | *nad2* | 24 | 1246 | 416 | 1 | C→U | CCA→UCA | P→S |
| *R.sacra* | *nad2* | 25 | 1298 | 433 | 2 | C→U | GCG→GUG | A→V |
| *R.sacra* | *nad2* | 26 | 1400 | 467 | 2 | C→U | UCA→UUA | S→L |
| *R.sacra* | *nad2* | 27 | 1403 | 468 | 2 | C→U | UCC→UUC | S→F |
| *R.sacra* | *nad2* | 28 | 1408 | 470 | 1 | C→U | CCA→UUA | P→L |
| *R.sacra* | *nad2* | | 1409 | | 2 | C→U | | |
| *R.sacra* | *nad2* | 29 | 1416 | 472 | 3 | C→U | CCC→CCU | P→P |
| *R.sacra* | *nad2* | 30 | 1457 | 486 | 2 | C→U | UCA→UUA | S→L |
| *R.sacra* | *nad3* | 1 | 5 | 2 | 2 | C→U | UCA→UUA | S→L |
| *R.sacra* | *nad3* | 2 | 44 | 15 | 2 | C→U | CCG→CUG | P→L |
| *R.sacra* | *nad3* | 3 | 62 | 21 | 2 | C→U | CCA→CUA | P→L |
| *R.sacra* | *nad3* | 4 | 80 | 27 | 2 | C→U | CCA→CUA | P→L |
| *R.sacra* | *nad3* | 5 | 124 | 42 | 1 | C→U | CAU→UAU | H→Y |
| *R.sacra* | *nad3* | 6 | 137 | 46 | 2 | C→U | UCC→UUC | S→F |
| *R.sacra* | *nad3* | 7 | 146 | 49 | 2 | C→U | UCC→UUC | S→F |
| *R.sacra* | *nad3* | 8 | 208 | 70 | 1 | C→U | CCU→UUU | P→F |
| *R.sacra* | *nad3* | | 209 | | 2 | C→U | | |
| *R.sacra* | *nad3* | 9 | 215 | 72 | 2 | C→U | CCG→CUG | P→L |

| | | | | | | | | |
|---|---|---|---|---|---|---|---|---|
| *R.sacra* | *nad3* | 10 | 230 | 77 | 2 | C→U | UCC→UUC | S→F |
| *R.sacra* | *nad3* | 11 | 247 | 83 | 1 | C→U | CCU→UCU | P→S |
| *R.sacra* | *nad3* | 12 | 251 | 84 | 2 | C→U | CCC→CUC | P→L |
| *R.sacra* | *nad3* | 13 | 266 | 89 | 2 | C→U | CCG→CUG | P→L |
| *R.sacra* | *nad3* | 14 | 275 | 92 | 2 | C→U | UCU→UUU | S→F |
| *R.sacra* | *nad3* | 15 | 317 | 106 | 2 | C→U | UCU→UUU | S→F |
| *R.sacra* | *nad3* | 16 | 344 | 115 | 2 | C→U | UCG→UUG | S→L |
| *R.sacra* | *nad3* | 17 | 349 | 117 | 1 | C→U | CGG→UGG | R→W |
| *R.sacra* | *nad4* | 1 | 50 | 17 | 2 | C→U | CCG→CUG | P→L |
| *R.sacra* | *nad4* | 2 | 74 | 25 | 2 | C→U | ACU→AUU | T→I |
| *R.sacra* | *nad4* | 3 | 77 | 26 | 2 | C→U | CCU→CUU | P→L |
| *R.sacra* | *nad4* | 4 | 84 | 28 | 3 | C→U | UUC→UUU | F→F |
| *R.sacra* | *nad4* | 5 | 107 | 36 | 2 | C→U | CCG→CUG | P→L |
| *R.sacra* | *nad4* | 6 | 154 | 52 | 1 | C→U | CCC→UCC | P→S |
| *R.sacra* | *nad4* | 7 | 158 | 53 | 2 | C→U | CCU→CUU | P→L |
| *R.sacra* | *nad4* | 8 | 164 | 55 | 2 | C→U | CCU→CUU | P→L |
| *R.sacra* | *nad4* | 9 | 166 | 56 | 1 | C→U | CGG→UGG | R→W |
| *R.sacra* | *nad4* | 10 | 197 | 66 | 2 | C→U | UCU→UUU | S→F |
| *R.sacra* | *nad4* | 11 | 317 | 106 | 2 | C→U | UCA→UUA | S→L |
| *R.sacra* | *nad4* | 12 | 362 | 121 | 2 | C→U | GCA→GUA | A→V |
| *R.sacra* | *nad4* | 13 | 368 | 123 | 2 | C→U | UCU→UUU | S→F |
| *R.sacra* | *nad4* | 14 | 376 | 126 | 1 | C→U | CGU→UGU | R→C |
| *R.sacra* | *nad4* | 15 | 403 | 135 | 1 | C→U | CGC→UGC | R→C |
| *R.sacra* | *nad4* | 16 | 416 | 139 | 2 | C→U | CCU→CUU | P→L |
| *R.sacra* | *nad4* | 17 | 433 | 145 | 1 | C→U | CUU→UUU | L→F |
| *R.sacra* | *nad4* | 18 | 436 | 146 | 1 | C→U | CCC→UUC | P→F |
| *R.sacra* | *nad4* | | 437 | | 2 | C→U | | |
| *R.sacra* | *nad4* | 19 | 449 | 150 | 2 | C→U | CCA→CUA | P→L |
| *R.sacra* | *nad4* | 20 | 608 | 203 | 2 | C→U | UCA→UUA | S→L |
| *R.sacra* | *nad4* | 21 | 659 | 220 | 2 | C→U | UCU→UUU | S→F |
| *R.sacra* | *nad4* | 22 | 767 | 256 | 2 | C→U | CCU→CUU | P→L |
| *R.sacra* | *nad4* | 23 | 836 | 279 | 2 | C→U | UCC→UUC | S→F |
| *R.sacra* | *nad4* | 24 | 856 | 286 | 1 | C→U | CCA→UUA | P→L |
| *R.sacra* | *nad4* | | 857 | | 2 | C→U | | |
| *R.sacra* | *nad4* | 25 | 887 | 296 | 2 | C→U | UCG→UUG | S→L |
| *R.sacra* | *nad4* | 26 | 896 | 299 | 2 | C→U | UCA→UUA | S→L |
| *R.sacra* | *nad4* | 27 | 977 | 326 | 2 | C→U | CCG→CUG | P→L |
| *R.sacra* | *nad4* | 28 | 1006 | 336 | 1 | C→U | CUA→UUA | L→L |
| *R.sacra* | *nad4* | 29 | 1010 | 337 | 2 | C→U | CCG→CUG | P→L |
| *R.sacra* | *nad4* | 30 | 1016 | 339 | 2 | C→U | UCA→UUA | S→L |
| *R.sacra* | *nad4* | 31 | 1033 | 345 | 1 | C→U | CCU→UCU | P→S |
| *R.sacra* | *nad4* | 32 | 1129 | 377 | 1 | C→U | CUC→UUC | L→F |
| *R.sacra* | *nad4* | 33 | 1142 | 381 | 2 | C→U | UCC→UUC | S→F |
| *R.sacra* | *nad4* | 34 | 1148 | 383 | 2 | C→U | UCU→UUU | S→F |
| *R.sacra* | *nad4* | 35 | 1151 | 384 | 2 | C→U | UCC→UUC | S→F |
| *R.sacra* | *nad4* | 36 | 1172 | 391 | 2 | C→U | UCA→UUA | S→L |
| *R.sacra* | *nad4* | 37 | 1211 | 404 | 2 | C→U | UCA→UUA | S→L |
| *R.sacra* | *nad4* | 38 | 1307 | 436 | 2 | C→U | GCG→GUG | A→V |
| *R.sacra* | *nad4* | 39 | 1355 | 452 | 2 | C→U | CCA→CUA | P→L |

| | | | | | | | | |
|---|---|---|---|---|---|---|---|---|
| *R.sacra* | *nad4* | 40 | 1373 | 458 | 2 | C→U | UCC→UUC | S→F |
| *R.sacra* | *nad4* | 41 | 1405 | 469 | 1 | C→U | CGG→UGG | R→W |
| *R.sacra* | *nad4* | 42 | 1417 | 473 | 1 | C→U | CAC→UAC | H→Y |
| *R.sacra* | *nad4* | 43 | 1433 | 478 | 2 | C→U | CCG→CUG | P→L |
| *R.sacra* | *nad4* | 44 | 1438 | 480 | 1 | C→U | CGC→UGC | R→C |
| *R.sacra* | *nad4L* | 1 | 14 | 5 | 2 | C→U | UCC→UUC | S→F |
| *R.sacra* | *nad4L* | 2 | 92 | 31 | 2 | C→U | CCU→CUU | P→L |
| *R.sacra* | *nad4L* | 3 | 113 | 38 | 2 | C→U | CCA→CUA | P→L |
| *R.sacra* | *nad4L* | 4 | 118 | 40 | 1 | C→U | CCA→UCA | P→S |
| *R.sacra* | *nad4L* | 5 | 167 | 56 | 2 | C→U | UCG→UUG | S→L |
| *R.sacra* | *nad4L* | 6 | 190 | 64 | 1 | C→U | CAU→UAU | H→Y |
| *R.sacra* | *nad4L* | 7 | 298 | 100 | 1 | C→U | CCG→UCG | P→S |
| *R.sacra* | *nad4L* | 8 | 311 | 104 | 2 | C→U | CCA→CUA | P→L |
| *R.sacra* | *nad4L* | 9 | 328 | 110 | 1 | C→U | CGG→UGG | R→W |
| *R.sacra* | *nad4L* | 10 | 368 | 123 | 2 | C→U | UCC→UUC | S→F |
| *R.sacra* | *nad4L* | 11 | 398 | 133 | 2 | C→U | UCA→UUA | S→L |
| *R.sacra* | *nad4L* | 12 | 439 | 147 | 1 | C→U | CUU→UUU | L→F |
| *R.sacra* | *nad4L* | 13 | 539 | 180 | 2 | C→U | UCU→UUU | S→F |
| *R.sacra* | *nad5* | 1 | 110 | 37 | 2 | C→U | UCA→UUA | S→L |
| *R.sacra* | *nad5* | 2 | 155 | 52 | 2 | C→U | UCG→UUG | S→L |
| *R.sacra* | *nad5* | 3 | 242 | 81 | 2 | C→U | CCG→CUG | P→L |
| *R.sacra* | *nad5* | 4 | 272 | 91 | 2 | C→U | UCC→UUC | S→F |
| *R.sacra* | *nad5* | 5 | 315 | 105 | 3 | C→U | UCC→UCU | S→S |
| *R.sacra* | *nad5* | 6 | 358 | 120 | 1 | C→U | CCU→UUU | P→F |
| *R.sacra* | *nad5* | | 359 | | 2 | C→U | | |
| *R.sacra* | *nad5* | 7 | 374 | 125 | 2 | C→U | CCA→CUA | P→L |
| *R.sacra* | *nad5* | 8 | 398 | 133 | 2 | C→U | UCU→UUU | S→F |
| *R.sacra* | *nad5* | 9 | 539 | 180 | 2 | C→U | CCU→CUU | P→L |
| *R.sacra* | *nad5* | 10 | 548 | 183 | 2 | C→U | UCG→UUG | S→L |
| *R.sacra* | *nad5* | 11 | 553 | 185 | 1 | C→U | CGU→UGU | R→C |
| *R.sacra* | *nad5* | 12 | 598 | 200 | 1 | C→U | CGU→UGU | R→C |
| *R.sacra* | *nad5* | 13 | 676 | 226 | 1 | C→U | CUU→UUU | L→F |
| *R.sacra* | *nad5* | 14 | 713 | 238 | 2 | C→U | UCG→UUG | S→L |
| *R.sacra* | *nad5* | 15 | 725 | 242 | 2 | C→U | UCA→UUA | S→L |
| *R.sacra* | *nad5* | 16 | 835 | 279 | 1 | C→U | CCA→UCA | P→S |
| *R.sacra* | *nad5* | 17 | 863 | 288 | 2 | C→U | UCU→UUU | S→F |
| *R.sacra* | *nad5* | 18 | 875 | 292 | 2 | C→U | ACG→AUG | T→M |
| *R.sacra* | *nad5* | 19 | 1184 | 395 | 2 | C→U | CCA→CUA | P→L |
| *R.sacra* | *nad5* | 20 | 1310 | 437 | 2 | C→U | UCA→UUA | S→L |
| *R.sacra* | *nad5* | 21 | 1550 | 517 | 2 | C→U | ACC→AUC | T→I |
| *R.sacra* | *nad5* | 22 | 1568 | 523 | 2 | C→U | CCG→CUG | P→L |
| *R.sacra* | *nad5* | 23 | 1589 | 530 | 2 | C→U | UCU→UUU | S→F |
| *R.sacra* | *nad5* | 24 | 1610 | 537 | 2 | C→U | CCC→CUC | P→L |
| *R.sacra* | *nad5* | 25 | 1895 | 632 | 2 | C→U | UCA→UUA | S→L |
| *R.sacra* | *nad5* | 26 | 1916 | 639 | 2 | C→U | UCU→UUU | S→F |
| *R.sacra* | *nad5* | 27 | 1918 | 640 | 1 | C→U | CGU→UGU | R→C |
| *R.sacra* | *nad5* | 28 | 1958 | 653 | 2 | C→U | UCG→UUG | S→L |
| *R.sacra* | *nad5* | 29 | 2000 | 667 | 2 | C→U | UCA→UUA | S→L |
| *R.sacra* | *nad6* | 1 | 26 | 9 | 2 | C→U | CCU→CUU | P→L |

| | | | | | | | | |
|---|---|---|---|---|---|---|---|---|
| *R.sacra* | *nad6* | 2 | 83 | 28 | 2 | C→U | UCG→UUG | S→L |
| *R.sacra* | *nad6* | 3 | 95 | 32 | 2 | C→U | CCU→CUU | P→L |
| *R.sacra* | *nad6* | 4 | 103 | 35 | 1 | C→U | CGC→UGC | R→C |
| *R.sacra* | *nad6* | 5 | 161 | 54 | 2 | C→U | CCA→CUA | P→L |
| *R.sacra* | *nad6* | 6 | 169 | 57 | 1 | C→U | CAU→UAU | H→Y |
| *R.sacra* | *nad6* | 7 | 191 | 64 | 2 | C→U | UCA→UUA | S→L |
| *R.sacra* | *nad6* | 8 | 463 | 155 | 1 | C→U | CCU→UCU | P→S |
| *R.sacra* | *nad6* | 9 | 569 | 190 | 2 | C→U | UCU→UUU | S→F |
| *R.sacra* | *nad7* | 1 | 38 | 13 | 2 | C→U | UCG→UUG | S→L |
| *R.sacra* | *nad7* | 2 | 44 | 15 | 2 | C→U | UCC→UUC | S→F |
| *R.sacra* | *nad7* | 3 | 77 | 26 | 2 | C→U | UCA→UUA | S→L |
| *R.sacra* | *nad7* | 4 | 137 | 46 | 2 | C→U | UCA→UUA | S→L |
| *R.sacra* | *nad7* | 5 | 200 | 67 | 2 | C→U | UCU→UUU | S→F |
| *R.sacra* | *nad7* | 6 | 209 | 70 | 2 | C→U | UCA→UUA | S→L |
| *R.sacra* | *nad7* | 7 | 224 | 75 | 2 | C→U | ACG→AUG | T→M |
| *R.sacra* | *nad7* | 8 | 244 | 82 | 1 | C→U | CAU→UAU | H→Y |
| *R.sacra* | *nad7* | 9 | 251 | 84 | 2 | C→U | UCA→UUA | S→L |
| *R.sacra* | *nad7* | 10 | 316 | 106 | 1 | C→U | CGU→UGU | R→C |
| *R.sacra* | *nad7* | 11 | 335 | 112 | 2 | C→U | UCA→UUA | S→L |
| *R.sacra* | *nad7* | 12 | 344 | 115 | 2 | C→U | UCA→UUA | S→L |
| *R.sacra* | *nad7* | 13 | 383 | 128 | 2 | C→U | UCA→UUA | S→L |
| *R.sacra* | *nad7* | 14 | 445 | 149 | 1 | C→U | CCG→UCG | P→S |
| *R.sacra* | *nad7* | 15 | 531 | 177 | 3 | C→U | UCC→UCU | S→S |
| *R.sacra* | *nad7* | 16 | 533 | 178 | 2 | C→U | UCC→UUC | S→F |
| *R.sacra* | *nad7* | 17 | 578 | 193 | 2 | C→U | UCA→UUA | S→L |
| *R.sacra* | *nad7* | 18 | 679 | 227 | 1 | C→U | CCA→UCA | P→S |
| *R.sacra* | *nad7* | 19 | 724 | 242 | 1 | C→U | CAU→UAU | H→Y |
| *R.sacra* | *nad7* | 20 | 734 | 245 | 2 | C→U | UCG→UUG | S→L |
| *R.sacra* | *nad7* | 21 | 739 | 247 | 1 | C→U | CCU→UUU | P→F |
| *R.sacra* | *nad7* | | 740 | | 2 | C→U | | |
| *R.sacra* | *nad7* | 22 | 769 | 257 | 1 | C→U | CGC→UGC | R→C |
| *R.sacra* | *nad7* | 23 | 789 | 263 | 3 | C→U | AUC→AUU | I→I |
| *R.sacra* | *nad7* | 24 | 836 | 279 | 2 | C→U | CCU→CUU | P→L |
| *R.sacra* | *nad7* | 25 | 926 | 309 | 2 | C→U | UCA→UUA | S→L |
| *R.sacra* | *nad7* | 26 | 944 | 315 | 2 | C→U | CCU→CUU | P→L |
| *R.sacra* | *nad7* | 27 | 1057 | 353 | 1 | C→U | CGU→UGU | R→C |
| *R.sacra* | *nad7* | 28 | 1079 | 360 | 2 | C→U | UCU→UUU | S→F |
| *R.sacra* | *nad7* | 29 | 1088 | 363 | 2 | C→U | UCA→UUA | S→L |
| *R.sacra* | *nad7* | 30 | 1103 | 368 | 2 | C→U | UCU→UUU | S→F |
| *R.sacra* | *nad7* | 31 | 1124 | 375 | 2 | C→U | CCA→CUA | P→L |
| *R.sacra* | *nad7* | 32 | 1137 | 379 | 3 | C→U | GUC→GUU | V→V |
| *R.sacra* | *nad7* | 33 | 1166 | 389 | 2 | C→U | UCU→UUU | S→F |
| *R.sacra* | *nad9* | 1 | 14 | 5 | 2 | C→U | UCC→UUC | S→F |
| *R.sacra* | *nad9* | 2 | 92 | 31 | 2 | C→U | CCU→CUU | P→L |
| *R.sacra* | *nad9* | 3 | 113 | 38 | 2 | C→U | CCA→CUA | P→L |
| *R.sacra* | *nad9* | 4 | 118 | 40 | 1 | C→U | CCA→UCA | P→S |
| *R.sacra* | *nad9* | 5 | 167 | 56 | 2 | C→U | UCG→UUG | S→L |
| *R.sacra* | *nad9* | 6 | 190 | 64 | 1 | C→U | CAU→UAU | H→Y |
| *R.sacra* | *nad9* | 7 | 298 | 100 | 1 | C→U | CCG→UCG | P→S |

| | | | | | | | | |
|---|---|---|---|---|---|---|---|---|
| *R.sacra* | *nad9* | 8 | 311 | 104 | 2 | C→U | CCA→CUA | P→L |
| *R.sacra* | *nad9* | 9 | 328 | 110 | 1 | C→U | CGG→UGG | R→W |
| *R.sacra* | *nad9* | 10 | 368 | 123 | 2 | C→U | UCC→UUC | S→F |
| *R.sacra* | *nad9* | 11 | 398 | 133 | 2 | C→U | UCA→UUA | S→L |
| *R.sacra* | *nad9* | 12 | 439 | 147 | 1 | C→U | CUU→UUU | L→F |
| *R.sacra* | *nad9* | 13 | 539 | 180 | 2 | C→U | UCU→UUU | S→F |
| *R.sacra* | *rpl10* | 1 | 83 | 28 | 2 | C→U | UCA→UUA | S→L |
| *R.sacra* | *rpl10* | 2 | 101 | 34 | 2 | C→U | UCG→UUG | S→L |
| *R.sacra* | *rpl10* | 3 | 134 | 45 | 2 | C→U | CCA→CUA | P→L |
| *R.sacra* | *rpl10* | 4 | 155 | 52 | 2 | C→U | CCA→CUA | P→L |
| *R.sacra* | *rpl10* | 5 | 239 | 80 | 2 | C→U | UCG→UUG | S→L |
| *R.sacra* | *rpl10* | 6 | 314 | 105 | 2 | C→U | UCA→UUA | S→L |
| *R.sacra* | *rpl16* | 1 | 6 | 2 | 3 | C→U | CUC→CUU | L→L |
| *R.sacra* | *rpl16* | 2 | 37 | 13 | 1 | C→U | CAG→UAG | Q→* |
| *R.sacra* | *rpl16* | 3 | 185 | 62 | 2 | C→U | ACU→AUU | T→I |
| *R.sacra* | *rpl16* | 4 | 313 | 105 | 1 | C→U | CUC→UUC | L→F |
| *R.sacra* | *rpl16* | 5 | 416 | 139 | 2 | C→U | CCA→CUA | P→L |
| *R.sacra* | *rpl16* | 6 | 482 | 161 | 2 | C→U | CCA→CUA | P→L |
| *R.sacra* | *rpl16* | 7 | 488 | 163 | 2 | C→U | UCG→UUG | S→L |
| *R.sacra* | *rpl5* | 1 | 35 | 12 | 2 | C→U | UCA→UUA | S→L |
| *R.sacra* | *rpl5* | 2 | 47 | 16 | 2 | C→U | CCG→CUG | P→L |
| *R.sacra* | *rpl5* | 3 | 59 | 20 | 2 | C→U | CCG→CUG | P→L |
| *R.sacra* | *rpl5* | 4 | 64 | 22 | 1 | C→U | CAC→UAC | H→Y |
| *R.sacra* | *rpl5* | 5 | 92 | 31 | 2 | C→U | UCG→UUG | S→L |
| *R.sacra* | *rpl5* | 6 | 166 | 56 | 1 | C→U | CCG→UCG | P→S |
| *R.sacra* | *rpl5* | 7 | 317 | 106 | 2 | C→U | UCG→UUG | S→L |
| *R.sacra* | *rpl5* | 8 | 329 | 110 | 2 | C→U | UCG→UUG | S→L |
| *R.sacra* | *rpl5* | 9 | 512 | 171 | 2 | C→U | CCA→CUA | P→L |
| *R.sacra* | *rpl5* | 10 | 515 | 172 | 2 | C→U | CCG→CUG | P→L |
| *R.sacra* | *rps12* | 1 | 71 | 24 | 2 | C→U | UCG→UUG | S→L |
| *R.sacra* | *rps12* | 2 | 100 | 34 | 1 | C→U | CGC→UGC | R→C |
| *R.sacra* | *rps12* | 3 | 104 | 35 | 2 | C→U | CCA→CUA | P→L |
| *R.sacra* | *rps12* | 4 | 196 | 66 | 1 | C→U | CAC→UAC | H→Y |
| *R.sacra* | *rps12* | 5 | 221 | 74 | 2 | C→U | UCG→UUG | S→L |
| *R.sacra* | *rps12* | 6 | 284 | 95 | 2 | C→U | UCC→UUC | S→F |
| *R.sacra* | *rps13* | 1 | 56 | 19 | 2 | C→U | UCA→UUA | S→L |
| *R.sacra* | *rps13* | 2 | 100 | 34 | 1 | C→U | CGU→UGU | R→C |
| *R.sacra* | *rps13* | 3 | 287 | 96 | 2 | C→U | UCG→UUG | S→L |
| *R.sacra* | *rps13* | 4 | 310 | 104 | 1 | C→U | CAU→UAU | H→Y |
| *R.sacra* | *rps7* | 1 | 116 | 39 | 2 | C→U | CCA→CUA | P→L |
| *R.sacra* | *rps7* | 2 | 332 | 111 | 2 | C→U | UCA→UUA | S→L |

Supplementary Table S5. Statistics of assembly or annotation errors and ommisions in the mitochondrial genomes of three *Rhodiola* species on NCBI.

| Species | Gene | Exon | Assembly omission | Annotation omission | Annotation error |
|---|---|---|---|---|---|
| *R. crenulata* | *cox2* | exon1 | | | |
| *R. crenulata* | *cox2* | exon2 | | | |
| *R. crenulata* | *cox2* | exon3 | | | |
| *R. crenulata* | *ccmFc* | exon1 | | | end |
| *R. crenulata* | *ccmFc* | exon2 | | | start |
| *R. crenulata* | *nad1* | exon1 | √ | | |
| *R. crenulata* | *nad1* | exon2 | | √ | |
| *R. crenulata* | *nad1* | exon3 | | √ | |
| *R. crenulata* | *nad1* | exon4 | | √ | |
| *R. crenulata* | *nad1* | exon5 | | √ | |
| *R. crenulata* | *nad2* | exon1 | √ | | |
| *R. crenulata* | *nad2* | exon2 | √ | | |
| *R. crenulata* | *nad2* | exon3 | | | end |
| *R. crenulata* | *nad2* | exon4 | | | start, end |
| *R. crenulata* | *nad2* | exon5 | | | start |
| *R. crenulata* | *nad4* | exon1 | | | |
| *R. crenulata* | *nad4* | exon2 | | | |
| *R. crenulata* | *nad4* | exon3 | | | |
| *R. crenulata* | *nad4* | exon4 | | | |
| *R. crenulata* | *nad5* | exon1 | | | end |
| *R. crenulata* | *nad5* | exon2 | | | start |
| *R. crenulata* | *nad5* | exon3 | | √ | |
| *R. crenulata* | *nad5* | exon4 | | | start |
| *R. crenulata* | *nad5* | exon5 | | | |
| *R. crenulata* | *nad7* | exon1 | | | |
| *R. crenulata* | *nad7* | exon2 | | | |
| *R. crenulata* | *nad7* | exon3 | | | |
| *R. crenulata* | *nad7* | exon4 | | | |
| *R. crenulata* | *nad7* | exon5 | | | |
| *R. sacra* | *cox2* | exon1 | | | |
| *R. sacra* | *cox2* | exon2 | | | |
| *R. sacra* | *cox2* | exon3 | | | |
| *R. sacra* | *ccmFc* | exon1 | | | end |
| *R. sacra* | *ccmFc* | exon2 | | | start |
| *R. sacra* | *nad1* | exon1 | √ | | |
| *R. sacra* | *nad1* | exon2 | √ | | |
| *R. sacra* | *nad1* | exon3 | √ | | |
| *R. sacra* | *nad1* | exon4 | | √ | |
| *R. sacra* | *nad1* | exon5 | √ | | |
| *R. sacra* | *nad2* | exon1 | √ | | |
| *R. sacra* | *nad2* | exon2 | √ | | |
| *R. sacra* | *nad2* | exon3 | | | end |
| *R. sacra* | *nad2* | exon4 | | | start, end |
| *R. sacra* | *nad2* | exon5 | | | start |

| | | | | | |
|---|---|---|---|---|---|
| *R. sacra* | *nad4* | exon1 | | | |
| *R. sacra* | *nad4* | exon2 | | | |
| *R. sacra* | *nad4* | exon3 | | | end |
| *R. sacra* | *nad4* | exon4 | | | start |
| *R. sacra* | *nad5* | exon1 | √ | | |
| *R. sacra* | *nad5* | exon2 | √ | | |
| *R. sacra* | *nad5* | exon3 | √ | | |
| *R. sacra* | *nad5* | exon4 | | √ | |
| *R. sacra* | *nad5* | exon5 | | √ | |
| *R. sacra* | *nad7* | exon1 | | | |
| *R. sacra* | *nad7* | exon2 | | | |
| *R. sacra* | *nad7* | exon3 | | | |
| *R. sacra* | *nad7* | exon4 | | | |
| *R. sacra* | *nad7* | exon5 | | | |

Supplementary Table S6. Statistics of intron lengths of multi-exon mitochondrial genes of *Rhodiola* species on NCBI and the intron length thresholds set in RhoMitoAnnotator.

| **Gene** | **exon_num** | **organism** | **joinIntronCount (bp)** | **complementIntronCount (bp)** | **threasholdForAnno (bp)** |
|---|---|---|---|---|---|
| *ccmFc* | 2 | *R. rosea* | 938 | | |
| *ccmFc* | 2 | *R. crenulata* | 932 | | |
| *ccmFc* | 2 | *R. juparensis* | | 932 | 2000 |
| *ccmFc* | 2 | *R. tangutica* | 938 | | |
| *ccmFc* | 2 | *R. wallichiana* | 932 | | |
| *cox2* | 3 | *R. juparensis* | | 1393;1341 | |
| *cox2* | 3 | *R. rosea* | -102827;1400 | | 3500 |
| *cox2* | 3 | *R. tangutica* | 3051 | | |
| *nad1* | 5 | *R. rosea* | 901;11631 | 103535 | 2000 |
| *nad1* | 5 | *R. tangutica* | 63111;901;-134829 | | |
| *nad2* | 5 | *R. crenulata* | 1320;1501 | | |
| *nad2* | 5 | *R. juparensis* | 1325;1501 | | |
| *nad2* | 5 | *R. rosea* | 1022 | -1320;-1505 | 2000 |
| *nad2* | 5 | *R. sacra* | 1320;1497 | | |
| *nad2* | 5 | *R. tangutica* | 1022 | -1325;-1504 | |
| *nad4* | 4 | *R. crenulata* | 1418;2971;1984 | | |
| *nad4* | 4 | *R. juparensis* | 1417;2970;1984 | | 3500 |
| *nad4* | 4 | *R. rosea* | 1417;2971;1991 | | |
| *nad4* | 4 | *R. tangutica* | | 1981;2970;1418 | |
| *nad5* | 5 | *R. crenulata* | 853 | -931 | |
| *nad5* | 5 | *R. rosea* | -95847;931 | -853 | 2000 |
| *nad5* | 5 | *R. tangutica* | -102692;931 | -853 | |
| *nad7* | 5 | *R. crenulata* | | 1640;980;739;916 | |
| *nad7* | 5 | *R. juparensis* | | 1640;983;739;916 | 2000 |
| *nad7* | 5 | *R. rosea* | 914;739;980;1640 | | |

| | | | |
|---|---|---|---|
| *nad7* | 5 | *R. sacra* | 916;739;961;1639 |
| *nad7* | 5 | *R. tangutica* | 916;739;983;1640 |

Supplementary Table S7. Characteristics of non-canonical start and stop codons in *Rhodiola* mitogenomes.

| **Species** | **Genes** | **start codon (Non canonical)** | **stop codon (Non canonical)** |
|---|---|---|---|
| *R. juparensis* | *atp8* | ACG（RNA editing, C to U） | |
| *Rhodiola spp.* | *nad1* | ACG（RNA editing, C to U） | |
| *Rhodiola spp.* | *nad4L* | ACG（RNA editing, C to U） | |
| *Rhodiola spp.* | *atp6* | | CAA（RNA editing, C to U） |
| *Rhodiola spp.* | *cob* | ATT | |
| *R. tangutica* | *atp8* | GTG | |
| *R. crenulata* | *rpl16* | GTG | |
| *R. wallichiana* | *cox1* | | GGA |

Supplementary Table S8. The data size of 108 *Rhodiola* samples and the genes not assembled by Polypods.

| **SampleID** | **DataSize** | **GeneNotAssemble** |
|---|---|---|
| Rhodiola_tieghemii_XZ_1 | 3.9G | *rps13* |
| Rhodiola_fastigiata_XZ_1 | 5.2G | |
| Rhodiola_wallichiana_XZ | 4.1G | |
| Rhodiola_forrestii_YN | 3.4G | |
| Rhodiola_crenulata_XZ_1 | 3.6G | |
| Rhodiola_primuloides_YN_1 | 5.8G | |
| Rhodiola_kirilowii_YN_1 | 5.6G | |
| Rhodiola_wallichiana_var_cholaensis_YN | 4.8G | |
| Rhodiola_rosea_HB_1 | 2.9G | |
| Rhodiola_rosea_HB_2 | 3.4G | |
| Rhodiola_rosea_HB_3 | 3.4G | |
| Rhodiola_quadrifida_XZ_1 | 4.2G | |
| Rhodiola_bupleuroides_XZ_1 | 4.7G | |
| Rhodiola_sacra_var_tsuiana_XZ | 4.2G | |
| Rhodiola_kirilowii_XZ_1 | 5.0G | |
| Rhodiola_himalensis_XZ_1 | 4.3G | |
| Rhodiola_algida_XZ_1 | 3.8G | |
| Rhodiola_amabilis_XZ_1 | 4.5G | |
| Rhodiola_sacra_XZ_1 | 4.0G | |
| Rhodiola_atuntsuensis_XZ_1 | 4.0G | |
| Rhodiola_discolor_XZ | 6.7G | |
| Rhodiola_sacra_XZ_2 | 4.7G | |
| Rhodiola_algida_XZ_2 | 4.1G | |
| Rhodiola_serrata_XZ_1 | 4.1G | |

| | |
|---|---|
| Rhodiola_nepalica_XZ_1 | 3.3G |
| Rhodiola_purpureoviridis_XZ_1 | 3.8G |
| Rhodiola_atuntsuensis_XZ_3 | 3.1G |
| Rhodiola_crenulata_XZ_2 | 3.5G |
| Rhodiola_subopposita_XZ | 4.5G |
| Rhodiola_wallichiana_var_cholaensis_XZ_1 | 4.6G |
| Rhodiola_forrestii_XZ | 5.2G |
| Rhodiola_chrysanthemifolia_XZ_1 | 3.2G |
| Rhodiola_fastigiata_YN_1 | 4.3G |
| Rhodiola_fastigiata_XZ_2 | 5.0G |
| Rhodiola_kirilowii_YN_2 | 4.2G |
| Rhodiola_primuloides_YN_2 | 3.2G |
| Rhodiola_atuntsuensis_XZ_2 | 3.8G |
| Rhodiola_quadrifida_XZ_2 | 3.1G |
| Rhodiola_purpureoviridis_XZ_2 | 4.1G |
| Rhodiola_sexifolia_XZ | 3.5G |
| Rhodiola_tangutica_XZ_1 | 6.1G |
| Rhodiola_tibetica_XZ | 4.7G |
| Rhodiola_humilis_var_jialiensis_XZ | 3.5G |
| Rhodiola_kirilowii_XZ_2 | 4.3G |
| Rhodiola_hobsonii_XZ | 5.2G |
| Rhodiola_himalensis_XZ_2 | 4.4G |
| Rhodiola_sacra_XZ_3 | 2.9G |
| Rhodiola_daochengensis_XZ_1 | 4.8G |
| Rhodiola_prainii_XZ | 4.8G |
| Rhodiola_tangutica_XZ_2 | 802M |
| Rhodiola_sacra_XZ_4 | 4.3G |
| Rhodiola_heterodonta_XZ | 4.3G |
| Rhodiola_nepalica_XZ_2 | 2.2G |
| Rhodiola_serrata_XZ_2 | 4.6G |
| Rhodiola_bupleuroides_XZ_2 | 4.4G |
| Rhodiola_daochengensis_XZ_2 | 4.5G |
| Rhodiola_amabilis_XZ_2 | 4.5G |
| Rhodiola_crenulata_XZ_3 | 3.8G |
| Rhodiola_tieghemii_XZ_2 | 4.1G |
| Rhodiola_wallichiana_var_cholaensis_XZ_2 | 3.9G |
| Rhodiola_atuntsuensis_XZ_4 | 3.7G |
| Rhodiola_chrysanthemifolia_XZ_2 | 7.2G |
| Rhodiola_himalensis_XZ_3 | 3.5G |
| Rhodiola_nobilis_XZ | 4.3G |
| Rhodiola_kirilowii_YN_3 | 3.7G |
| Rhodiola_fastigiata_YN_2 | 3.1G |
| Rhodiola_kirilowii_YN_4 | 4.0G |
| Rhodiola_primuloides_YN_3 | 4.5G |
| Rhodiola_yunnanensis_YN_1 | 3.1G |
| Rhodiola_alsia_SC | 4.0G |
| Rhodiola_fastigiata_SC_1 | 4.0G |
| Rhodiola_fastigiata_SC_2 | 4.1G |
| Rhodiola_purpureoviridis_SC | 4.5G |

| | | |
|---|---|---|
| Rhodiola_discolor_SC | 3.1G | |
| Rhodiola_fastigiata_SC_3 | 4.0G | |
| Rhodiola_macrocarpa_SC | 669M | |
| Rhodiola_yunnanensis_YN_2 | 3.7G | |
| Rhodiola_dumulosa_NX | 4.8G | |
| Rhodiola_quadrifida_QH | 3.3G | |
| Rhodiola_algida_QH_2 | 4.1G | |
| Rhodiola_kirilowii_QH | 4.4G | |
| Rhodiola_rosea_HB_4 | 4.1G | |
| Rhodiola_kirilowii_HB | 5.0G | |
| Rhodiola_crenulata_SC | 6.0G | |
| Rhodiola_sacra_XZ_5 | 3.0G | *rpl5* |
| Rhodiola_sacra_YN | 5.6G | |
| Rhodiola_quadrifida_XZ_3 | 4.4G | |
| Rhodiola_dumulosa_SX_1 | 5.0G | |
| Rhodiola_dumulosa_SX_2 | 3.2G | |
| Rhodiola_kirilowii_SX_2 | 2.7G | |
| Rhodiola_angusta_JL_2 | 5.9G | |
| Rhodiola_sachalinensis_JL_1 | 4.1G | |
| Rhodiola_rosea_HB_5 | 3.5G | |
| Rhodiola_rosea_HB_6 | 2.8G | |
| Rhodiola_fastigiata_XJ_1 | 4.1G | |
| Rhodiola_fastigiata_XJ_2 | 6.9G | |
| Rhodiola_crenulata_XJ | 3.8G | |
| Rhodiola_litwinowii_XJ | 6.4G | |
| Rhodiola_rosea_XJ_1 | 2.8G | |
| Rhodiola_rosea_XJ_2 | 4.4G | |
| Rhodiola_himalensis_XZ_4 | 5.6G | |
| Rhodiola_angusta_JL_3 | 2.7G | |
| Rhodiola_rosea_JL | 3.7G | |
| Rhodiola_rosea_JL_2 | 3.6G | |
| Rhodiola_crenulata_AG | 2.9G | |
| Rhodiola_chrysanthemifolia_AG | 5.0G | |
| Rhodiola_fastigiata_AG | 4.1G | |
| Rhodiola_yunnanensis_YN_3 | 4.9G | |

Supplementary Table S9. Overall comparison of PMGA and RhoMitoAnnotator annotations across seven *Rhodiola* mitogenomes.

| **Species** | **Size (bp)** | **PMGA PCGs** | **Rho PCGs** | **PCG union** | **PMGA recovery** | **Rho recovery** | **Shared PCGs** | **Boundary match** | **Protein match** | **Exact match** |
|---|---|---|---|---|---|---|---|---|---|---|
| *R. juparensis* | 202,019 | 31 | 29 | 32 | 31/32, 96.9% | 29/32, 90.6% | 28 | 23/28, 82.1% | 19/28, 67.9% | 18/28, 64.3% |
| *R. crenulata* | 194,106 | 24 | 22 | 24 | 24/24, 100.0% | 22/24, 91.7% | 22 | 18/22, 81.8% | 17/22, 77.3% | 16/22, 72.7% |

| | | | | | | | | | | |
|---|---|---|---|---|---|---|---|---|---|---|
| *R. sacra* | 128,593 | 11 | 11 | 13 | 11/13, 84.6% | 11/13, 84.6% | 9 | 6/9, 66.7% | 7/9, 77.8% | 6/9, 66.7% |
| *R. tangutica* | 257,378 | 32 | 30 | 33 | 32/33, 97.0% | 30/33, 90.9% | 29 | 23/29, 79.3% | 20/29, 69.0% | 19/29, 65.5% |
| *R. kirilowii* | 79,921 | 13 | 12 | 13 | 13/13, 100.0% | 12/13, 92.3% | 12 | 8/12, 66.7% | 8/12, 66.7% | 8/12, 66.7% |
| *R. rosea* | 259,150 | 30 | 30 | 31 | 30/31, 96.8% | 30/31, 96.8% | 29 | 23/29, 79.3% | 22/29, 75.9% | 21/29, 72.4% |
| *R. wallichiana* | 82,073 | 14 | 13 | 15 | 14/15, 93.3% | 13/15, 86.7% | 12 | 9/12, 75.0% | 10/12, 83.3% | 8/12, 66.7% |
| Total | — | — | — | — | — | — | 141 | 110/141, 78.0% | 103/141, 73.0% | 96/141, 68.1% |

**Note:** PCGs, protein-coding genes; Rho, RhoMitoAnnotator. PCG union refers to the non-redundant set of PCGs annotated by either tool. Recovery was calculated using the PCG union as the denominator. Boundary match indicates identical CDS/exon coordinates between the two tools. Protein match indicates identical translated protein sequences after excluding terminal stop symbols. Exact match indicates shared PCGs with both identical coordinates and identical translated protein sequences.

Supplementary Table S10. Major annotation differences between PMGA and RhoMitoAnnotator in seven *Rhodiola* mitogenomes.

| **Species** | **PMGA-specific CDSs** | **Rho-specific CDSs** | **Boundary-different PCGs** | **Protein-different PCGs** |
|---|---|---|---|---|
| *R. juparensis* | *rps4, sdh3, sdh4* | *atp9* | *ccmFn, mttB, nad6, nad7, rpl16* | *atp6, atp8, ccmFn, cob, mttB, nad1, nad4L, nad6, rpl16* |
| *R. crenulata* | *sdh3, sdh4* | None | *cox2, matR, mttB, nad7* | *atp6, cox2, matR, mttB, nad4L* |
| *R. sacra* | *rps14, sdh4* | *atp9, ccmB* | *cob, mttB, nad7* | *cob, mttB* |
| *R. tangutica* | *rps14, rps4, sdh4* | *atp9* | *ccmFn, cob, mttB, nad6, nad7, rpl16* | *atp6, atp8, ccmFn, cob, mttB, nad1, nad4L, nad6, rpl16* |
| *R. kirilowii* | *sdh4* | None | *atp1, atp6, nad6, nad7* | *atp1, atp6, nad6, nad7* |
| *R. rosea* | *sdh4* | *atp9* | *cob, matR, mttB, nad1, nad7, rpl16* | *atp6, cob, matR, mttB, nad1, nad4L, rpl16* |
| *R. wallichiana* | *rps4, sdh4* | *atp9* | *cob, cox1, nad7* | *atp6, cob* |

Supplementary Table S11. SNP counts across 108 samples of 39 *Rhodiola* species. *nad6* analyzed only within its first 619 bp due to downstream sequence variation.

| **Genes** | **Number of SNPs** |
|---|---|
| *atp1* | 23 |
| *atp4* | 7 |
| *atp6* | 11 |

| | |
|---|---|
| *atp8* | 24 |
| *atp9* | 0 |
| *ccmB* | 6 |
| *ccmC* | 6 |
| *ccmFc* | 18 |
| *ccmFn* | 13 |
| *cob* | 8 |
| *cox1* | 6 |
| *cox2* | 4 |
| *cox3* | 6 |
| *matR* | 32 |
| *mttB* | 10 |
| *nad1* | 14 |
| *nad2* | 7 |
| *nad3* | 2 |
| *nad4* | 16 |
| *nad4L* | 2 |
| *nad5* | 12 |
| *nad6* | 9 |
| *nad7* | 2 |
| *nad9* | 8 |
| *rpl10* | 1 |
| *rpl16* | 13 |
| *rpl5* | 6 |
| *rps12* | 9 |
| *rps13* | 6 |
| *rps7* | 5 |

Supplementary Table S12. The location information of 108 *Rhodiola* samples.

| **Latin name** | **Location** |
|---|---|
| Rhodiola_tieghemii_XZ_1 | Bayi District, Linzhi City, Tibet |
| Rhodiola_fastigiata_XZ_1 | Tibet |
| Rhodiola_wallichiana_XZ | Bayi District, Linzhi City, Tibet |
| Rhodiola_forrestii_YN | Lijiang City, Yunnan |
| Rhodiola_crenulata_XZ_1 | Gongbu Jiangda County, Linzhi City, Tibet |
| Rhodiola_primuloides_YN_1 | Jianchuan County, Dali Prefecture, Yunnan |
| Rhodiola_kirilowii_YN_1 | Shangri-La City, Diqing Prefecture, Yunnan |
| Rhodiola_wallichiana_var_cholaensis_YN | Deqin County, Diqing Prefecture, Yunnan |
| Rhodiola_rosea_HB_1 | Dadanzi Township, Fengning County, Hebei |
| Rhodiola_rosea_HB_2 | Dadanzi Township, Fengning County, Hebei |
| Rhodiola_rosea_HB_3 | Dadanzi Township, Fengning County, Hebei |
| Rhodiola_quadrifida_XZ_1 | Zangbaka, Mangkang County, Qamdo City, Tibet |
| Rhodiola_bupleuroides_XZ_1 | Zangbaka, Mangkang County, Qamdo City, Tibet |
| Rhodiola_sacra_var_tsuiana_XZ | Chamang Highway, Chaya County, Qamdo City, Tibet |

| | |
|---|---|
| Rhodiola_kirilowii_XZ_1 | Chamang Highway, Chaya County, Qamdo City, Tibet |
| Rhodiola_himalensis_XZ_1 | Bazongwa, Chaya County, Qamdo City, Tibet |
| Rhodiola_algida_XZ_1 | Jiegar, Leiniaoqi County, Qamdo City, Tibet |
| Rhodiola_amabilis_XZ_1 | Chidou Town, Dingqing County, Qamdo City, Tibet |
| Rhodiola_sacra_XZ_1 | Reshapu, Suo County, Nagqu City, Tibet |
| Rhodiola_atuntsuensis_XZ_1 | Resha, Rongbu Town, Suo County, Nagqu City, Tibet |
| Rhodiola_discolor_XZ | Bibu County, Nagqu City, Tibet |
| Rhodiola_sacra_XZ_2 | Zhenri, Gonggar County, Shannan City, Tibet |
| Rhodiola_algida_XZ_2 | Langkazi County, Shannan City, Tibet |
| Rhodiola_serrata_XZ_1 | Xingna, Qusong County, Shannan City, Tibet |
| Rhodiola_nepalica_XZ_1 | Xingna, Qusong County, Shannan City, Tibet |
| Rhodiola_purpureoviridis_XZ_1 | Jiongbuqiong, Qusong County, Shannan City, Tibet |
| Rhodiola_atuntsuensis_XZ_3 | Chapu, Gongbujiangda County, Nyingchi City, Tibet |
| Rhodiola_crenulata_XZ_2 | Chapu, Gongbujiangda County, Nyingchi City, Tibet |
| Rhodiola_subopposita_XZ | Chapu, Gongbujiangda County, Nyingchi City, Tibet |
| Rhodiola_wallichiana_var_cholaensis_XZ_1 | National Highway 318, Linzhi Town, Bayi District, Linzhi City, Tibet |
| Rhodiola_forrestii_XZ | Sejila Mountain, Bayi District, Nyingchi City, Tibet |
| Rhodiola_chrysanthemifolia_XZ_1 | Bayi District, Linzhi City, Tibet |
| Rhodiola_fastigiata_YN_1 | National Highway 214, Shengping Town, Deqin County, Diqing Tibetan Autonomous Prefecture, Yunnan |
| Rhodiola_fastigiata_XZ_2 | Zuogong County, Qamdo City, Tibet |
| Rhodiola_kirilowii_YN_2 | Deqin County, Diqing Tibetan Autonomous Prefecture, Yunnan |
| Rhodiola_primuloides_YN_2 | Eryuan County, Dali Bai Autonomous Prefecture, Yunnan |
| Rhodiola_atuntsuensis_XZ_2 | Mugabu, Langkazi County, Shannan City, Tibet |
| Rhodiola_quadrifida_XZ_2 | Zangbaka, Mangkang County, Qamdo City, Tibet |
| Rhodiola_purpureoviridis_XZ_2 | Chaya Highway, Xiangdui Town, Chaya County, Qamdo City, Tibet |
| Rhodiola_sexifolia_XZ | Zhongji, Chaya County, Qamdo City, Tibet |
| Rhodiola_tangutica_XZ_1 | Leiwuqi County, Qamdo City, Tibet |
| Rhodiola_tibetica_XZ | National Highway 317, Dingqing County, Qamdo City, Tibet, goes directly to Dhaka |
| Rhodiola_humilis_var_jialiensis_XZ | Shangshang Village, Dingqing County, Qamdo City, Tibet |
| Rhodiola_kirilowii_XZ_2 | Bado Middle Bridge, Dingqing County, Qamdo City, Tibet |
| Rhodiola_hobsonii_XZ | Bado Middle Bridge, Dingqing County, Qamdo City, Tibet |
| Rhodiola_himalensis_XZ_2 | Chaga Bridge (Hala Bridge), Suo County, Nagqu City, Tibet |
| Rhodiola_sacra_XZ_3 | Entrance of G6 Beijing-Tibet Expressway (Nakqu direction), Doulungdeqing District, Lhasa City, Tibet |
| Rhodiola_daochengensis_XZ_1 | Entrance of G6 Beijing-Tibet Expressway (Nakqu direction), Doulungdeqing District, Lhasa City, Tibet |
| Rhodiola_prainii_XZ | Entrance of G6 Beijing-Tibet Expressway (Nakqu direction), Doulungdeqing District, Lhasa City, Tibet |

| | |
|---|---|
| Rhodiola_tangutica_XZ_2 | Entrance of G6 Beijing-Tibet Expressway (Nakqu direction), Doulungdeqing District, Lhasa City, Tibet |
| Rhodiola_sacra_XZ_4 | National Highway 349, Gonggar County, Shannan City, Tibet |
| Rhodiola_heterodonta_XZ | Bori, Xiajiang Township, Qusong County, Shannan City, Tibet |
| Rhodiola_nepalica_XZ_2 | Bori, Qusong County, Shannan City, Tibet |
| Rhodiola_serrata_XZ_2 | Xingna, Xiajiang Township, Qusong County, Shannan City, Tibet |
| Rhodiola_bupleuroides_XZ_2 | Lalong Village Bridge, G4218 Linla Highway, Mozhugongka County, Lhasa City, Tibet |
| Rhodiola_daochengensis_XZ_2 | National Highway 318, Tashigang Township, Mozhugongka County, Lhasa City, Tibet |
| Rhodiola_amabilis_XZ_2 | Mozhugongkar County, Lhasa City, Tibet |
| Rhodiola_crenulata_XZ_3 | Chapu, Gongbujiangda County, Nyingchi City, Tibet |
| Rhodiola_tieghemii_XZ_2 | Zhangba Village, Bayi District, Nyingchi City, Tibet |
| Rhodiola_wallichiana_var_cholaensis_XZ_2 | Chagang'er, Bayi District, Nyingchi City, Tibet |
| Rhodiola_atuntsuensis_XZ_4 | National Highway 318, Linzhi Town, Bayi District, Linzhi City, Tibet |
| Rhodiola_chrysanthemifolia_XZ_2 | National Highway 318, Lulang Town, Bayi District, Nyingchi City, Tibet |
| Rhodiola_himalensis_XZ_3 | Class 21, Zuogong County, Qamdo City, Tibet |
| Rhodiola_nobilis_XZ | National Highway 318, Lulang Town, Bayi District, Nyingchi City, Tibet |
| Rhodiola_kirilowii_YN_3 | National Highway 214, Shengping Town, Deqin County, Diqing Tibetan Autonomous Prefecture, Yunnan |
| Rhodiola_fastigiata_YN_2 | National Highway 214, Shengping Town, Deqin County, Diqing Tibetan Autonomous Prefecture, Yunnan |
| Rhodiola_kirilowii_YN_4 | National Highway 214, Shengping Town, Deqin County, Diqing Tibetan Autonomous Prefecture, Yunnan |
| Rhodiola_primuloides_YN_3 | Eryuan County, Dali Bai Autonomous Prefecture, Yunnan |
| Rhodiola_yunnanensis_YN_1 | Bai Exit, Yousuo Town, Eryuan County, Dali Bai Autonomous Prefecture, Yunnan |
| Rhodiola_alsia_SC | Yala Pass, Provincial Road 434, Kangding City, Garze Tibetan Autonomous Prefecture, Sichuan |
| Rhodiola_fastigiata_SC_1 | Jianziwanshan Tunnel, Yajiang County, Garze Tibetan Autonomous Prefecture, Sichuan |
| Rhodiola_fastigiata_SC_2 | Twelve-kilometer road class in Xinduqiao Town, Kangding City, Garze Tibetan Autonomous Prefecture, Sichuan |
| Rhodiola_purpureoviridis_SC | Litang County, Garze Tibetan Autonomous Prefecture, Sichuan |
| Rhodiola_discolor_SC | Zhabu, Litang County, Garze Tibetan Autonomous Prefecture, Sichuan |
| Rhodiola_fastigiata_SC_3 | Litang County, Garze Tibetan Autonomous Prefecture, Sichuan |
| Rhodiola_macrocarpa_SC | Ranwu Township, Xiangcheng County, Garze Tibetan Autonomous Prefecture, Sichuan |
| Rhodiola_yunnanensis_YN_2 | Liangwang Mountain, Chenggong District, Kunming City, Yunnan |
| Rhodiola_dumulosa_NX | Sandaogou, Helan Mountain, Yinchuan, Ningxia |
| Rhodiola_quadrifida_QH | Daban Mountain, Menyuan, Haibei Prefecture, Qinghai |
| Rhodiola_algida_QH_2 | Banma County, Zhuluo Prefecture, Qinghai |
| Rhodiola_kirilowii_QH | Datong County, Qinghai |
| Rhodiola_rosea_HB_4 | Weichang, Hebei |

| | |
|---|---|
| Rhodiola_kirilowii_HB | Weichang, Hebei |
| Rhodiola_crenulata_SC | Three peaks in Changpinggou Scenic Area, Siguniangshan Town, Xiaojin County, Aba Prefecture, Sichuan |
| Rhodiola_sacra_XZ_5 | Mozhugongka County, Lhasa City, Tibet |
| Rhodiola_sacra_YN | Yunnan |
| Rhodiola_quadrifida_XZ_3 | Qamdo District, Tibet |
| Rhodiola_dumulosa_SX_1 | Taibai Mountain, Shaanxi |
| Rhodiola_dumulosa_SX_2 | Taibai Mountain, Shaanxi |
| Rhodiola_kirilowii_SX_2 | Taibai Mountain, Shaanxi |
| Rhodiola_angusta_JL_2 | Tonghua, Jilin |
| Rhodiola_sachalinensis_JL_1 | Tonghua, Jilin |
| Rhodiola_rosea_HB_5 | Weichang, Hebei |
| Rhodiola_rosea_HB_6 | Weichang, Hebei |
| Rhodiola_fastigiata_XJ_1 | Xinjiang |
| Rhodiola_fastigiata_XJ_2 | Xinjiang |
| Rhodiola_crenulata_XJ | Xinjiang |
| Rhodiola_litwinowii_XJ | Taxkorgan Tajik Autonomous County, Kashgar District, Xinjiang |
| Rhodiola_rosea_XJ_1 | Xinjiang |
| Rhodiola_rosea_XJ_2 | Taxkorgan Tajik Autonomous County, Kashgar District, Xinjiang |
| Rhodiola_himalensis_XZ_4 | Bomi County, Linzhi City, Tibet |
| Rhodiola_angusta_JL_3 | Erdaobaihe Town, Antu County, Yanbian Korean Autonomous Prefecture, Jilin |
| Rhodiola_rosea_JL | Laobai Mountain, Dunhua City, Yanbian Korean Autonomous Prefecture, Jilin |
| Rhodiola_rosea_JL_2 | Erdaobaihe Town, Antu County, Yanbian Korean Autonomous Prefecture, Jilin |
| Rhodiola_crenulata_AG | Anguo medicinal materials market, Baoding, Hebei |
| Rhodiola_chrysanthemifolia_AG | Anguo medicinal materials market, Baoding, Hebei |
| Rhodiola_fastigiata_AG | Anguo medicinal materials market, Baoding, Hebei |
| Rhodiola_yunnanensis_YN_3 | Kunming, Yunnan |

Supplementary Table S13. Primers designed for amplifying the 5’-end variant regions of *nad9* and *ccmFn* in *R. crenulata* and *ccmC* in *R. rosea*.

| **Genes** | **Primer sequences** |
|---|---|
| ccmC-F | GGTTCGCAGCTAAGCAAGAT |
| ccmC-R | AGTGGTGCTATCCGAAGACT |
| ccmFn-F | ACAAGTGGTGAAAGAAGCCA |
| ccmFn-R | AATGCAAGGAAAAGACCCGG |
| nad9-F | GTGTTGTACCACCATTCCGC |
| nad9-R | TCCCATGTTCCGATCTTTCCA |
